# Supplementary material for: Identification and Evaluation of Dibasic Piperidines as Cell Wall Inhibitors against Mycobacterium tuberculosis
Source: ACS Infect Dis. 2026 May 14;12(6):2004–14. doi: 10.1021/acsinfecdis.6c00123 (PMC13270526; doi:10.1021/acsinfecdis.6c00123)

Supporting Information

Identification and Evaluation of dibasic piperidines as cell wall inhibitors against *Mycobacterium tuberculosis*

Authors

Claire Naylor[1], Gareth Prosser[1], Tracy Bayliss[1], Lila F Berle[2], Joshua B Wallach[3], Heather Kim[3], Rodrigo Aguilera Olvera[3], Stephen Thompson[1], Thomas R Ioerger[4], Laura Simpson[1], Ruth Casanueva[5], Laura Guijarro-Lopez[5], Kevin D. Read[1], Paul G Wyatt[1], Dirk Schnappinger[3], Clifton E Barry[2], Simon R Green[1], Helena I M Boshoff[2], Laura A T Cleghorn[1]*

Affiliations

[1]Drug Discovery Unit, Faculty of Life Sciences, University of Dundee, Dundee, DD1 5EH, UK

[2]Tuberculosis Research Section, Laboratory of Clinical Immunology and Microbiology, NIAID, NIH, 9000 Rockville Pike, Bethesda, Maryland, MD 20892, USA

[3]Dept. of Microbiology and Immunology, Weill Cornell Medical College, New York, NY 10065, USA

[4]Department of Computer Science and Engineering, Texas A&M University, College Station, TX 77843, USA

[5]Global Health Medicines R&D, GlaxoSmithKline, Severo Ochoa 2, Tres Cantos, 28760, Madrid, Spain

***Corresponding author**

l.a.t.cleghorn@dundee.ac.uk

ORCHID iD 0000-0001-6218-0092

**Table of Contents**	Page
Table S1: Modifications on R_1	S2
Table S2: Compound **1** resistant mutants	S3
Figure S1: Compound **1** targets cell wall biosynthesis	S3
General Chemistry Methods	S4
Chemistry Experimental Procedures	S4
1H and 13 C NMR Analytical data compounds 1-32	S26

Table S1: R_1Modifications

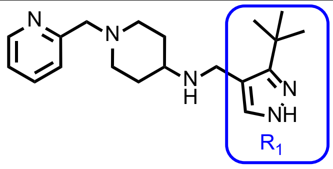

Compound	R_1	*H37Rv* MIC[A] (µM)	*H37Rv* MIC[B] (µM)	Compound	R_1	*H37Rv* MIC[A] (µM)	*H37Rv* MIC[B] (µM)
**1**	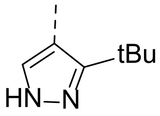	**3.2**	**3.6**	**7**	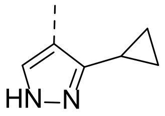	**37**	**>50**
**2**	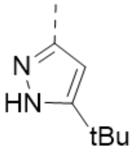	**>50**	**>50**	**8**	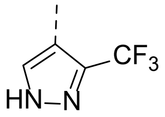	**>50**	**>50**
**3**	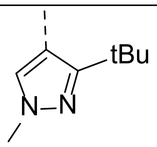	**>50**	**>50**	**9**	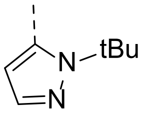	**>50**	**>50**
**4**	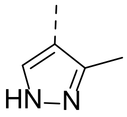	**>50**	**>50**	**10**	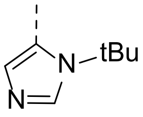	**>50**	**>50**
**5**	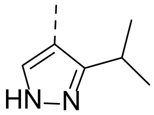	**25**	**25**	**11**	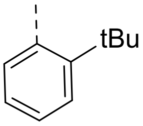	**>50**	**>50**
**6**	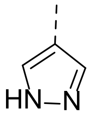	**>50**	**>50**				

[a]MIC is the minimum concentration required to inhibit the growth of *M. tuberculosis* (H37Rv) in liquid culture by 90% compared to untreated control (MIC[A] 7H9/DPPC/CAS/Tx media, MIC[B] 7H9/GLU/CAS/Tx media);

Table S2: Resistant mutants

Strain	Raised against compound	2 wk MIC (7H9 ADC Tw)	Fold-resistance	Mutations
WT		1 - 2.3	-	-
A1	1	37	16	ubiA:S173P
B5	1	9.4	4	embB:D300G
C5	1	37	16	embC:T348A
WT		21 - 0.2	-	
B7	21	1.6	8	ubiA:S173P

MIC required to inhibit the growth of *M. tuberculosis* in liquid culture (7H9/GLU/GLY/Tween). The MIC is shown for wild type H37Rv and each of the strains resistant to **1** or **21**. The fold resistance is also shown along with the SNP identified through whole genome sequencing.

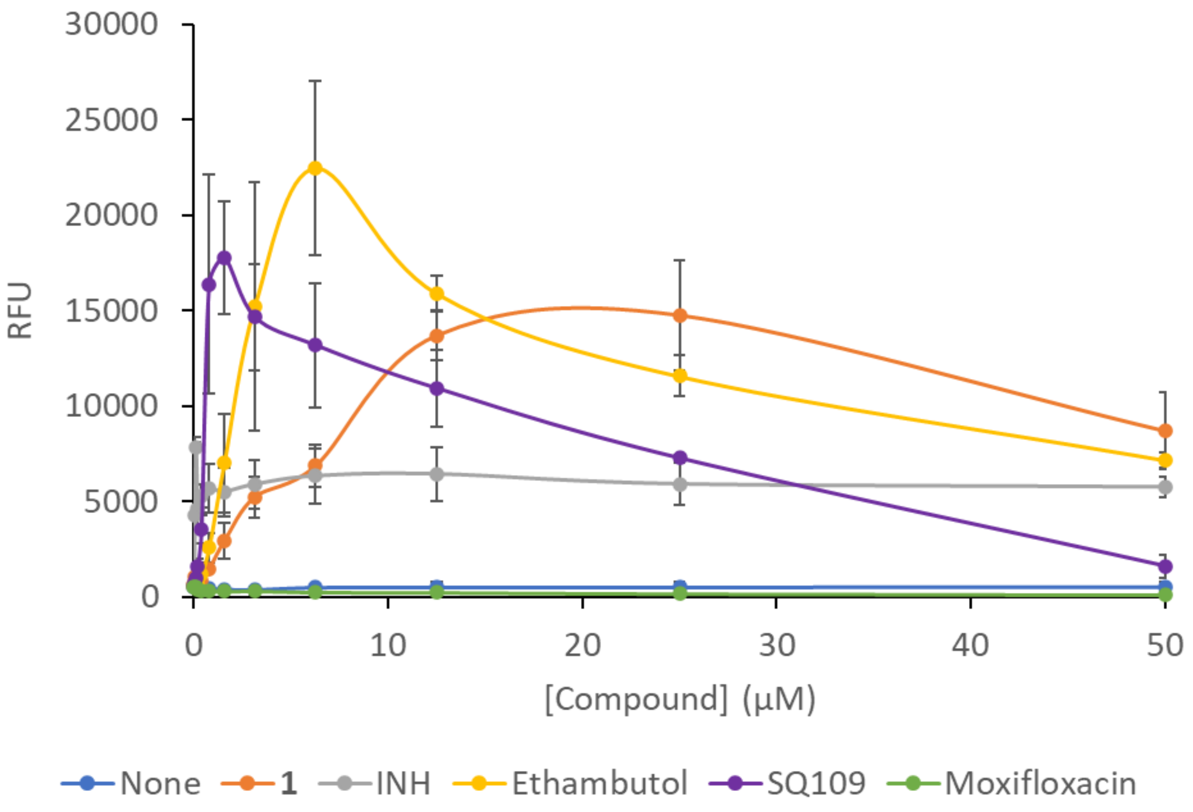

Figure S1. Compound 1 targets cell wall biosynthesis H37Rv strain transfected with the Pini-LUX reporter vector was treated with compound as previously reported{Naran, 2016 #265}. Luminescence was monitored at 4 different timepoints, data shown for the 48h timepoint. The relative fluorescence units are shown for the strain treated with DMSO, **1**, INH, Ethambutol, SQ109 (positive controls) and moxifloxacin (negative control). Experiments were performed in duplicate.

General Chemistry methods: Normal phase TLCs were carried out on pre-coated silica plates (Kieselgel 60 F254, BDH) with visualization via U.V. light (UV254/365 nm) and/or potassium permanganate solution. Normal phase flash chromatography was performed using Combiflash Companion Rf (commercially available from Teledyne ISCO) and prepacked silica gel columns purchased from Teledyne ISCO. Mass-directed preparative HPLC separations and preparative HPLC chromatographic separations were conducted with either Waters XBridge C18 columns, 100 * 19 mm, 5 µm particle size or Waters X bridge 150 * 25 mm * 5 µm .^{1}H NMR spectra were recorded on a Bruker Avance DPX 500 spectrometer (^{1}H at 500.1 MHz), or a Bruker Avance DPX 400 (^{1}H at 400 MHz) or 500MHz Cryo NMR (Bruker, Avance 3). Chemical shifts (δ) are expressed in ppm recorded using the residual solvent as the internal reference in all cases. Signal splitting patterns are described as singlet (s), doublet (d), triplet (t), quartet (q), multiplet (m), broad (br), or a combination thereof. Coupling constants (J) are quoted to the nearest 0.1 Hz. High-resolution electrospray measurements were performed on a Bruker Daltonics MicroTOF mass spectrometer or on an Orbitrap Exploris 120 Mass spectrometer. Low resolution electrospray (ES) mass spectra were recorded on an Advion Compact Mass Spectrometer (CMS; model ExpressIon CMS) connected to Dionex Ultimate 3000 UPLC system with diode array detector. HPLC chromatographic separations were conducted using a Waters XBridge C18 column, 2.1 x 50 mm, 3.5 µm particle size or Waters XSelect 2.1 x 30 mm, 2.5 µm particle size. The compounds were eluted with a gradient of 5 to 95 % acetonitrile/water + 0.1 % Ammonia or + 0.1 % formic acid. Unless otherwise stated herein reactions have not been optimized. Solvents and reagents were purchased from commercial suppliers and used without further purification. Dry solvents were purchased in sure sealed bottles stored over molecular sieves. All final compounds showed chemical purity of ≥ 95 % as determined from the UV chromatogram (190−450 nm) obtained by LC-MS analysis (unless otherwise stated). Note that due to the tautomeric forms of the NH-pyrazoles within this series of compounds, the signals for these cannot always be observed within the ^{13}C spectra and have therefore not been reported in these cases.

Experimental Procedures:

General procedures

General procedure 1: NaOAc (2.0 equiv.) was added to a mixture of amine (1.0 equiv. HCl) in EtOH and the reaction stirred at room temperature for 10 min, the desired aldehyde (1.2 equiv.), AcOH (0.13 equiv.) and $NaBH_3CN$ (2.6 equiv.) was added and the reaction stirred for 16 h, quenched with water and concentrated *in vacuo*. The residue was purified by preparative HPLC (10-40 % CH_3CN in water (+1 % NH_4HCO_3)) to afford **compounds 1-3.**

General procedure 2: To a solution of amine (1.0 equiv.) in DCM and AcOH (0.1 equiv.) was added the desired aldehyde (1.0 equiv.) followed by $NaBH(OAc)_3$ (1.2 equiv.) and the reaction stirred at room temperature for 16 h, diluted with MeOH and transferred to SCX SPE cartridge, washed with MeOH and eluted with 3M NH_3 in MeOH. The residue was purified by reverse phase flash column chromatography (5-95 % CH_3CN in water (+0.1 % NH_4OH) collecting at 220 nm) to afford **compounds 6-11** and **27-30.**

General procedure 3: To a solution of desired aldehyde (1.0 equiv.) and amine (1.0 equiv.) in MeOH was added AcOH (0.1 equiv.) and $NaBH_3CN$ (2 equiv.) and the reaction stirred at room temperature for 12 h, quenched with water, the pH adjusted to ~8 with saturated, aqueous $NaHCO_3$ solution and extracted with EtOAc. The combined organic layers were washed with brine, dried over anhydrous Na_2SO_4, filtered and concentrated in vacuo to afford **compounds 4, 5, 12-20** and **31-32.**

General procedure 4: To a solution of Boc intermediate (1.0 equiv.) in CH_3CN was added HCl in dioxane (4 M, 14.0 *eq*uiv.). The reaction mixture stirred at room temperature for 0.5 h, concentrated in vacuo to afford intermediates on route to **compounds 12-19.**

General procedure 5: To a solution of amine (1.0 equiv.) in THF/EtOH was added the desired aldehyde (1.0 equiv.) 3Å molecular sieves and AcOH (0.1 equiv.). After 1 h $NaBH(OAc)_3$ (2 equiv.) was added and the reaction stirred at room temperature for 16 h. the pH was adjusted to ~7 with aqueous $NaHCO_3$, filtered and the filtrate concentrated in vacuo. The crude material was purified by preparative HPLC conditions to afford **compounds 21-26.**

***tert*-butyl (1-(pyridin-2-ylmethyl)piperidin-4-yl) carbamate (Intermediate 1, 33)**

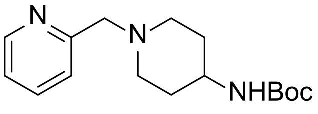

To a solution of *tert*-butyl piperidin-4-ylcarbamate (31 g, 154 mmol, 1.0 equiv.) and picolinaldehyde (17.4 g, 163 mmol, 1.1 equiv.) and AcOH in MeOH (886 µl, 15.5 mmol, 0.1 equiv., 0.5 M) at rt under N_2 was added $NaBH(OAc)_3$ (65.6 g, 310 mmol, 2.0 equiv.) in one portion and the reaction stirred for 12 h, concentrated, diluted with water and extracted with DCM. The combined organic layers were washed with brine, dried over Na_2SO_4, filtered and concentrated *in vacuo*. The residue was purified by reverse phase flash column chromatography (5-95 % CH_3CN in water (+0.1 % FA)) to afford *tert*-butyl (1-(pyridin-2-ylmethyl)piperidin-4-yl) carbamate (31.6 g, 107.8 mmol, 70 %) as white solid. **LCMS** (ES+): *m/z* 292.2 $[M+H]^+$, 100 %.

1-(pyridin-2-ylmethyl)piperidin-4-amine HCl (34.HCl)

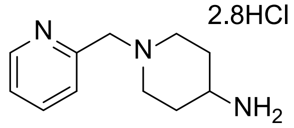

To a solution of *tert*-butyl (1-(pyridin-2-ylmethyl)piperidin-4-yl)carbamate (31.6 g, 108 mmol, 1.0 equiv.) in CH_3CN (0.36 M) was added 4M HCl in dioxane (11 equiv.) dropwise at 0 °C under N_2. The reaction mixture stirred at 10 °C for 1 h, concentrated *in vacuo* and the residue triturated with CH_3CN and filtered to afford **34.HCl** (30.5 g, 98.3 mmol, 91 %, 2.8 HCl) as an off-white solid. **^{1}H NMR** (400 MHz, DMSO-d_6) δ 8.93 - 8.81 (m, 1H), 8.56 - 8.46 (m, 1H), 8.12 (d, *J* = 7.6 Hz, 1H), 8.05 - 7.97 (m, 1H), 4.72 (s, 2H), 3.72 (m, 2H), 3.65 - 3.54 (m, 1H), 3.36 (td, *J* = 2.8, 12.8 Hz, 2H), 2.34 (m, 2H), 2.07 - 1.91 (m, 2H). **LCMS** (ES+): *m/z* 192.1 $[M+H]^+$, 100 %.

1-(pyridin-2-ylmethyl)piperidin-4-amine, 34

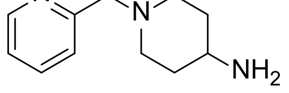

1-(2-pyridylmethyl)piperidin-4-amine hydrochloride **33.HCl** (3.73 g, 16.4 mmol) was dissolved in DCM loaded onto a preconditioned SCX cartridge, washed with DCM, DCM/MeOH and MeOH then eluted with 3M NH_3 in MeOH to afford **R1** (2.14 g, 11.2 mmol, 65 %) as a light yellow oil. **^{1}H NMR** (500 MHz, MeOD) δ 8.49 (d, *J* = 4.3 Hz, 1H), 7.85 - 7.81 (m ,1H), 7.56 - 7.54 (m, 1H), 7.35 - 7.32 (m, 1H), 3.66 (s, 2H), 2.91 - 2.86 (m, 2H), 2.73 - 2.65 (m, 1H), 2.21 - 2.14 (m, 2H), 1.87 - 1.81 (m, 2H), 1.53 - 1.43 (m, 2H). **LCMS** (ES+): *m/z* 191.9 $[M+H]^+$, 100 %.

***tert*-butyl 4-(((3-(tert-butyl)-1*H*-pyrazol-4-yl)methyl)amino)piperidine-1-carboxylate (35)**

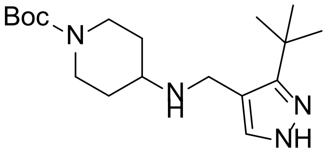

To a solution of *tert*-butyl 4-aminopiperidine-1-carboxylate (5.5 g, 27.46 mmol, 1.0 equiv.), and 3-(*tert*-butyl)-1*H*-pyrazole-4-carbaldehyde (3.76 g, 24.72 mmol, 0.9 equiv.) in MeOH (0.5 M) was added AcOH (157 µL, 2.75 mmol, 0.1 equiv.) in one portion at rt under N_2. The reaction mixture was stirred for 0.5 h, $NaBH_3CN$ (3.45 g, 54.9 mmol, 2.0 equiv.) added and stirred for a further 12 h. The reaction mixture was quenched with water and extracted with EtOAc. The combined organic layers were washed with brine, dried over Na_2SO_4, filtered and concentrated *in vacuo* and purified by reverse phase flash column chromatography (5-95 % CH_3CN in water (+0.1 % FA)) to afford *tert*-butyl 4-(((3-(*tert*-butyl)-1*H*-pyrazol-4-yl)methyl)amino)piperidine-1-carboxylate (9.2 g, 27.3 mmol, 99 % , FA salt) as a white solid. **LCMS** (ES+): *m/z* 337.3 $[M+H]^+$, 85 %.

***N*-((3-(*tert*-butyl)-1*H*-pyrazol-4-yl)methyl)piperidin-4-amine HCl, (36.HCl)**

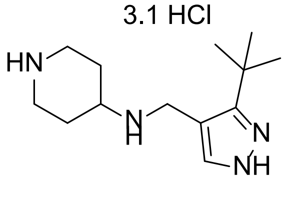

To a solution of tert-butyl 4-(((3-(*tert*-butyl)-1*H*-pyrazol-4-yl)methyl)amino)piperidine-1-carboxylate (9.2 g, 27.34 mmol, FA salt, 1 equiv.) in CH_3CN (0.3 M) was added HCl in dioxane (4 M, 13.2 equiv.) in one portion at 0 °C under N_2. The mixture was stirred at 0 °C for 1 h. The reaction mixture was concentrated *in vacuo* and the residue triturated with CH_3CN to afford *N*-((3-(*tert*-butyl)-1*H*-pyrazol-4-yl)methyl)piperidin-4-amine HCl as a white solid (6.41 g, 17.7 mmol, 3.1 HCl, 65 %). **^{1}H NMR (**400 MHz, D_2O) δ 8.16 (s, 1H), 4.44 (s, 2H), 3.74 - 3.65 (m, 1H), 3.64 - 3.54 (m, 2H), 3.18 - 3.06 (m, 2H), 2.51 – 5.43 (m, 2H), 2.04 - 1.93 (m, 2H), 1.38 (s, 9H). **LCMS** (ES+): m/z 237.2 $[M+H]^+$, 96 %.

***N*-((3-(*tert*-butyl)-1*H*-pyrazol-4-yl)methyl)piperidin-4-amine, 36**

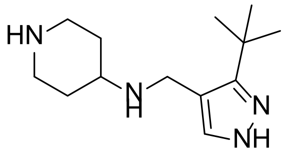

Synthesized following the same procedure as **R1**, from *N*-((3-(*tert*-butyl)-1*H*-pyrazol-4-yl)methyl)piperidin-4-amine **36.HCl** (1.23 g, 3.54 mmol) to afford *N*-((3-(*tert*-butyl)-1*H*-pyrazol-4-yl)methyl)piperidin-4-amine **36** (0.83 g, 3.49 mmol, 98 %). **^{1}H NMR** (500 MHz, MeOD) δ 7.5 (s, 1H), 3.82 (s, 2H), 3.13 - 3.06 (m, 2H), 2.73 - 2.68 (m, 1H), 2.66 - 2.59 (m, 2H), 1.98 - 1.94 (m, 2H), 1.41 - 1.39 (m, 11H). **LCMS** (ES+): m/z 237 $[M+H]^+$, 95 %.

***N*-((3-(*tert*-butyl)-1*H*-pyrazol-4-yl)methyl)-1-(pyridin-2-ylmethyl)piperidin-4-amine, (1)**

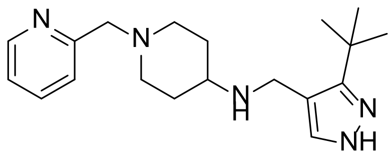

1 was prepared according to general procedure 1 from **34.HCl** (449 mg, 1.97 mmol, 1.0 equiv. HCl) and 3-*tert*-butyl-1*H*-pyrazole-4-carbaldehyde (300 mg, 1.97 mmol, 1 equiv.) in DCM (0.3 M) using 1.5 equiv. of NaOAc and 2 equiv. of $NaBH_3CN$ to afford desired compound (230 mg, 0.69 mmol, 35 %) as a white gum. **^{1}H NMR** (500 MHz, MeOD) δ 8.54 – 8.51 (m, 1H), 7.88 – 7.84 (m, 1H), 7.7 (br s, 1H), 7.55 (d, *J* = 7.8 Hz, 1H), 7.38 –z 7.34 (m, 1H), 4.29 (s, 2H), 3.75 (s, 2H), 3.30 – 3.22 (m, 1H), 3.10 – 3.04 (m, 2H), 2.34 – 2.26 (m, 2H), 2.22 – 2.16 (m, 2H), 1.83 – 1.73 (m, 2H), 1.43 (s, 9H). **^{13}C NMR** (126 MHz, MeOD) δ 157.4, 148.4, 137.4, 123.8, 122.7, 107.7, 62.6, 55.4, 51.3, 39.7,

32.0, 29.3, 28.3. **LCMS** 2.04 min, 100 %, *m/z* 328.3 [M+H]$^+$. **HRMS** (ES+): calcd. for $C_{19}H_{29}N_5$ [M+H]$^+$ 328.2496, found 328.2505 (2.7 ppm).

(5-(*tert*-butyl)-1*H*-pyrazol-3-yl)methanol (intermediate 1, for synthesis of compound 2)

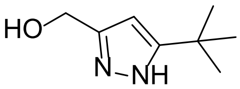

To a solution of ethyl 5-(*tert*-butyl)-1*H*-pyrazole-3-carboxylate (500 mg, 2.6 mmol, 1.0 equiv.) in THF (0.3 M) was added $LiAlH_4$ (290 mg, 7.64 mmol, 2.9 equiv.) at 0 °C and the mixture was stirred at room temperature for 2.5 h. The reaction quenched with 15 % NaOH solution slowly at 0 °C and the solution extracted with EtOAc. The combined organics were dried over Na_2SO_4, filtered and concentrated *in vacuo* to afford (5-(*tert*-butyl)-1*H*-pyrazol-3-yl)methanol (0.4 g, crude) as a white solid.

5-(*tert*-butyl)-1*H*-pyrazole-3-carbaldehyde (intermediate 2, for synthesis of compound 2)

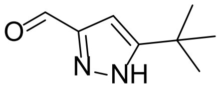

A solution of (5-(*tert*-butyl)-1*H*-pyrazol-3-yl)methanol (400 mg, 2.6 mmol, 1.0 equiv.) and MnO_2 (2.26 g, 25.9 mmol, 10 equiv.) in THF (0.43 M) was stirred at room temperature for 1 h. The reaction mixture was filtered and concentrated under vacuum to afford 5-(*tert*-butyl)-1*H*-pyrazole-3-carbaldehyde (378 mg, crude) as a yellow oil. **LCMS** (ES+): *m/z* 153.2 [M+H]$^+$, 100 %.

***N*-((5-(*tert*-butyl)-1*H*-pyrazol-3-yl)methyl)-1-(pyridin-2-ylmethyl)piperidin-4-amine, (2)**

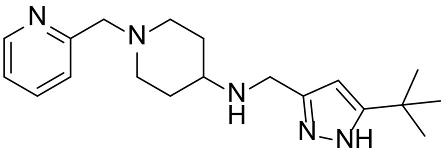

2 was prepared according to general procedure 1 from **34** (570 mg, 2.50 mmol, 1.0 equiv. HCl) and 5-(*tert*-butyl)-1*H*-pyrazole-3-carbaldehyde (343 mg, 2.25 mmol, 0.9 equiv.) in EtOH (0.5 M) to afford desired compound (141 mg, 0.41 mmol, 17 %) as a colorless gum. **^{1}H NMR** (500 MHz, MeOD) δ 8.48 (d, *J* = 4.2 Hz, 1H), 7.85 - 7.81 (m, 1H), 7.57 - 7.54 (m, 1H), 7.34 - 7.30 (m, 1H), 6.12 (s, 1H), 3.78 (s, 2H), 3.66 - 3.65 (m, 2H), 2.91 (d, *J* = 12.1 Hz, 2H), 2.59 - 2.50 (m, 1H), 2.17 - 2.10 (m, 2H), 1.96 - 1.90 (m, 2H), 1.54 - 1.44 (m, 2H), 1.32 (s, 9H). **^{13}C NMR** (126 MHz, MeOD) δ 158.1, 148.1, 137.3, 123.8, 122.4, 99.9, 63.4, 53.8, 52.2, 31.2, 29.3. **LCMS** 2.22, 100 %, *m/z* 328.2 [M+H]$^+$. **HRMS** (ES+): calcd. for $C_{19}H_{29}N_5$ [M+H]$^+$ 328.2496, found 328.2496 (0.0 ppm).

3-(*tert*-butyl)-1-methyl-1*H*-pyrazole-4-carbaldehyde (Intermediate 1, for synthesis of compound 3)

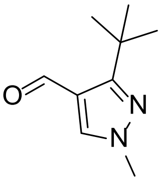

A mixture of 3-(*tert*-butyl)-1*H*-pyrazole-4-carbaldehyde (300 mg, 1.97 mmol, 1.0 equiv.), CH_3I (560 mg, 3.94 mmol, 245 µL, 2.0 equiv.) and K_2CO_3 (1.09 g, 7.88 mmol, 4.0 equiv.) in DMF (0.5 M) was stirred at 85 °C for 1.5 h, quenched with saturated NH_4Cl solution, extracted with EtOAc and the combined organics dried over Na_2SO_4, filtered and concentrated *in vacuo* and the residue

was purified by reverse phase flash column chromatography (5-95 % CH_3CN in water (+0.1 % formic acid)) to afford 3-(*tert*-butyl)-1-methyl-1*H*-pyrazole-4-carbaldehyde (255 mg, 1.46 mmol, 74 %) as yellow oil. **^{1}H NMR** (400 MHz, $CDCl_3$) δ 10.00 (s, 1H), 7.87 (s, 1H), 3.87 (s, 3H), 1.41 (s, 9H). **LCMS** (ES+): *m/z* 167.2 $[M+H]^+$, 100 %.

***N*-((3-(*tert*-butyl)-1-methyl-1*H*-pyrazol-4-yl)methyl)-1-(pyridin-2-ylmethyl)piperidin-4-amine (3)**

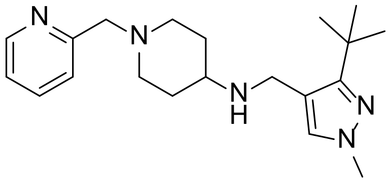

3 was prepared according to general procedure 1 from **34.HCl** (300 mg, 1.01 mmol, 1.0 equiv. HCl) and 3-(*tert*-butyl)-1-methyl-1*H*-pyrazole-4-carbaldehyde (197 mg, 1.19 mmol, 1.2 equiv.) in EtOH (0.3 M) to afford desired compound (88 mg, 0.24 mmol, 19 %) as a yellow gum. **^{1}H NMR** (500 MHz, MeOD) δ 8.50 - 8.47 (m, 1H), 7.86 - 7.82 (m, 1H), 7.56 (d, *J* = 7.9 Hz, 1H), 7.47 (s, 1H), 7.35 - 7.31 (m, 1H), 3.79 - 3.78 (m, 5H), 3.67 - 3.66 (m, 2H), 2.92 (d, *J* = 11.9 Hz, 2H), 2.64 - 2.55 (m, 1H), 2.21 - 2.15 (m, 2H), 1.97 - 1.91 (m, 2H), 1.56 - 1.46 (m, 2H), 1.35 - 1.34 (m, 9H). **^{13}C NMR** (126 MHz, MeOD) δ 158.1, 157.1, 148.2, 137.3, 131.6, 124.8, 122.5, 115.9, 63.5, 54.4, 52.2, 41.1, 37.6, 32.6, 31.5, 29.3. **LCMS** 2.71, 100 %, *m/z* 342.4 $[M+H]^+$. **HRMS** (ES+): calcd. for $C_{20}H_{31}N_5$ $[M+H]^+$ 342.2652, found 342.2653 (0.3 ppm).

***N*-((3-methyl-1*H*-pyrazol-4-yl)methyl)-1-(pyridin-2-ylmethyl)piperidin-4-amine (4)**

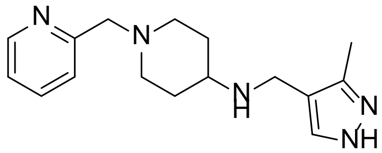

4 was prepared according to general procedure 3 from **34.HCl** (300 mg, 1.32 mmol, 1.0 equiv. HCl) and 3-methyl-1*H*-pyrazole-4-carbaldehyde (145 mg, 1.32 mmol, 1 equiv.) in EtOH (0.2 M) using 1.5 equiv. of AcOH in addition to NaOAc (0.1 equiv.), the reaction mixture extracted with DCM, the combined organics concentrated *in vacuo* and purified by preparative TLC (SiO_2, 10:1 DCM/MeOH, R_f = 0.15) and preparative HPLC (6-36 % CH_3CN in water (+1 % NH_4HCO_3)) to afford desired compound (16 mg, 0.05 mmol, 4 %) as a colorless gum. **^{1}H NMR** (500 MHz, MeOD) δ 8.50 – 8.47 (m, 1H), 7.85 – 7.80 (m, 1H), 7.56 (d, *J* = 7.4 Hz, 1H), 7.51 (s, 1H), 7.34 – 7.30 (m, 1H), 3.67 – 3.63 (m, 4H), 2.95 – 2.89 (m, 2H), 2.59 – 2.51 (m, 1H), 2.27 (s, 3H), 2.19 – 2.10 (m, 2H), 1.98 – 1.90 (m, 2H), 1.55 – 1.44 (m, 2H). **^{13}C NMR** (126 MHz, MeOD) δ 158.0, 148.1, 137.2, 123.8, 122.5, 115.5, 63.5, 53.9, 52.3, 38.8, 31.3, 8.7. **LCMS** 2.19, 99 %, *m/z* 286.2 $[M+H]^+$. **HRMS** (ES+): calcd. for $C_{16}H_{24}N_5$ $[M+H]^+$ 286.2026, found 286.2024 (0.6 ppm).

(3-isopropyl-1*H*-pyrazol-4-yl)methanol (Intermediate 1, for synthesis of compound 5)

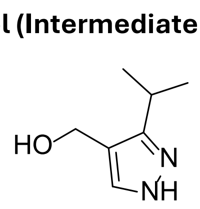

To a solution of ethyl 3-isopropyl-1*H*-pyrazole-4-carboxylate (500 mg, 2.74 mmol, 1.0 *eq*uiv.) in THF (0.14 M) was added $LiAlH_4$ (416 mg, 10.9 mmol, 4.0 *eq*uiv.) at 0 °C. The reaction mixture warmed to room temperature then heated at 60 °C, after 2 h cooled to 0 °C, brine added dropwise and the reaction mixture filtered. The filtrate was lyophilized to give a residue which was purified

by normal phase flash chromatography eluting with 2:1 Pet ether:EtOAc to 10:1 DCM:MeOH to afford (3-isopropyl-1*H*-pyrazol-4-yl)methanol (260 mg, 1.84 mmol, 67 %) as a white solid. **^{1}H NMR** (400 MHz, MeOD) δ 7.66 - 7.20 (m, 1H), 4.50 (br s, 2H), 3.25 - 3.05 (m, 1H), 1.30 (br d, *J* = 7.2 Hz, 6H). **LCMS** (ES+): *m/z* (%) 141.2 $[M+H]^+$.

3-isopropyl-1*H*-pyrazole-4-carbaldehyde (Intermediate 2, for synthesis of compound 5)

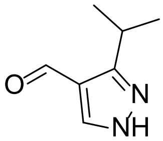

To a solution of (3-isopropyl-1*H*-pyrazol-4-yl)methanol (200 mg, 1.43 mmol, 1.0 *eq*uiv.) in THF (0.04 M) was added Dess-Martin (907 mg, 2.14 mmol, 1.5 *eq*uiv.). The reaction mixture stirred at 25 °C for 1 h, filtered and concentrated *in vacuo,* saturated $NaHCO_3$ solution and saturated Na_2SO_3 solution was added to the residue and the aqueous layer extracted with 10:1 DCM:MeOH. The combined organic layers were washed with brine, dried over anhydrous Na_2SO_4, filtered and concentrated *in vacuo* to afford 3-isopropyl-1*H*-pyrazole-4-carbaldehyde (120 mg, 0.87 mmol, 61 %) as yellow oil. **^{1}H NMR** (400 MHz, MeOD) δ 9.89 (s, 1H), 8.02 (br s, 1H), 3.54 (br s, 1H), 1.33 (br d, *J* = 6.8 Hz, 6H). **LCMS** (ES+): *m/z* 139.2 $[M+H]^+$, 98 %.

***N*-((3-isopropyl-1*H*-pyrazol-4-yl)methyl)-1-(pyridin-2-ylmethyl)piperidin-4-amine (5)**

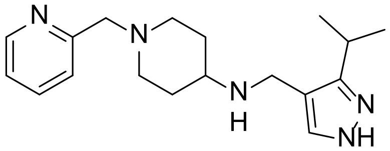

5 was prepared according to general procedure 3 from **34.HCl** (217 mg, 0.96 mmol, 1.2 *eq*uiv. HCl) and 3-isopropyl-1*H*-pyrazole-4-carbaldehyde (110 mg, 0.79 mmol, 1.0 *eq*uiv.) in MeOH (0.16 M) using 1 equiv. of AcOH in addition to 2 equiv. of AcONa and the product extracted with 10:1 DCM:MeOH, the organic layers were concentrated *in vacuo* and the residue purified by preparative HPLC (6-36 % CH_3CN in water (+1 % NH_4HCO_3) using Phenomenex Gemini-NX C18 75 * 30 mm * 3 µm column) to afford desired compound (34.3 mg, 0.11 mmol, 13 %) as a yellow gum**.** **^{1}H NMR** (500 MHz, MeOD) δ 8.50 – 8.47 (m, 1H), 7.86 – 7.81 (m, 1H), 7.56 (d, *J* = 7.8 Hz, 1H), 7.51 (s, 1H), 7.35 – 7.30 (m, 1H), 3.68 (s, 2H), 3.66 (s, 2H), 3.15 – 3.06 (m, 1H), 2.95 – 2.88 (m, 2H), 2.60 – 2.52 (m, 1H), 2.19 – 2.11 (m, 2H), 1.97 – 1.90 (m, 2H), 1.56 – 1.45 (m, 2H), 1.30 (s, 3H), 1.29 (s, 3H). **^{13}C NMR** (126 MHz, MeOD) δ 158.0, 148.1, 137.3, 123.8, 122.5, 63.4, 53.9, 52.3, 38.9, 31.3, 21.4. **LCMS** 2.38, 100 %, *m/z* 314.3 $[M+H]^+$. **HRMS** (ES+): calcd. for $C_{18}H_{28}N_5$ $[M+H]^+$ 314.2339, found 314.2347 (2.5 ppm).

***N*-((3-(tert-butyl)-1*H*-pyrazol-4-yl)methyl)-1-(pyridin-2-ylmethyl)piperidin-4-amine (6)**

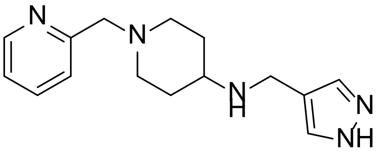

6 was prepared according to general procedure 2 from **34** (100 mg, 0.52 mmol, 1 equiv.) and 1*H*-pyrazole-4-carbaldehyde (50 mg, 0.52 mmol, 1 equiv.) in DCM (0.5 M) to afford the desired compound (60 mg, 0.21 mmol, 40 %) as a clear oil. **^{1}H NMR** (500 MHz, MeOD) δ 8.50 – 8.47 (m, 1H), 7.85 – 7.80 (m, 1H), 7.62 – 7.52 (m, 3H), 7.35 – 7.30 (m, 1H), 3.73 (s, 2H), 3.65 (s, 2H), 2.94 – 2.88 (m, 2H), 2.58 – 2.50 (m, 1H), 2.17 – 2.10 (m, 2H), 1.96 – 1.89 (m, 2H), 1.54 – 1.44 (m, 2H). **^{13}C NMR** (126 MHz, MeOD) δ 158.0, 148.1, 137.3, 123.8, 122.5, 118.4, 63.4, 53.6, 52.3, 39.4, 31.2.

LCMS 1.09, 100 %, *m/z* 272.1 [M+H]$^+$. **HRMS** (ES+): calcd. for $C_{15}H_{22}N_5$ [M+H]$^+$ 272.1870, found 272.1870 (0.2 ppm).

***N*-((3-cyclopropyl-1*H*-pyrazol-4-yl)methyl)-1-(pyridin-2-ylmethyl)piperidin-4-amine (7)**

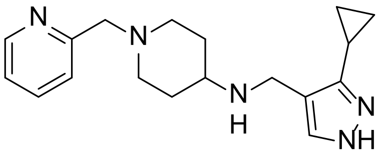

7 was prepared according to general procedure 2 from **34** (95 mg, 0.49 mmol, 1 equiv.) and 3-cyclopropyl-1*H*-pyrazole-4-carbaldehyde (68 mg, 0.49 mmol, 1 equiv.) in DCM (0.25 M) to afford the desired compound (90 mg, 0.27 mmol, 55 %) as a glassy solid. **^{1}H NMR** (500 MHz, MeOD) δ 8.50 – 8.47 (m, 1H), 7.86 – 7.81 (m, 1H), 7.56 (d, *J* = 7.8 Hz, 1H), 7.50 (s, 1H), 7.35 – 7.30 (m, 1H), 3.75 (s, 2H), 3.66 (s, 2H), 2.95 – 2.88 (m, 2H), 2.60 – 2.52 (m, 1H), 2.19 – 2.11 (m, 2H), 1.98 – 1.92 (m, 2H), 1.92 – 1.86 (m, 1H), 1.56 – 1.45 (m, 2H), 0.97 – 0.90 (m, 2H), 0.82 – 0.76 (m, 2H). **^{13}C NMR** (126 MHz, MeOD) δ 158.0, 148.1, 137.2, 123.8, 122.5, 116.4, 63.5, 52.3, 38.8, 31.3, 5.7. **LCMS** 1.31, 100 %, *m/z* 312.2 [M+H]$^+$. **HRMS** (ES+): calcd. for $C_{18}H_{26}N_5$ [M+H]$^+$ 312.2183, found 312.2187 (1.5 ppm).

(3-(trifluoromethyl)-1*H*-pyrazol-4-yl)methanol (Intermediate 1, for synthesis of compound 8)

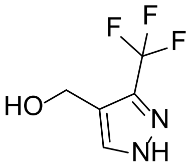

To a solution of ethyl 5-(trifluoromethyl)-1*H*-pyrazole-4-carboxylate (300 mg, 1.44 mmol, 1 equiv.) in THF (0.18 M) cooled to 0 ºC under N_2 was added 1M $LiAlH_4$ (3.6 mL, 3.60 mmol, 2.5 equiv.) and the reaction stirred for 2 h, quenched by the dropwise addition of water while cooling in an ice bath, the mixture extracted with EtOAc and the solution filtered to remove precipitate and the organics were separated, dried over Na_2SO_4, filtered and concentrated *in vacuo* to yield (3-(trifluoromethyl)-1*H*-pyrazol-4-yl)methanol (173 mg, 0.83 mmol, 58 %). **^{1}H NMR** (500MHz, MeOD) δ 7.77 (s, 1H), 4.63 (s, 2H).

3-(trifluoromethyl)-1*H*-pyrazole-4-carbaldehyde (Intermediate 2, for synthesis of compound 8)

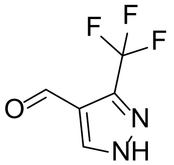

To a suspension of [5-(trifluoromethyl)-1*H*-pyrazol-4-yl]methanol (173 mg, 0.83 mmol, 1 equiv.) in DCM (0.17 M) cooled to 0 ºC was added Dess-martin periodinane (441 mg, 1.04 mmol, 1.25 equiv.) and the reaction slowly warmed to room temperature and stirred for 16 h, diluted with DCM, filtered and concentrated *in vacuo* and purified by normal phase flash chromatography eluting with EtOAc/heptane gradient (0 to 100 %) afford 3-(trifluoromethyl)-1*H*-pyrazole-4-carbaldehyde (54 mg, 0.32 mmol, 39 %). **^{1}H NMR** (500 MHz, MeOD) δ 9.95 (s, 1H), 8.43 (s, 1H).

1-(pyridin-2-ylmethyl)-*N*-((3-(trifluoromethyl)-1*H*-pyrazol-4-yl)methyl)piperidin-4-amine (8)

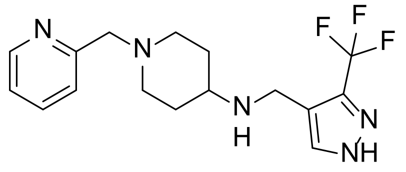

8 was prepared according to general procedure 2 from **34** (63 mg, 0.33 mmol, 1 equiv.) and 3-(trifluoromethyl)-1*H*-pyrazole-4-carbaldehyde (54 mg, 0.33 mmol, 1 equiv.) in DCM (0.17 M) to afford the desired compound (24 mg, 0.067 mmol, 20 %) as red sticky gum. **^{1}H NMR** (500 MHz, MeOD) δ 8.50 – 8.47 (m, 1H), 7.86 – 7.82 (m, 1H), 7.81 (s, 1H), 7.56 (d, *J* = 7.8 Hz, 1H), 7.35 – 7.30 (m, 1H), 3.81 (s, 2H), 3.66 (s, 2H), 2.95 – 2.88 (m, 2H), 2.59 – 2.51 (m, 1H), 2.19 – 2.11 (m, 2H), 1.96 – 1.89 (m, 2H), 1.55 – 1.44 (m, 2H). **^{13}C NMR** (126 MHz, MeOD) δ 157.9, 148.1, 137.2, 129.9, 123.8, 123.3, 122.5, 121.1, 117.7, 63.4, 53.9, 52.2, 38.5, 31.3. **LCMS** 2.08, 100 %, *m/z* 340.3 $[M+H]^+$. **HRMS** (ES+): calcd. for $C_{16}H_{21}F_3N_5$ $[M+H]^+$ 340.1744, found 340.1752 (2.5 ppm).

***N*-((1-(*tert*-butyl)-1*H*-pyrazol-5-yl)methyl)-1-(pyridin-2-ylmethyl)piperidin-4-amine (9)**

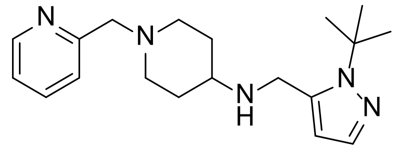

9 was prepared according to general procedure 2 from **34**(40 mg, 0.21 mmol, 1.0 equiv.) and 1-*tert*-butyl-1*H*-pyrazole-5-carbaldehyde (32 mg, 0.21 mmol, 1.0 equiv.) in DCM (0.2 M) to afford the desired compound (25 mg, 0.07 mmol, 35 %) as a gum**. ^{1}H NMR** (500 MHz, MeOD) δ 8.49 - 8.48 (m, 1H), 7.86 - 7.81 (m, 1H), 7.58 - 7.55 (m, 1H), 7.35 - 7.31 (m, 2H), 6.34 (d, *J* = 1.7 Hz, 1H), 4.03 (s, 2H), 3.67 (s, 2H), 2.95 - 2.90 (m, 2H), 2.65 - 2.58 (m, 1H), 2.21 - 2.15 (m, 2H), 1.98 - 1.92 (m, 2H), 1.65 (s, 9H), 1.57 - 1.48 (m, 2H). **^{13}C NMR** (126 MHz, MeOD) δ 157.9, 148.1, 141.7, 137.3, 136.2, 123.8, 122.5, 106.8, 63.5, 60.1, 54.4, 52.3, 43.0, 31.5, 29.2. **LCMS** 1.46, 100 %, *m/z* 328.3 $[M+H]^+$. **HRMS** (ES+): calcd. for $C_{19}H_{29}N_5$ $[M+H]^+$ 328.2496, found 328.2489 (2.1 ppm).

***N*-((1-(*tert*-butyl)-1*H*-imidazol-5-yl)methyl)-1-(pyridin-2-ylmethyl)piperidin-4-amine (10)**

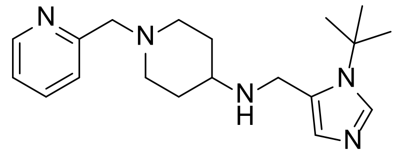

10 was prepared according to general procedure 2 from **34** (40 mg, 0.21 mmol, 1.0 equiv.) and 1-(*tert*-butyl)-1*H*-imidazole-5-carbaldehyde (32 mg, 0.21 mmol, 1.0 equiv.) in DCM (0.2 M) to afford the desired compound (28 mg, 0.08 mmol, 38 %) as a gum. **^{1}H NMR** (500 MHz, MeOD) δ 8.49 - 8.47 (m, 1H), 7.85 - 7.81 (m, 1H), 7.73 (d, *J* = 1.4 Hz, 1H), 7.58 - 7.55 (m, 1H), 7.34 - 7.31 (m, 1H), 7.22 - 7.21 (m, 1H), 3.72 (s, 2H), 3.66 - 3.65 (m, 2H), 2.94 - 2.89 (m, 2H), 2.59 - 2.51 (m, 1H), 2.18 - 2.11 (m, 2H), 1.95 - 1.90 (m, 2H), 1.59 (s, 9H), 1.54 - 1.46 (m, 2H). **^{13}C NMR** (126 MHz, MeOD) δ 158.1, 148.1, 138.9, 137.3, 134.0, 123.8, 122.5, 114.5, 63.5, 55.0, 53.8, 52.2, 42.9, 31.3, 29.3. **LCMS** 1.42, 100 %, *m/z* 328.3 $[M+H]^+$. **HRMS** (ES+): calcd. for $C_{19}H_{29}N_5$ $[M+H]^+$ 328.2496, found 328.2508 (3.8 ppm).

***N*-[(2-*tert*-butylphenyl)methyl]-1-(2-pyridylmethyl)piperidin-4-amine (11)**

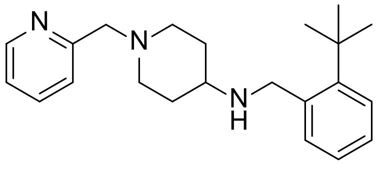

11 was prepared according to general procedure 2 from **34** (40 mg, 0.21 mmol, 1.0 equiv.) and 2-*tert*-butylbenzaldehyde (34 mg, 0.21 mmol, *1* equiv.) in DCM (0.2 M) to afford the desired compound (24 mg, 0.068 mmol, 32 %) as a gum. **^{1}H NMR** (500 MHz, MeOD) δ 8.50 - 8.48 (m, 1H), 7.86 - 7.82 (m, 1H), 7.57 (d, *J* = 7.8 Hz, 1H), 7.44 - 7.38 (m, 2H), 7.35 - 7.31 (m, 1H), 7.21 - 7.15 (m, 2H), 4.01 (s, 2H), 3.67 (s, 2H), 2.96 - 2.91 (m, 2H), 2.69 - 2.62 (m, 1H), 2.23 - 2.16 (m, 2H), 2.00 - 1.95 (m, 2H), 1.60 - 1.51 (m, 2H), 1.44 - 1.44 (m, 9H). **^{13}C NMR** (126 MHz, MeOD) δ 158.0, 148.1, 147.4, 138.2, 137.3, 130.9, 126.6, 125.8, 125.7, 123.8, 122.5, 63.5, 55.3, 52.3, 48.7, 35.2, 31.6, 31.0. **LCMS** 2.25, 96 %, *m/z* 338.3 [M+H]$^+$. **HRMS** (ES+): calcd. for $C_{22}H_{31}N_3$ [M+H]$^+$ 338.2591, found 338.2590 (0.2 ppm).

***tert*-butyl (3-((pyridin-2-ylmethyl)amino)propyl)carbamate (Intermediate 1, for synthesis of compound 12)**

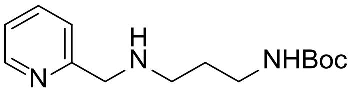

tert-Butyl (3-((pyridin-2-ylmethyl)amino)propyl)carbamate was prepared according to general procedure 3 from *tert*-butyl (3-aminopropyl)carbamate (976 mg, 5.60 mmol, 978 µL, 1.2 equiv.) and picolinaldehyde, (500 mg, 4.67 mmol, 1.0 equiv.) in EtOH (0.47 M). The reaction mixture was quenched with water, extracted with DCM, the combined organics concentrated *in vacuo* and purified by flash chromatography eluting with 5:1 pet ether:EtOAc to 3:1 EtOAc:MeOH followed by reverse phase flash column chromatography (5-95 % CH_3CN in water (+0.1 % FA)) to afford *tert*-butyl (3-((pyridin-2-ylmethyl)amino)propyl)carbamate (450 mg, 1.7 mmol, 36 %) as a yellow oil. **^{1}H NMR** (400 MHz, $CDCl_3$) δ 8.56 (d, *J* = 4.4 Hz, 1H), 7.70 - 7.62 (m, 1H), 7.33 (d, *J* = 8.0 Hz, 1H), 7.21 - 7.15 (m, 1H), 5.21 (br s, 1H), 3.95 (s, 2H), 3.30-3.19 (m, 2H), 2.76 (t, *J* = 6.4 Hz, 2H), 1.81 - 1.68 (m, 2H), 1.44 (s, 9H). MS m/z 266.3 [M+H]$^+$. **LCMS** (ES+): *m/z* 266.3 [M+H]$^+$, 99 %.

***N*^{1}-(pyridin-2-ylmethyl)propane-1,3-diamine HCl (Intermediate 2, for synthesis of compound 12)**

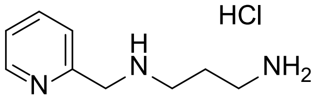

***N*^{1}-(pyridin-2-ylmethyl)propane-1,3-diamine** was prepared according to general procedure 5 from *tert*-butyl (3-((pyridin-2-ylmethyl)amino)propyl)carbamate (400 mg, 1.51 mmol, 1.0 equiv.) in CH_3CN (0.76 M) to afford *N*^{1}-(pyridin-2-ylmethyl)propane-1,3-diamine (300 mg, crude, HCl) as a yellow solid that was used without further purification.

***N*^{1}-((3-(*tert*-butyl)-1*H*-pyrazol-4-yl)methyl)-*N*^{3}-(pyridin-2-ylmethyl)propane-1,3-diamine (12)**

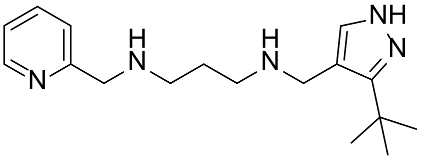

1 was prepared according to general procedure 3 from N^1-(pyridin-2-ylmethyl)propane-1,3-diamine HCl (300 mg, 1.49 mmol, 1.3 equiv.) and 3-*tert*-butyl-1*H*-pyrazole-4-carbaldehyde (174 mg, 1.14 mmol, 1.0 equiv.) using EtOH (0.2 M) with the addition of NaOAc (1.5 equiv.). The reaction mixture was quenched with water, concentrated *in vacuo* and purified by flash chromatography eluting with EtOAc :MeOH (20 to 35 %) and preparative HPLC (10-40 % CH_3CN in water (+1 % NH_4HCO_3)) to afford product which was further purified by preparative HPLC (0-18 % CH_3CN in water (+0.05 % HCl) using 3_Phenomenex Luna C18 75 * 30 mm * 3 μm column) to afford the desired compound (33 mg, 0.10 mmol, 9 %) as a white solid. **^{1}H NMR** (500 MHz, MeOD) δ 8.90 - 8.87 (m, 1H), 8.54 (s, 1H), 8.46 - 8.42 (m, 1H), 8.11 (d, *J* = 7.9 Hz, 1H), 7.93 - 7.89 (m, 1H), 4.68 (s, 2H), 4.49 (s, 2H), 3.47 - 3.39 (m, 4H), 2.45 - 2.36 (m, 2H), 1.53 - 1.52 (m, 9H). **^{13}C NMR** (126 MHz, MeOD) δ 154.6, 147.6, 145.4, 143.5, 136.1, 126.5, 126.2, 110.4, 48.4, 45.0, 44.9, 41.5, 32.7, 28.6, 22.7. **LCMS** 2.12, 100 %, *m/z* 302.3 $[M+H]^+$. **HRMS** (ES+): calcd. for $C_{17}H_{27}N_5$ $[M+H]^+$ 302.2339, found 302.2338 (0.3 ppm).

***Tert*-butyl (2-((pyridin-2-ylmethyl)amino)ethyl)carbamate (Intermediate 1, for synthesis of compound 13)**

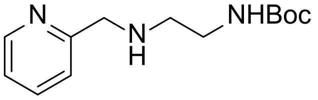

Tert-butyl (2-((pyridin-2-ylmethyl)amino)ethyl)carbamate was prepared according to general procedure 3 using *tert*-butyl 2-aminoethyl)carbamate (897 mg, 5.60 mmol, 880 μL, 1.2 equiv.) and picolinaldehyde (500 mg, 4.67 mmol, 1.0 equiv.) in EtOH (0.47 M).(450 mg, 1.76 mmol, 38 %) isolated as a yellow oil. **^{1}H NMR** (400 MHz, $CDCl_3$) δ 8.57 (d, *J* = 4.8 Hz, 1H), 7.70 - 7.63 (m, 1H), 7.30 (d, *J* = 7.6 Hz, 1H), 7.22 - 7.16 (m, 1H), 5.18 (br s, 1H), 3.96 (s, 2H), 3.35 - 3.25 (m, 2H), 2.85 - 2.82 (m, 2H), 1.45 (s, 9H). **LCMS** (ES+): *m/z* 252.3 $[M+H]^+$, 100 %.

N^1-(pyridin-2-ylmethyl)ethane-1,2-diamine hydrochloride (Intermediate 2, for synthesis of compound 13)

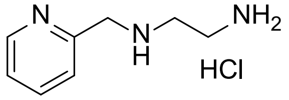

N^1-(pyridin-2-ylmethyl)ethane-1,2-diamine hydrochloride was prepared following the same procedure as intermediate 2, compound 12 (290 mg, crude) isolated as a yellow solid and used without further purification.

N^1-((3-(*tert*-butyl)-1*H*-pyrazol-4-yl)methyl)-N^2-(pyridin-2-ylmethyl)ethane-1,2-diamine (13)

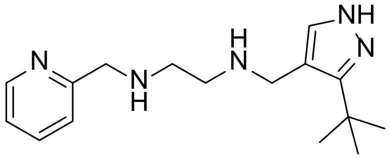

13 was prepared following the same procedure as **12** using intermediate 2, compound 13.The residue was purified by preparative HPLC (10-40 % CH_3CN in water (+1 % NH_4HCO_3) to afford product which was further purified by preparative HPLC (0-15 % CH_3CN in water (+0.05 % HCl) using Phenomenex Synergi C18 150 * 25 mm * 10 μm column) followed by additional preparative HPLC (0-5 % CH_3CN in water (+0.05 % HCl) using 3_Phenomenex Luna C18 75 * 30 mm * 3 μm column) to afford desired compound (25 mg, 0.08 mmol, 7 %) as a yellow solid. **^{1}H NMR** (500 MHz, MeOD) δ 8.87 (d, *J* = 4.1 Hz, 1H), 8.56 (s, 1H), 8.42 (t, *J* = 7.2 Hz, 1H), 8.13 (d, *J* = 7.5 Hz, 1H), 7.93 - 7.87 (m, 1H), 4.75 (s, 2H), 4.57 (s, 2H), 4.56 (s, 2H), 3.80 (s, 4H), 1.53 (s, 9H). **^{13}C NMR** (126 MHz, MeOD) δ 154.4, 147.6, 145.4, 143.5, 136.2, 126.4, 126.2, 110.1, 48.6, 43.8, 41.8, 32.7, 28.7.

LCMS 1.96, 100 %, *m/z* 288.3 [M+H]$^+$. **HRMS** (ES+): calcd. for $C_{16}H_{25}N_5$ [M+H]$^+$ 288.2183, found 288.2180 (0.8 ppm).

***tert*-butyl (*R*)-3-((((3-(*tert*-butyl)-1*H*-pyrazol-4-yl)methyl)amino)methyl) pyrrolidine-1-carboxylate (Intermediate 1, for synthesis of compound 14)**

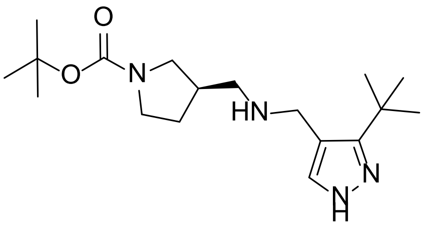

tert-butyl (*R*)-3-((((3-(*tert*-butyl)-1*H*-pyrazol-4-yl)methyl)amino)methyl) pyrrolidine-1-carboxylate was prepared according to general procedure 3 from *tert*-butyl (3*R*)-3-(aminomethyl)pyrrolidine-1-carboxylate (500 mg, 2.50 mmol, 1.0 *eq*uiv.) and 3-*tert*-butyl-1*H*-pyrazole-4-carbaldehyde (303 mg, 2.00 mmol, 0.8 *eq*uiv.) in MeOH (0.25 M) using 1 equiv. of AcOH. The crude residue was purified by flash chromatography eluting with 2:1 Pet. Ether:EtOAc to 20:1 EtOAc:MeOH to afford *tert*-butyl (3*R*)-3-[[(3-*tert*-butyl-1*H*-pyrazol-4-yl)methylamino]methyl]pyrrolidine-1-carboxylate (390 mg, 1.07 mmol, 43 %) as a yellow solid. **^{1}H NMR** (400 MHz, $CDCl_3$) δ 7.49 (s, 1H), 3.80 (br s, 2H), 3.58 - 3.42 (m, 2H), 3.36 - 3.22 (m, 1H), 3.08 - 2.96 (m, 1H), 2.79 - 2.61 (m, 2H), 2.45 - 2.28 (m, 1H), 2.01 (br s, 1H), 1.64 - 1.55 (m, 1H), 1.46 (s, 9H), 1.39 (s, 9H). **LCMS** (ES+): *m/z* 337.2 [M+H]$^+$, 100 %.

(*S*)-1-(3-(*tert*-butyl)-1*H*-pyrazol-4-yl)-*N*-(pyrrolidin-3-ylmethyl) methanamine (Intermediate 2, compound 14)

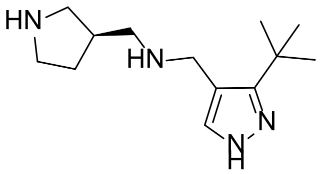

(*S*)-1-(3-(*tert*-butyl)-1*H*-pyrazol-4-yl)-*N*-(pyrrolidin-3-ylmethyl) methanamine was prepared according to general procedure 4 from *tert*-butyl (3*R*)-3-[[(3-*tert*-butyl-1*H*-pyrazol-4-yl)methylamino]methyl]pyrrolidine-1-carboxylate (370 mg, 1.10 mmol, 1.0 equiv.) in CH_3CN (0.28 M) to afford the desired compound as a white solid. (400 mg, crude, HCl) **^{1}H NMR** (400 MHz, MeOD) δ 8.54 (s, 1H), 4.47 (s, 2H), 3.65 - 3.60 (m, 1H), 3.52 - 3.45 (m, 1H), 3.40 (br d, *J* = 7.2 Hz, 2H), 3.40 - 3.30 (m, 1H), 3.23 - 3.14 (m, 1H), 2.93 (td, *J* = 8.0, 16.0 Hz, 1H), 2.41 (s, 2H), 2.41 - 2.34 (m, 1H), 1.97 - 1.86 (m, 1H), 1.50 (s, 9H). **LCMS** (ES+): *m/z* 237.2 [M+H]$^+$, 100 %.

(*R*)-1-(3-(*tert*-butyl)-1*H*-pyrazol-4-yl)-*N*-((1-(pyridin-2-ylmethyl)pyrrolidin-3-yl)methyl)methanamine (14)

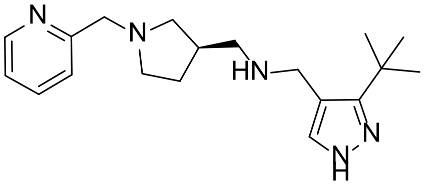

14 was prepared according to general procedure 3 from 1-(3-*tert*-butyl-1*H*-pyrazol-4-yl)-*N*-[[(3*R*)-pyrrolidin-3-yl]methyl]methanamine HCl (350 mg, 1.28 mmol, 1.0 *eq*uiv. HCl) and pyridine-2-carbaldehyde (109 mg, 1.03 mmol, 0.8 *eq*uiv.) in MeOH (0.1 M) using 1 equiv. of AcOH in addition to NaOAc (2 equiv.) and extracted with 10:1 DCM:MeOH. The crude was purified by preparative

HPLC (21-51 % CH_3CN in water (+1 % NH_4HCO_3)) to afford desired compound **14** (49.09 mg, 0.15 mmol, 11 %, 99.9 %ee) as a yellow gum. **^{1}H NMR** (500 MHz, MeOD) δ 8.48 – 8.51 (m, 1H), 7.81 – 7.86 (m, 1H), 7.49 – 7.53 (m, 2H), 7.31 – 7.36 (m, 1H), 3.73 – 3.83 (m, 4H), 2.83 – 2.88 (m, 1H), 2.60 – 2.72 (m, 4H), 2.40 – 2.47 (m, 1H), 2.33 – 2.38 (m, 1H), 2.02 – 2.11 (m, 1H), 1.50 – 1.57 (m, 1H), 1.38 (s, 9H). **^{13}C NMR** (126 MHz, MeOD) δ 158.15, 148.17, 137.30, 123.60, 122.48, 61.02, 58.44, 53.94, 53.49, 43.92, 37.17, 29.12, 28.69. **LCMS** 2.54, 97 %, *m/z* 328.2 $[M+H]^+$. **HRMS** (ES+): calcd. for $C_{19}H_{29}N_5$ $[M+H]^+$ 328.2501, found 328.2507 (1.8 ppm).

***tert*-butyl (3*S*)-3-[[(3-*tert*-butyl-1*H*-pyrazol-4-yl)methylamino]methyl]pyrrolidine-1-carboxylate** (**Intermediate 1, for synthesis of compound 15**)

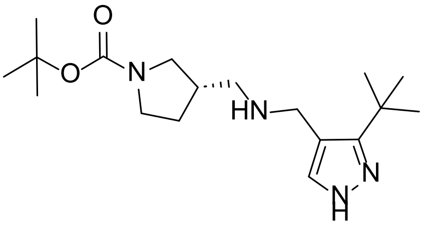

15 was prepared following the same procedure as **14** from *tert*-butyl (3*S*)-3-(aminomethyl)pyrrolidine-1-carboxylate (500 mg, 2.50 mmol, 1.0 *equiv.) and* 3-*tert*-butyl-1*H*-pyrazole-4-carbaldehyde (303 mg, 2.00 mmol, 0.8 *eq*uiv.).

(*S*)-1-(3-(*tert*-butyl)-1*H*-pyrazol-4-yl)-*N*-((1-(pyridin-2-ylmethyl)pyrrolidin-3-yl)methyl)methanamine (intermediate 2, compound 15)

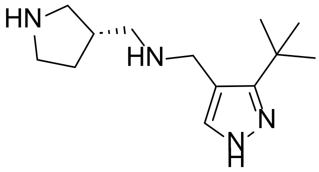

(380 mg, crude, HCl) isolated as a white solid. **^{1}H NMR** (400 MHz, MeOD) δ 8.62 (s, 1H), 4.49 (s, 2H), 3.66 - 3.60 (m, 1H), 3.52 - 3.45 (m, 1H), 3.41 (br d, *J* = 7.2 Hz, 2H), 3.38 - 3.35 (m, 1H), 3.21 (dd, *J* = 8.4, 11.6 Hz, 1H), 2.95 (td, *J* = 7.6, 15.2 Hz, 1H), 2.42 - 2.35 (m, 1H), 1.98 - 1.90 (m, 1H), 1.51 (s, 9H). **LCMS** (ES+): *m/z* 237.2 $[M+H]^+$, 100 %.

(*S*)-1-(3-(*tert*-butyl)-1*H*-pyrazol-4-yl)-*N*-((1-(pyridin-2-ylmethyl)pyrrolidin-3-yl)methyl)methanamine (15)

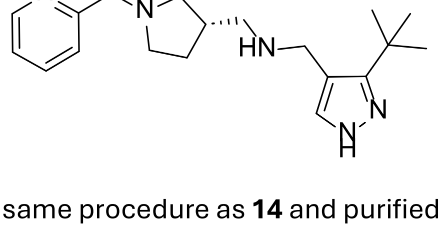

15 was prepared following the same procedure as **14** and purified by preparative HPLC (21-51 % CH_3CN in water (+1 % NH_4HCO_3)) to afford product which was further purified by preparative HPLC (23-53 % CH_3CN in water (+0.05 % NH_4OH) to afford compound **15** (33 mg, 0.101 mmol, 8 %, 99.9 %ee) as a yellow gum. **^{1}H NMR** (400 MHz, MeOD) δ 8.47 (td, *J* = 0.8, 4.0 Hz, 1H), 7.82 (dt, *J* = 2.0, 8.0 Hz, 1H), 7.50 (br d, *J* = 7.6 Hz, 2H), 7.33 - 7.30 (m, 1H), 3.84 - 3.68 (m, 4H), 2.83 (dd, *J* = 7.6, 9.2 Hz, 1H), 2.71 - 2.56 (m, 4H), 2.47 - 2.36 (m, 1H), 2.36 - 2.28 (m, 1H), 2.10 - 1.98 (m, 1H), 1.54 - 1.49 (m, 1H), 1.36 (s, 9H). **^{13}C NMR** (101 MHz, MeOD) δ 158.15, 148.17, 137.30, 123.60, 122.48, 61.02, 58.44, 53.94, 53.49, 43.92, 37.17, 29.12, 28.69. **LCMS** 2.54, 98 %, *m/z* 328.2 $[M+H]^+$. **HRMS** (ES+): calcd. for $C_{19}H_{30}N_5$ $[M+H]^+$ 328.2496, found 328.2499 (1.14 ppm).

tert-butyl ((1-(pyridin-2-ylmethyl)piperidin-3-yl)methyl)carbamate (Intermediate 1, for synthesis of compound 16)

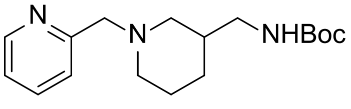

tert-butyl ((1-(pyridin-2-ylmethyl)piperidin-3-yl)methyl)carbamate was prepared according to general procedure 3 from *tert*-butyl *N*-(3-piperidylmethyl)carbamate (500 mg, 2.33 mmol, 1.0 equiv.) and pyridine-2-carbaldehyde (249 mg, 2.33 mmol, 1.0 equiv.) in MeOH (0.23 M) using 1 equiv. of AcOH. The reaction was quenched with water, concentrated and purified by reverse phase flash column chromatography (5-95 % CH_3CN in water (+0.1 % FA)) to afford *tert*-butyl *N*-[[1-(2-pyridylmethyl)-3-piperidyl]methyl]carbamate (200 mg, 0.65 mmol, 25 %) as a yellow oil. **^{1}H NMR** (400 MHz, $CDCl_3$) δ 8.60 - 8.53 (m, 1H), 7.65 (dt, *J* = 1.6, 7.6 Hz, 1H), 7.42 (br d, *J* = 5.6 Hz, 1H), 7.21 - 7.13 (m, 1H), 4.75 - 4.57 (m, 1H), 3.65 (br s, 2H), 3.16 - 2.94 (m, 2H), 2.89 - 2.64 (m, 2H), 1.97 - 1.53 (m, 6H), 1.43 (s, 9H), 1.17 - 0.92 (m, 1H). **LCMS** (ES+): *m/z* 306.3 $[M+H]^+$, 100 %.

***tert*-butyl ((1-(pyridin-2-ylmethyl)piperidin-3-yl)methyl)carbamate (Intermediate 2, for synthesis of compound 16)**

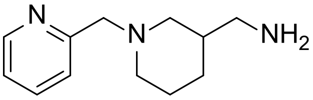

tert-butyl ((1-(pyridin-2-ylmethyl)piperidin-3-yl)methyl)carbamate was prepared according to general procedure 4 from *tert*-butyl *N*-[[1-(2-pyridylmethyl)-3-piperidyl]methyl]carbamate (200 mg, 0.65 mmol 1.0 equiv.) in CH_3CN (0.33 M) to afford *tert*-butyl ((1-(pyridin-2-ylmethyl)piperidin-3-yl)methyl)carbamate (158 mg, 0.65 mmol, 94 %) as a white solid which was used without further purification. **LCMS** (ES+): *m/z* 206.2 $[M+H]^+$, 100 %.

1-(3-(*tert*-butyl)-1*H*-pyrazol-4-yl)-*N*-((1-(pyridin-2-ylmethyl)piperidin-3-yl)methyl)methanamine (16)

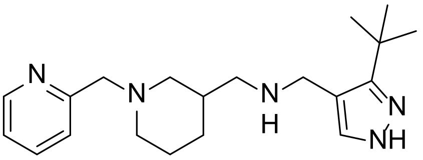

16 was prepared according to general procedure 3 from [1-(2-pyridylmethyl)-3-piperidyl]methanamine HCl (330 mg, 1.36 mmol, 1.0 equiv.) and 3-*tert*-butyl-1*H*-pyrazole-4-carbaldehyde (186 mg, 1.23 mmol, 0.9 equiv.) in MeOH (0.2 M) using 1 equiv. AcOH in addition to NaOAc (2 equiv), the reaction was quenched with water, concentrated and purified by preparative HPLC (15-45 % CH_3CN in water (+1 % NH_4HCO_3) using Phenomenex Gemini-NX C18 75 * 30 mm * 3 µm column) to afford desired compound (87 mg, 0.25 mmol, 18 %) as a white solid. **^{1}H NMR** (500 MHz, MeOD) δ 8.47 (d, *J* = 4.8 Hz, 1H), 7.85 - 7.80 (m, 1H), 7.56 - 7.52 (m, 2H), 7.32 (dd, *J* = 5.6, 6.9 Hz, 1H), 3.91 - 3.89 (m, 2H), 3.68 - 3.67 (m, 2H), 2.94 (d, *J* = 7.2 Hz, 1H), 2.81 (d, *J* = 11.1 Hz, 1H), 2.69 (d, *J* = 5.8 Hz, 2H), 2.21 - 2.13 (m, 1H), 1.94 (t, *J* = 6.9 Hz, 2H), 1.86 - 1.80 (m, 1H), 1.76 - 1.60 (m, 2H), 1.37 (s, 9H). **^{13}C NMR** (126 MHz, MeOD) δ 157.8, 148.2, 137.2, 123.8, 122.6, 112.5, 63.8, 57.9, 53.9, 52.4, 43.7, 35.0, 31.9, 29.2, 28.3, 24.1. **LCMS** 2.10, 99 %, *m/z* 342.3 $[M+H]^+$. **HRMS** (ES+): calcd. for $C_{20}H_{31}N_5$ $[M+H]^+$ 342.2652, found 342.2661 (2.6 ppm).

***tert*-butyl 3-(((3-(*tert*-butyl)-1*H*-pyrazol-4-yl)methyl)amino)-9-azabicyclo[3.3.1]nonane-9-carboxylate (Intermediate 1, for synthesis of compound 17)**

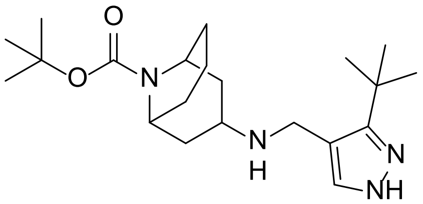

tert-butyl 3-(((3-(*tert*-butyl)-1*H*-pyrazol-4-yl)methyl)amino)-9-azabicyclo[3.3.1]nonane-9-carboxylate was prepared according to general procedure 3 from *tert*-butyl 3-amino-9-azabicyclo[3.3.1]nonane-9-carboxylate (500 mg, 2.08 mmol, 1.0 *eq*uiv.) and 3-*tert*-butyl-1*H*-pyrazole-4-carbaldehyde (253 mg, 1.66 mmol, 0.8 *eq*uiv.) in MeOH (0.21 M) using 1 equiv. of AcOH. The crude was purified by reverse phase flash column chromatography (5-95 % CH_3CN in water (+0.1 % FA)) to afford the desired compound (350 mg, 0.93 mmol, 42 %) as a yellow oil. **^{1}H NMR** (400 MHz, MeOD) δ 7.50 (br s, 1H), 4.41 (br d, *J* = 10.4 Hz, 2H), 3.79 (d, *J* = 2.0 Hz, 2H), 2.52 - 2.41 (m, 1H), 2.41 - 2.27 (m, 2H), 2.12 - 2.02 (m, 1H), 1.66 - 1.46 (m, 5H), 1.43 (s, 9H), 1.37 (s, 9H), 1.30 - 1.25 (m, 2H). **LCMS** (ES+): *m/z* 377.4 $[M+H]^+$, 100 %.

***N*-((3-(*tert*-butyl)-1*H*-pyrazol-4-yl)methyl)-9-azabicyclo[3.3.1]nonan-3-amine (Intermediate 2, for synthesis of compound 17)**

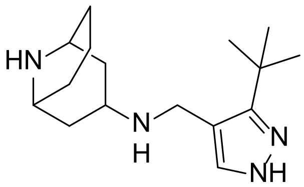

N-((3-(*tert*-butyl)-1*H*-pyrazol-4-yl)methyl)-9-azabicyclo[3.3.1]nonan-3-amine was prepared according to general procedure 4 from *tert*-butyl 3-(((3-(*tert*-butyl)-1*H*-pyrazol-4-yl)methyl)amino)-9-azabicyclo[3.3.1]nonane-9-carboxylate (340 mg, 0.90 mmol, 1.0 *equiv.)* in MeOH (0.23 M) to afford the desired produce as a yellow solid (300 mg, crude, HCl) which was used without further purification. **^{1}H NMR** (400 MHz, MeOD) δ 8.27 (s, 1H), 4.45 (s, 2H), 4.03 - 3.93 (m, 2H), 3.90 - 3.76 (m, 1H), 2.82 (td, *J* = 6.0, 12.0 Hz, 2H), 2.23 - 2.08 (m, 1H), 2.01 - 1.89 (m, 4H), 1.83 - 1.69 (m, 3H), 1.49 (s, 9H). **LCMS** (ES+): *m/z* 277.2 $[M+H]^+$, 100 %.

***N*-((3-(*tert*-butyl)-1*H*-pyrazol-4-yl)methyl)-9-(pyridin-2-ylmethyl)-9-azabicyclo[3.3.1]nonan-3-amine (17)**

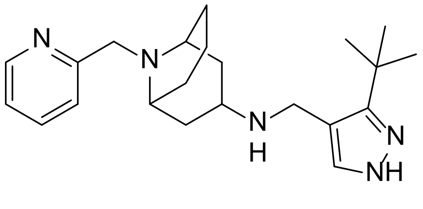

17 was prepared according to general procedure 3 from *N*-((3-(*tert*-butyl)-1*H*-pyrazol-4-yl)methyl)-9-azabicyclo[3.3.1]nonan-3-amine HCl (280 mg, 0.89 mmol, 1.0 *eq*uiv. HCl) and pyridine-2-carbaldehyde (96 mg, 0.89 mmol, 1.0 *equiv.*) in MeOH (0.1 M) using 1 equiv. of AcOH in addition to NaOAc (2 equiv.). The crude was purified by preparative NPLC (1-40 % EtOH in hexane (+0.1 % NH_4OH) using Welch Ultimate XB-CN 250 * 50 * 10 µm column) followed by preparative HPLC (32-62 % CH_3CN in water (+1 % NH_4HCO_3)) to afford desired compound **17** (74 mg, 0.19 mmol, 22 %) as a yellow solid. **^{1}H NMR** (500 MHz, MeOD) δ 8.45 - 8.42 (m, 1H), 7.85 -

7.80 (m, 1H), 7.60 - 7.57 (m, 2H), 7.31 - 7.27 (m, 1H), 3.98 (s, 2H), 3.89 (s, 2H), 3.10 (d, *J* = 10.9 Hz, 2H), 2.49 - 2.40 (m, 2H), 2.15 - 1.97 (m, 3H), 1.57 (d, *J* = 13.0 Hz, 1H), 1.49 - 1.44 (m, 1H), 1.42 - 1.40 (m, 9H), 1.37 - 1.29 (m, 2H), 1.11 (br d, *J* = 12.7 Hz, 2H). **^{13}C NMR** (126 MHz, MeOD) δ 160.6, 147.8, 137.4, 122.8, 122.1, , 57.0, 50.1, 49.0, 41.0, 32.2, 29.3, 24.9, 13.8. **LCMS** 2.90, 97 %, *m/z* 368.2 [M+H]$^+$. **HRMS** (ES+): calcd. for $C_{22}H_{33}N_5$ [M+H]$^+$ 368.2809, found 368.2806 (0.7 ppm).

18 was prepared following the same procedure as 17 from *tert*-butyl 3-amino-8-azabicyclo[3.2.1]octane-8-carboxylate (500 mg, 2.21 mmol, 1.0 *eq*uiv.) and 3-*tert*-butyl-1*H*-pyrazole-4-carbaldehyde (268 mg, 1.77 mmol, 0.8 *eq*uiv.).

***tert*-butyl 3-(((3-(*tert*-butyl)-1*H*-pyrazol-4-yl)methyl)amino)-8-azabicyclo[3.2.1]octane-8-carboxylate (Intermediate 1, compound 18)**

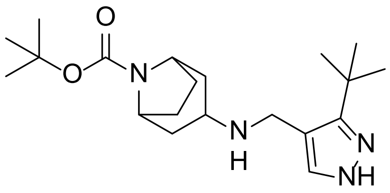

(360 mg, 0.99 mmol, 45 %) isolated as a white solid. **^{1}H NMR** (400 MHz, MeOD) δ 7.61 - 7.31 (m, 1H), 4.13 (br s, 2H), 3.73 (s, 2H), 3.03 - 2.89 (m, 1H), 2.23 - 2.02 (m, 4H), 1.90 (br s, 2H), 1.64 (br d, J = 14.4 Hz, 2H), 1.46 (s, 9H), 1.38 (br s, 9H). **LCMS** (ES+): m/z 363.3 [M+H]$^+$, 100 %.

***N*-((3-(*tert*-butyl)-1*H*-pyrazol-4-yl)methyl)-8-azabicyclo[3.2.1]octan-3-amine HCl (Intermediate 2, compound 18)**

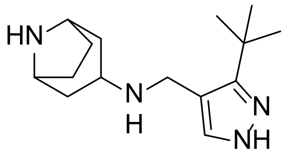

(300 mg, crude) isolated as a white solid and used without further purification. **^{1}H NMR** (400 MHz, MeOD) δ 8.50 (s, 1H), 4.48 (s, 2H), 4.15 (br d, *J* = 5.6 Hz, 2H), 3.87 - 3.70 (m, 1H), 2.80 (td, *J* = 7.6, 15.2 Hz, 2H), 2.31 - 2.18 (m, 6H), 1.50 (s, 9H). **LCMS** (ES+): *m/z* 263.2 [M+H]$^+$, 100 %.

***N*-((3-(*tert*-butyl)-1*H*-pyrazol-4-yl)methyl)-8-(pyridin-2-ylmethyl)-8-azabicyclo[3.2.1]octan-3-amine (18)**

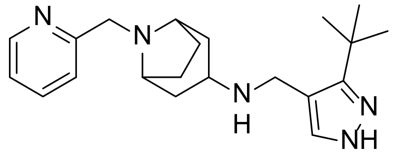

18 (40 mg, 0.11 mmol, 11 %) was isolated as a light yellow solid. **^{1}H NMR** (500 MHz, MeOD) δ 8.49 - 8.46 (m, 1H), 7.86 - 7.81 (m, 1H), 7.69 - 7.65 (m, 1H), 7.46 - 7.43 (m, 1H), 7.34 - 7.30 (m, 1H), 3.75 - 3.72 (m, 4H), 3.25 - 3.21 (m, 2H), 3.00 (t, *J* = 6.4 Hz, 1H), 2.20 - 2.06 (m, 6H), 1.66 (d, *J* = 14.3 Hz, 2H), 1.40 (s, 9H). **^{13}C NMR** (126 MHz, MeOD) δ 158.99, 148.05, 137.25, 123.35, 122.35, 58.09, 57.38, 49.94, 43.22, 35.82, 29.12, 26.13. **LCMS** 2.70, 91 %, *m/z* 354.2 [M+H]$^+$. **HRMS** (ES+): calcd. for $C_{21}H_{31}N_5$ [M+H]$^+$ 354.2652, found 354.2658 (1.5 ppm).

tert-butyl (1-(pyridin-2-ylmethyl)azepan-4-yl)carbamate (Intermediate 1, for synthesis of compound 19)

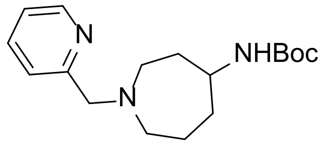

tert-butyl (1-(pyridin-2-ylmethyl)azepan-4-yl)carbamate was prepared according to general procedure 3 from *tert*-butyl azepan-4-ylcarbamate HCl (500 mg, 1.99 mmol, 1.0 *eq*uiv. HCl) and pyridine-2-carbaldehyde (213 mg, 1.99 mmol, 1.0 *eq*uiv.) in MeOH (0.4 M) using 1 equiv. of AcOH in addition to AcONa (2 equiv.) and extracted with 10:1 DCM:MeOH to afford crude *tert*-butyl (1-(pyridin-2-ylmethyl)azepan-4-yl)carbamate (500 mg, 1.64 mmol, 78 %) as a yellow oil which was used without further purification. **LCMS** (ES+): *m/z* 306.4 $[M+H]^+$, 100 %.

1-(pyridin-2-ylmethyl)azepan-4-amine hydrochloride (Intermediate 2, for synthesis of compound 19)

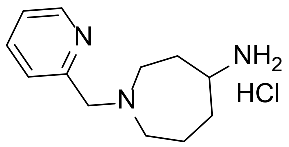

1-(pyridin-2-ylmethyl)azepan-4-amine hydrochloride was prepared according to general procedure 4 from *tert*-butyl (1-(pyridin-2-ylmethyl)azepan-4-yl)carbamate (500 mg, 1.64 mmol, 1.0 *equiv.) in CH_3CN (0.33 M)* to afford 1-(pyridin-2-ylmethyl)azepan-4-amine HCl (400 mg, crude, HCl) as a yellow solid which was used without further purification. **^{1}H NMR** (400 MHz, MeOD) δ 8.77 (dd, *J* = 0.8, 5.2 Hz, 1H), 8.12 (dt, *J* = 1.6, 7.6 Hz, 1H), 7.80 (d, *J* = 8.0 Hz, 1H), 7.65 (dt, *J* = 0.8, 6.0 Hz, 1H), 4.67 (s, 2H), 3.64 - 3.60 (m, 1H), 3.59 - 3.46 (m, 4H), 2.35 - 2.20 (m, 3H), 2.15 - 2.11 (m, 1H), 2.09 - 2.01 (m, 1H), 1.83 - 1.79 (m, 1H). **LCMS** (ES+): *m/z* 206.2 $[M+H]^+$, 100 %.

***N*-((3-(*tert*-butyl)-1*H*-pyrazol-4-yl)methyl)-1-(pyridin-2-ylmethyl)azepan-4-amine hydrochloride (19)**

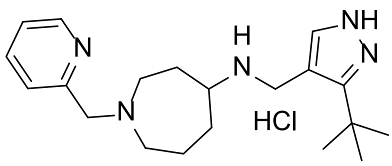

19 was prepared according to general procedure 3 from 1-(2-pyridylmethyl)azepan-4-amine hydrochloride (400 mg, 1.65 mmol, 1.0 *eq*uiv.) and 3-*tert*-butyl-1*H*-pyrazole-4-carbaldehyde (176 mg, 1.16 mmol, 0.7 *eq*uiv.) in MeOH (0.3 M) using 1 equiv. of AcOH in addition to NaOAc (2 equiv.) and extracted with 10:1 DCM:MeOH. The crude was purified by preparative HPLC (1-21 % CH_3CN in water (+0.05 % HCl) using 3_Phenomenex Luna C18 75 * 30 mm * 3 μm column) to afford the desired compound (127 mg, 0.37 mmol, 15 %) as a white solid. **^{1}H NMR** (500 MHz, MeOD) δ 8.93 (d, *J* = 5.0 Hz, 1H), 8.60 (s, 1H), 8.46 (t, *J* = 7.6 Hz, 1H), 8.20 (d, *J* = 7.6 Hz, 1H), 7.96 (t, *J* = 6.3 Hz, 1H), 4.88 - 4.85 (m, 2H), 3.89 - 3.80 (m, 2H), 3.70 - 3.57 (m, 3H), 2.74 - 2.68 (m, 1H), 2.61 - 2.50 (m, 2H), 2.32 - 2.22 (m, 1H), 2.17 - 2.05 (m, 2H), 1.54 (s, 9H). **^{13}C NMR** (126 MHz, MeOD) δ 154.7, 146.0, 145.9, 143.6, 136.1, 128.1, 126.7, 110.7, 57.6, 54.9, 50.5, 39.2, 32.7, 28.7, 28.6, 25.6, 19.7. **LCMS** 2.52, 99 %, *m/z* 342.3 $[M+H]^+$. **HRMS** (ES+): calcd. for $C_{20}H_{31}N_5$ $[M+H]^+$ 342.2652, found 342.2658 (1.6 ppm).

1-benzyl-*N*-((3-(*tert*-butyl)-1*H*-pyrazol-4-yl)methyl)piperidin-4-amine (20)

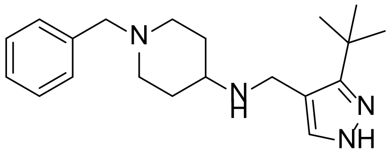

20 was prepared according to general procedure 3 from commercial 1-benzylpiperidin-4-amine (375 mg, 1.97 mmol, 402 µL, 1.5 equiv.) and 3-*tert*-butyl-1*H*-pyrazole-4-carbaldehyde (200 mg, 1.31 mmol, 1.0 equiv.) in EtOH (0.3 M). The crude residue was purified by preparative HPLC (32-62 % CH_3CN in water (+1 % NH_4HCO_3)) to afford desired compound (13.9 mg, 0.041 mmol, 3 %) as colorless gum. **^{1}H NMR** (400 MHz, MeOD) δ 7.57 - 7.45 (m, 1H), 7.32 (d, *J* = 4.4 Hz, 4H), 7.29 - 7.25 (m, 1H), 3.80 (s, 2H), 3.52 (s, 2H), 2.92 (br d, *J* = 11.6 Hz, 2H), 2.65 - 2.52 (m, 1H), 2.12 - 2.03 (m, 2H), 1.99 - 1.90 (m, 2H), 1.55 - 1.42 (m, 2H), 1.37 - 1.36 (s, 9H). **LCMS** 1.69, 100 %, *m/z* 327.4 $[M+H]^+$. **HRMS** (ES+): calcd. For $C_{20}H_{31}N_4$ $[M+H]^+$ 327.2544, found 327.2547 (1.29 ppm).

***N*-((3-(*tert*-butyl)-1*H*-pyrazol-4-yl)methyl)-1-((3-methoxypyridin-2-yl)methyl)piperidin-4-amine (21)**

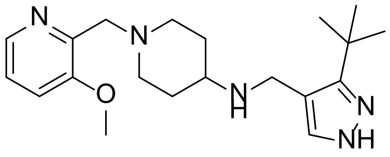

21 was prepared according to general procedure 5 from **36** (100 mg, 0.42 mmol, 1 equiv.) and 3-methoxypyridine-2-carbaldehyde (58 mg, 0.42 mmol, 1 equiv.) in EtOH (0.4 M) to afford the desired compound (37 mg, 0.098 mmol, 23 %) as a clear sticky glass. **^{1}H NMR** (500 MHz, MeOD) δ 8.13 – 8.09 (m, 1H), 7.53 (br s, 1H), 7.46 (d, *J* = 8.2 Hz, 1H), 7.37 – 7.32 (m 1H), 3.9 (s, 3H), 3.85 (s, 2H), 3.74, (s 2H), 3.06 – 3.01 (m, 2H), 2.68 – 2.60 (m, 1H), 2.29 – 2.21 (m, 2H), 1.99 – 1.92 (m, 2H), 1.59 – 1.49 (m, 2H), 1.39 (s, 9H). **^{13}C NMR** (126 MHz, MeOD) δ 155.0, 146.2, 139.5, 123.8, 118.3, 57.0, 54.7, 54.4, 52.2, 40.6, 30.8, 29.1. **LCMS** 1.42, 99 %, *m/z* 358.3 $[M+H]^+$. **HRMS** (ES+): calcd. for $C_{20}H_{32}N_5O$ $[M+H]^+$ 358.2601, found 358.2600 (0.5 ppm).

***N*-((3-(*tert*-butyl)-1*H*-pyrazol-4-yl)methyl)-1-((4-methoxypyridin-2-yl)methyl)piperidin-4-amine (22)**

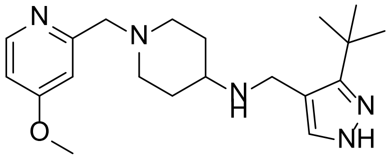

22 was prepared according to general procedure 5 from **36** (100 mg, 0.42 mmol, 1 equiv.) and 4-methoxypicolinaldehyde (58 mg, 0.42 mmol, 1 equiv.) in EtOH (0.4 M) to afford desired compound **22** (9 mg, 0.023 mmol, 5.7 %) as a clear sticky glass. **^{1}H NMR** (500 MHz, DMSO-*d$_6$*) δ 12.07 (s, 1H), 8.30 - 8.28 (m, 1H), 7.41 - 7.34 (m, 1H), 6.97 (d, J = 2.4 Hz, 1H), 6.84 (dd, *J* =2.6, 5.8 Hz, 1H), 3.82 (s, 3H), 3.66 (s, 2H), 3.52 - 3.50 (m, 2H), 2.81 - 2.77 (m, 2H), 2.45 (s, 1H), 2.08 - 2.03 (m, 2H), 1.84 - 1.81 (m, 2H), 1.32 - 1.30 (m, 11H). **LCMS** 0.74, 94 %, *m/z* 358.1 $[M+H]^+$. **HRMS** (ES+): calcd. For $C_{20}H_{32}ON_5$ $[M+H]^+$ 358.2602, found 358.2606 (1.36 ppm).

***N*-((3-(*tert*-butyl)-1*H*-pyrazol-4-yl)methyl)-1-((5-methoxypyridin-2-yl)methyl)piperidin-4-amine (23)**

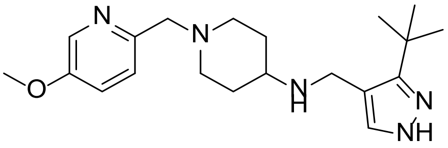

23 was prepared according to general procedure 5 from **36** (100 mg, 0.42 mmol, 1 equiv.) and 5-methoxypicolinaldehyde (58 mg, 0.42 mmol, 1 equiv.) in EtOH (0.8 M) to afford the desired compound (19 mg, 0.050 mmol, 12 %) as a clear sticky glass. **^{1}H NMR** (500 MHz, MeOD) δ 8.18 – 8.16 (m, 1H), 7.53 (br s, 1H), 7.47 (d, *J* = 8.4 Hz, 1H), 7.43 – 7.39 (m, 1H), 3.88 (s, 3H), 3.81 (s, 2H), 3.60, (s 2H), 2.94 – 2.89 (m, 2H), 2.64 – 2.56 (m, 1H), 2.18 – 2.11 (m, 2H), 1.98 – 1.92 (m, 2H), 1.55 – 1.46 (m, 2H), 1.38 (s, 9H). **^{13}C NMR** (126 MHz, MeOD) δ 155.4, 149.5, 135.6, 124.4, 121.6, 62.7, 54.9, 54.5, 52.1, 40.7, 31.3, 29.1. **LCMS** 0.77, 96 %, *m/z* 358.3 [M+H]$^+$. **HRMS** (ES+): calcd. for $C_{20}H_{32}N_5O$ [M+H]$^+$ 358.2601, found 358.2590 (3.1 ppm).

***N*-((3-(*tert*-butyl)-1*H*-pyrazol-4-yl)methyl)-1-((6-methoxypyridin-2-yl)methyl)piperidin-4-amine, (24)**

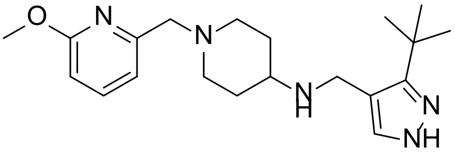

24 was prepared according to general procedure 5 from **36** (100 mg, 0.42 mmol, 1 equiv.) and 6-methoxypyridine-2-carbaldehyde (58 mg, 0.42 mmol, 1 equiv.) in EtOH (0.8 M) to afford the desired compound (16 mg, 0.043 mmol, 10 %) as a sticky clear solid. **^{1}H NMR** (500 MHz, MeOD) δ 7.65 – 7.60 (m, 1H), 7.53 (br s, 1H), 7.01 (d, *J* = 7.2 Hz, 1H), 6.67 (d, J = 8.2 Hz, 1H), 3.92 (s, 3H), 3.81 (s, 2H), 3.58 (s 2H), 3.01 – 2.96 (m, 2H), 2.63 – 2.56 (m, 1H), 2.23 – 2.16 (m, 2H), 1.99 – 1.93 (m, 2H), 1.58 – 1.48 (m, 2H), 1.39 (s, 9H). **^{13}C NMR** (126 MHz, MeOD) δ 163.9, 155.5, 138.9, 116.0, 108.2, 63.4, 54.5, 52.5, 52.3, 40.8, 31.3, 29.1. **LCMS** 0.81, 98 %, *m/z* 358.3 [M+H]$^+$. **HRMS** (ES+): calcd. for $C_{20}H_{32}N_5O$ [M+H]$^+$ 358.2601, found 358.2600 (0.4 ppm).

***N*-((3-(*tert*-butyl)-1*H*-pyrazol-4-yl)methyl)-1-((3-methylpyridin-2-yl)methyl)piperidin-4-amine (25)**

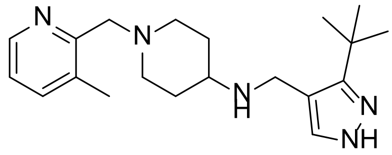

25 was prepared according to general procedure 5 from **36** (100 mg, 0.42 mmol, 1 equiv.) and 3-methylpyridine-2-carbaldehyde (51 mg, 0.42 mmol, 1 equiv.) in EtOH (0.8 M) to afford desired compound **25** (17 mg, 0.047 mmol, 11 %) as a sticky clear oil. **^{1}H NMR** (500 MHz, MeOD) δ 8.31 – 8.27 (m, 1H), 7.66 – 7.61 (m, 1H), 7.53 (br s, 1H), 7.27 - 7.23 (m, 1H), 3.80 (s, 2H), 3.65 (s, 2H), 2.91 – 2.85 (m, 2H), 2.63 – 2.56 (m, 1H), 2.46 (s, 3H), 2.22 – 2.14 (m, 2H), 1.96 – 1.88 (m, 2H), 1.50 – 1.41 (m, 2H), 1.39 (s, 9H). **^{13}C NMR** (126 MHz, MeOD) δ 156.1, 144.9, 138.8, 134.2, 122.7, 61.8, 54.7, 52.4, 40.8, 31.6, 29.1, 17.1. **LCMS** 0.76, 97 %, *m/z* 342.1 [M+H]$^+$. **HRMS** (ES+): calcd. for $C_{20}H_{32}N_5$ [M+H]+ 342.2652, found 342.2641 (3.3 ppm).

***N*-((3-(*tert*-butyl)-1*H*-pyrazol-4-yl)methyl)-1-((3-fluoropyridin-2-yl)methyl)piperidin-4-amine (26)**

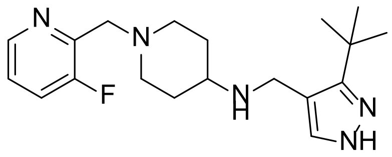

26 was prepared according to general procedure 5 from **36** (100mg, 0.42 mmol, 1 equiv.) and 3-fluoropicolinaldehyde (53 mg, 0.42 mmol, 1 equiv.) in EtOH (0.8 M) to afford the desired compound (7 mg, 0.019 mmol, 4.5 %) as a sticky clear glass. **^{1}H NMR** (500 MHz, MeOD) δ 8.40 - 8.39 (m, 1H), 7.64 - 7.59 (m, 1H), 7.52 (s, 1H), 7.44 - 7.40 (m, 1H), 3.81 (s, 2H), 3.76 (d, J = 2.6 Hz, 2H), 2.99 (d, *J* = 12.1 Hz, 2H), 2.62 - 2.56 (m, 1H), 2.27 - 2.20 (m, 2H), 1.95 - 1.91 (m, 2H), 1.55 - 1.46 (m, 2H), 1.40 - 1.37 (m, 9H). **LCMS** 0.76, 97 %, *m/z* 346.0 $[M+H]^+$. **HRMS** (ES+): calcd. for $C_{19}H_{28}N_5F$ [M+H]+ 346.2402, found 346.2405 (1.06 ppm).

2-((4-(((3-(*tert*-butyl)-1*H*-pyrazol-4-yl)methyl)amino)piperidin-1-yl)methyl)pyridin-3-ol (27)

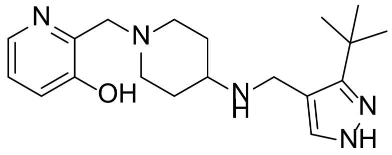

27 was prepared according to general procedure 2 from **36** (586 mg, 2.47 mmol, 1 equiv.) and 3-hydroxypicolinaldehyde (305 mg, 2.47 mmol, 1 equiv.) in DCM (0.25 M) using 1.5 equiv. of $NaBH(OAc)_3$ the residue was purified by preparative HPLC (5-95 % MeCN in water (+0.1 % NH_4OH)) to afford the desired compound (18 mg, 0.05 mmol, 33 %). **^{1}H NMR** (500 MHz, MeOD) δ 7.95 – 7.92 (m, 1H), 7.54 (s, 1H), 7.23 – 7.19 (m, 1H), 7.18 – 7.15 (m, 1H), 3.91 (s, 2H), 3.82 (s, 2H), 3.05 – 2.99 (m, 2H), 2.73 – 2.66 (m, 1H), 2.35 – 2.28 (m, 2H), 2.07 – 2.01 (m, 2H), 1.58 – 1.49 (m, 2H), 1.39 (s, 9H). **^{13}C NMR** (126 MHz, MeOD) δ 154.9, 142.5, 138.7, 123.8, 123.4, 114.6, 61.9, 53.9, 51.8, 40.9, 31.4, 29.1. **LCMS** 1.39, 97 %, *m/z* 344.2 $[M+H]^+$. **HRMS** (ES+): calcd. for $C_{19}H_{30}N_5O$ $[M+H]^+$ 344.2445, found 344.2461 (4.7 ppm).

***N*-((3-(*tert*-butyl)-1*H*-pyrazol-4-yl)methyl)-1-((3-isopropoxypyridin-2-yl)methyl)piperidin-4-amine (28)**

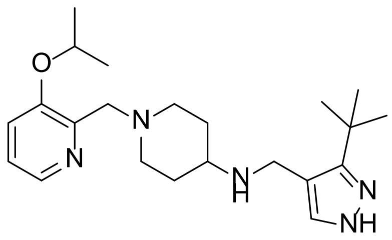

To a solution of **27** (115 mg, 0.33 mmol, 1 equiv.) in DMF (0.17 M) was added K_2CO_3 (93 mg, 0.67 mmol, 2 equiv.) and 2-bromopropane (45 mg, 0.37 mmol, 1.1 equiv.). The reaction heated in a sealed tube at 80 °C for 16 h and then diluted with MeOH and transferred to SCX washing with MeOH and eluting product with NH_3 in MeOH, the combined organics were concentrated *in vacuo* and purified by preparative HPLC (5-95 % CH_3CN in water (+0.1% NH_4OH)) to afford the desired compound (31 mg, 0.076 mmol, 22 %). **^{1}H NMR** (500 MHz, MeOD) δ 7.90 – 7.87 (m, 1H), 7.32 (broad s, 1H), 7.27 – 7.22 (m, 2H), 7.13 – 7.08 (m, 2H), 4.53 – 4.46 (m, 1H), 3.61, (2, 2H), 3.52 (s, 2H), 2.86 – 2.80 (m, 2H), 2.41 – 2.33 (m, 1H), 2.08 – 2.01 (m, 2H), 1.76 – 1.70 (m, 2H), 1.35 – 1.26 (m, 2H), 1.20 – 1.15 (m, 15H). **^{13}C NMR** (126 MHz, MeOD) δ 153.2, 147.2, 139.4, 123.5, 120.5,

70.5, 56.9, 54.4, 52.2, 40.7, 31.2, 29.1, 20.8. **LCMS** 2.04, 97 %, *m/z* 386.4 [M+H]$^+$. **HRMS** (ES+): calcd. for $C_{22}H_{36}N_5O_1$ [M+H]$^+$ 386.292, found 386.2932 (3.1 ppm).

_N_-((3-(_tert_-butyl)-1_H_-pyrazol-4-yl)methyl)-1-((3-ethoxypyridin-2-yl)methyl)piperidin-4-amine (29)

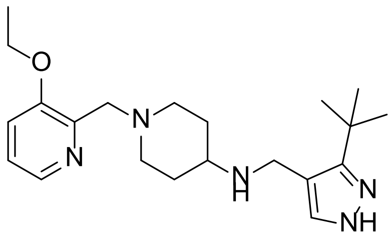

28 was prepared according to general procedure 2 from **36**(50 mg, 0.21 mmol, 1 equiv.) and 3-ethoxypicolinaldehyde (32 mg, 0.21 mmol, 1 equiv.) in DCM (0.2 M) using 1.5 equiv. of $NaBH(OAc)_3$ to afford the desired compound (33 mg, 0.08 mmol, 40 %). **^{1}H NMR** (500 MHz, MeOD) δ 8.11 – 8.08 (m, 1H), 7.52 (br s, 1H), 7.46 – 7.41 (m, 1H), 7.34 – 7.30 (m, 1H), 4.16 – 4.11 (m, 2H), 3.82 (br s, 2H), 3.75 (br s, 2H), 3.08 – 3.01 (m, 2H), 2.63 – 2.56 (m, 1H), 2.29 – 2.21 (m, 2H), 1.98 – 1.90 (m, 2H), 1.57 – 1.49 (m, 2H), 1.49 – 1.43 (m, 3H), 1.39 (br s, 9H). **^{13}C NMR** (126 MHz, MeOD) δ 152.9, 144.9, 137.9, 122.2, 117.7, 62.3, 55.4, 52.9, 50.7, 39.3, 29.5, 27.6, 12.1. **LCMS** 1.63, 100 %, *m/z* 372.3 [M+H]$^+$. **HRMS** (ES+): calcd. for $C_{22}H_{36}N_5O_1$ [M+H]$^+$ 372.2763, found 372.2763 (3.2 ppm).

1-((1_H_-benzo[d]imidazol-7-yl)methyl)-_N_-((3-(_tert_-butyl)-1_H_-pyrazol-4-yl)methyl)piperidin-4-amine (30)

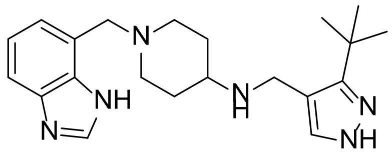

30 was prepared according to general procedure 2 from **36** (50 mg, 0.21 mmol, 1 equiv.) and 3*H*-benzimidazole-4-carbaldehyde (31 mg, 0.21 mmol, 1 equiv.) in DCM (0.2 M) using 1.5 equiv. of $NaBH(OAc)_3$ to afford the desired compound (25 mg, 0.06 mmol, 31 %)**.** **^{1}H NMR** (500 MHz, MeOD) δ 8.18 (s, 1H), 7.61 – 7.54 (m, 2H), 7.29 – 7.21 (m, 2H), 3.98 – 3.88 (m, 4H), 3.07 – 2.97 (m, 2H), 2.85 – 2.76 (m, 1H), 2.27 – 2.16 (m, 2H), 2.05 – 1.97 (m, 2H), 1.65 – 1.55 (m 2H), 1.39, (s, 9H). **^{13}C NMR** (126 MHz, MeOD) δ 141.1, 123.3, 122.1, 58.2, 54.8, 51.8, 40.5, 30.4, 29.1. **LCMS** 1.86, 96 %, *m/z* 367.5 [M+H]$^+$. **HRMS** (ES+): calcd. for $C_{21}H_{31}N_6$ [M+H]$^+$ 367.261, found 367.2609 (0.3 ppm).

1-((1_H_-imidazol-5-yl)methyl)-_N_-((3-(_tert_-butyl)-1_H_-pyrazol-4-yl)methyl)piperidin-4-amine (31)

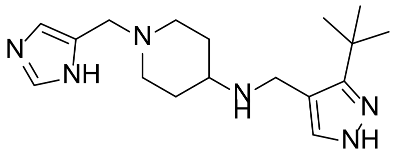

31 was prepared according to general procedure 3 from **36** (0.2 g, 0.846 mmol, 1.0 equiv.) and 1*H*-imidazole-5-carbaldehyde (81 mg, 0.85 mmol, 1.0 equiv.) in MeOH (0.2 M) and stirred for 0.5 h prior to adding $NaBH_3CN$. The reaction was purified without workup by preparative HPLC (7-37 % CH_3CN in water (+1% NH_4HCO_3)) followed by 1-20 % CH_3CN in water (+0.1 % Formic acid) using Waters Atlantis T3 150 * 30 mm * 5 um column and finally 11-41 % CH_3CN in water (+ 0.1%

NH_4OH) to afford the desired compound (44.2 mg, 0.14 mmol, 16 %) as a light-yellow gum. **^{1}H NMR** (500 MHz, MeOD) δ 7.61 (s, 1H), 7.50 (br s, 1H), 6.98 (s, 1H), 3.78 (s, 2H), 3.54, (s, 2H), 2.97 – 2.91 (m, 2H), 2.59 – 2.52 (m, 1H), 2.15 – 2.07 (m, 2H), 1.98 – 1.91 (m, 2H), 1.52 – 1.41 (m, 2H), 1.36 (s, 9H). **^{13}C NMR** (126 MHz, MeOD) δ 134.8, 54.4, 51.6, 40.8, 31.2, 29.1. **LCMS** 1.91, 100 %, *m/z* 317.3 $[M+H]^+$. **HRMS** (ES+): calcd. for $C_{17}H_{28}N_6$ $[M+H]^+$ 317.2454, found 317.2456 (0.6 ppm).

Preparation of *tert*-butyl 4-(((3-(*tert*-butyl)-1*H*-pyrazol-4-yl)methyl)amino)azepane-1-carboxylate (Intermediate 1, for synthesis of compound 32)

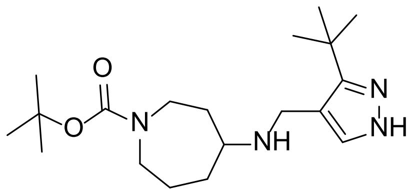

tert-butyl 4-(((3-(*tert*-butyl)-1*H*-pyrazol-4-yl)methyl)amino)azepane-1-carboxylate was prepared according to general procedure 3 from *tert*-butyl 4-aminoazepane-1-carboxylate (6.97 g, 32.5 mmol, 1.1 *equiv.)* and *tert*-butyl-1*H*-pyrazole-4-carbaldehyde (4.50 g, 29.5 mmol, 1.0 *equiv.)* in MeOH (0.3 M) using 1 equiv. of AcOH. The product was purified by reverse phase flash chromatography (5-95 % CH_3CN in water (0.1% NH_4OH)) to afford *tert*-butyl 4-[(3-*tert*-butyl-1*H*-pyrazol-4-yl)methylamino]azepane-1-carboxylate (9.1 g, 26.0 mmol, 88 %) as a yellow solid. **^{1}H NMR** (400 MHz, $CDCl_3$) δ 7.49 (s, 1H), 3.85 - 3.67 (m, 2H), 3.60 - 3.41 (m, 2H), 3.41 - 3.17 (m, 2H), 2.81 - 2.65 (m, 1H), 2.02 - 1.82 (m, 3H), 1.65 - 1.44 (m, 12H), 1.38 (s, 9H). **LCMS** (ES+): *m/z* 351.4 $[M+H]^+$, 100 %.

***N*-((3-(*tert*-butyl)-1*H*-pyrazol-4-yl)methyl)azepan-4-amine (Intermediate 2, for synthesis of compound 32)**

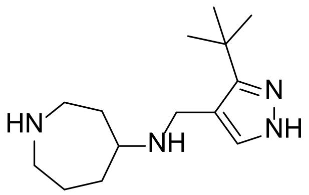

N-((3-(*tert*-butyl)-1*H*-pyrazol-4-yl)methyl)azepan-4-amine was prepared according to general procedure 4 from *tert*-butyl 4-[(3-*tert*-butyl-1*H*-pyrazol-4-yl)methylamino]azepane-1-carboxylate (9.0 g, 25.6 mmol, 1.0 *eq*uiv.) in CH_3CN (0.28 M). The residue was dissolved with water and the mixture was worked up with an anion exchange resin. The resulting residue was washed with water and the filtrate was lyophilized to afford the desired compound (6.5 g, 24.1 mmol, 94 %) as a yellow gum. **^{1}H NMR** (400 MHz, MeOD) δ 7.53 (s, 1H), 3.80 (s, 2H), 3.31-3.17 (m, 1H), 3.13 - 2.90 (m, 4H), 2.16 - 1.94 (m, 3H), 1.82 - 1.60 (m, 3H), 1.38 (s, 9H). **LCMS** (ES+): *m/z* 251.2 $[M+H]^+$, 93 %.

***N*-((3-(*tert*-butyl)-1*H*-pyrazol-4-yl)methyl)-1-((3-methoxypyridin-2-yl)methyl)azepan-4-amine (32)**

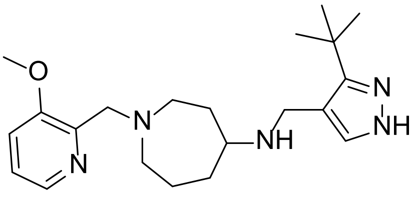

32 was prepared according to general procedure 3 from intermediate 2, compound 32 (200 mg, 0.798 mmol, 1 equiv.) and 3-methoxypyridine-2-carbaldehyde (109 mg, 0.79 mmol, 1 equiv.) in

MeOH (0.16 M). The reaction was purified without workup by preparative HPLC (23-53 % CH_3CN in water (+1 % NH_4HCO_3)) to afford the desired compound (80 mg, 0.21 mmol, 26 %) as a brown oil. **^{1}H NMR** (500 MHz, MeOD) δ 8.0 – 7.97 (m, 1H), 7.55 (b s, 1H), 7.45 – 7.41 (m, 1H), 7.33 – 7.29 (m, 1H), 3.87 (s, 4H), 3.85 (s, 3H), 3.06 – 2.99 (m, 1H), 2.99 – 2.93 (m, 1H), 2.84 – 2.79 (m, 2H), 2.77 – 2.70 (m, 1H), 2.01 – 1.89 (m 2H), 1.86 – 1.75 (m, 2H), 1.74 – 1.62 (m, 2H), 1.37 (2, 9H). **^{13}C NMR** (126 MHz, MeOD) δ 154.8, 147.2, 139.4, 123.6, 118.2, 57.1, 56.9, 55.2, 54.7, 50.9, 41.1, 32.4, 31.1, 29.1, 23.9**.** **LCMS** 2.06, 95 %, *m/z* 372.2 $[M+H]^+$. **HRMS** (ES+): calcd. for $C_{22}H_{36}N_5O_1$ $[M+H]^+$ 372.2763, found 372.2773 (2.7 ppm).

^{1}H NMR spectra of intermediate 34.HCl in DMSO-d_6

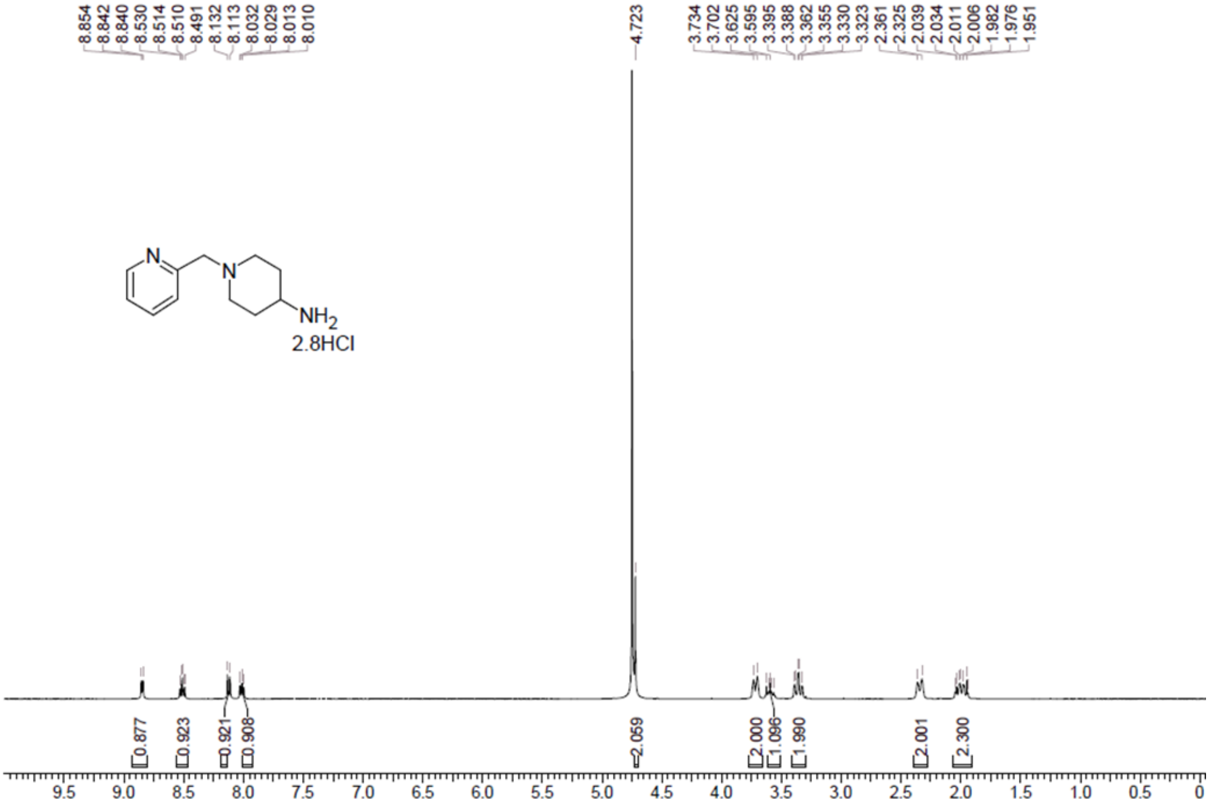

^{1}H NMR spectra of intermediate 34 in MeOD

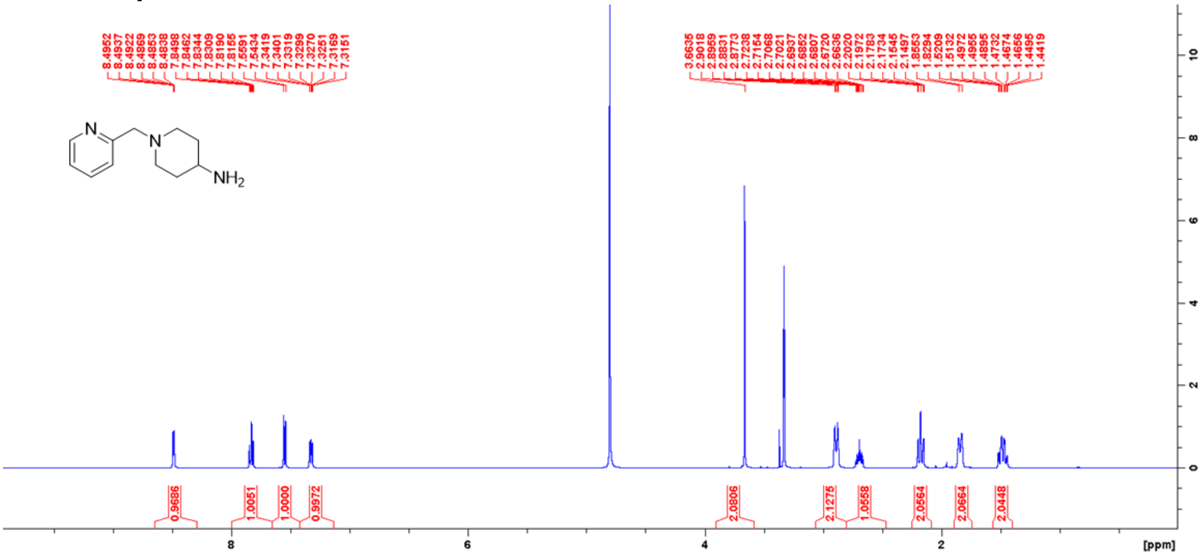

^{1}H NMR spectra of intermediate 36.HCl in D_2O

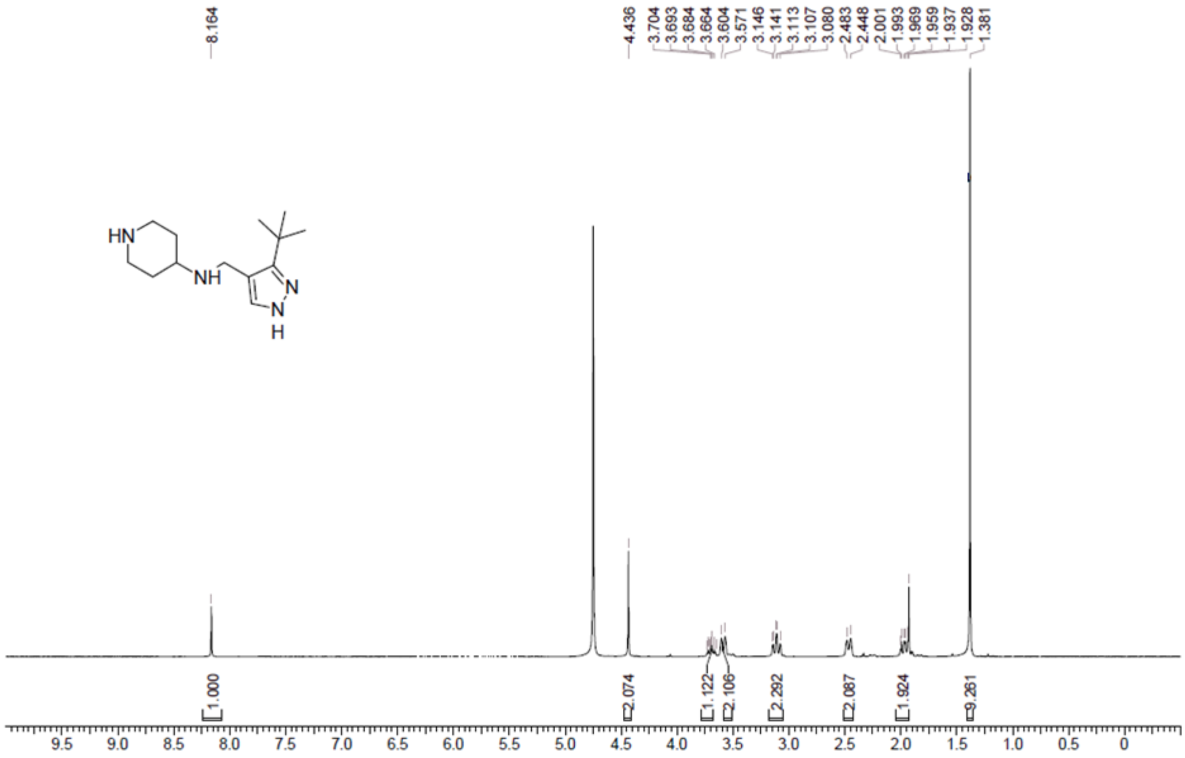

^{1}H NMR spectra of intermediate 36 in MeOD

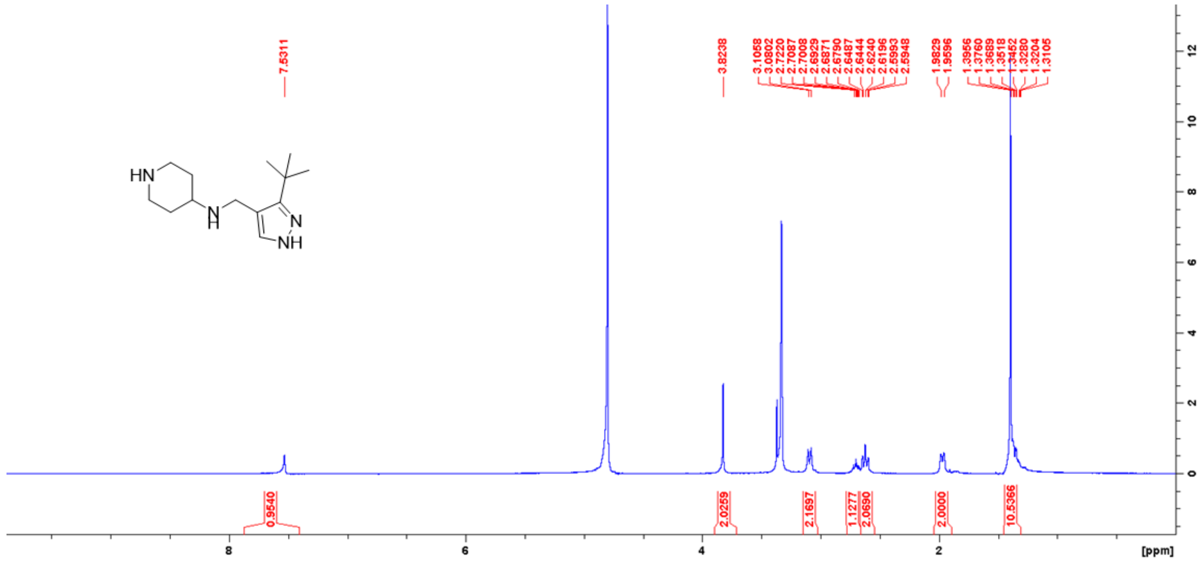

^{1}H and ^{13}C NMR spectra of compound 1 in MeOD

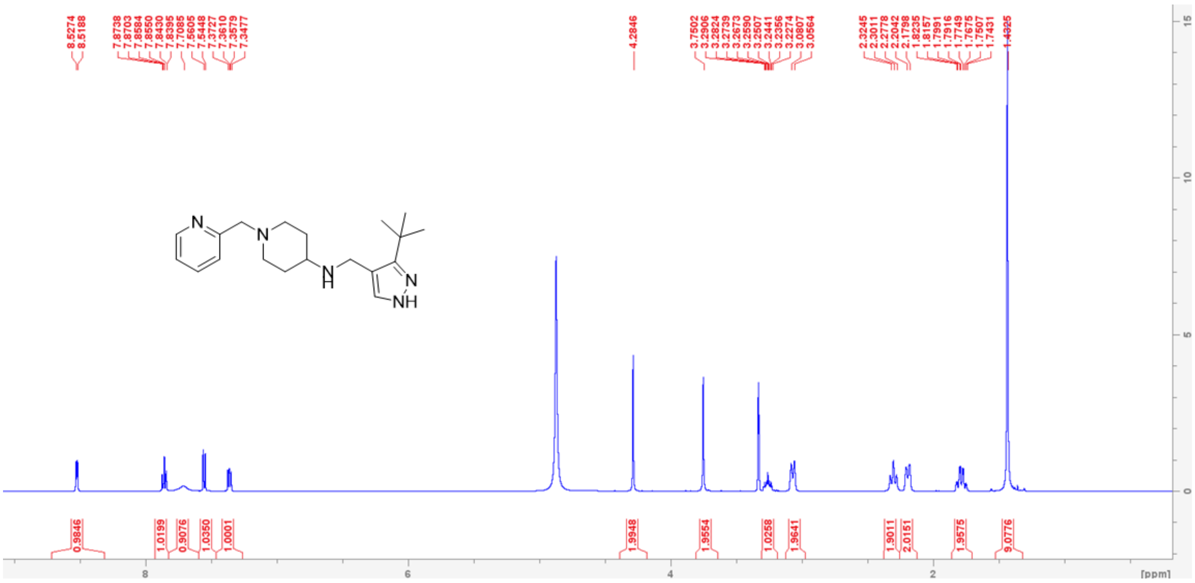

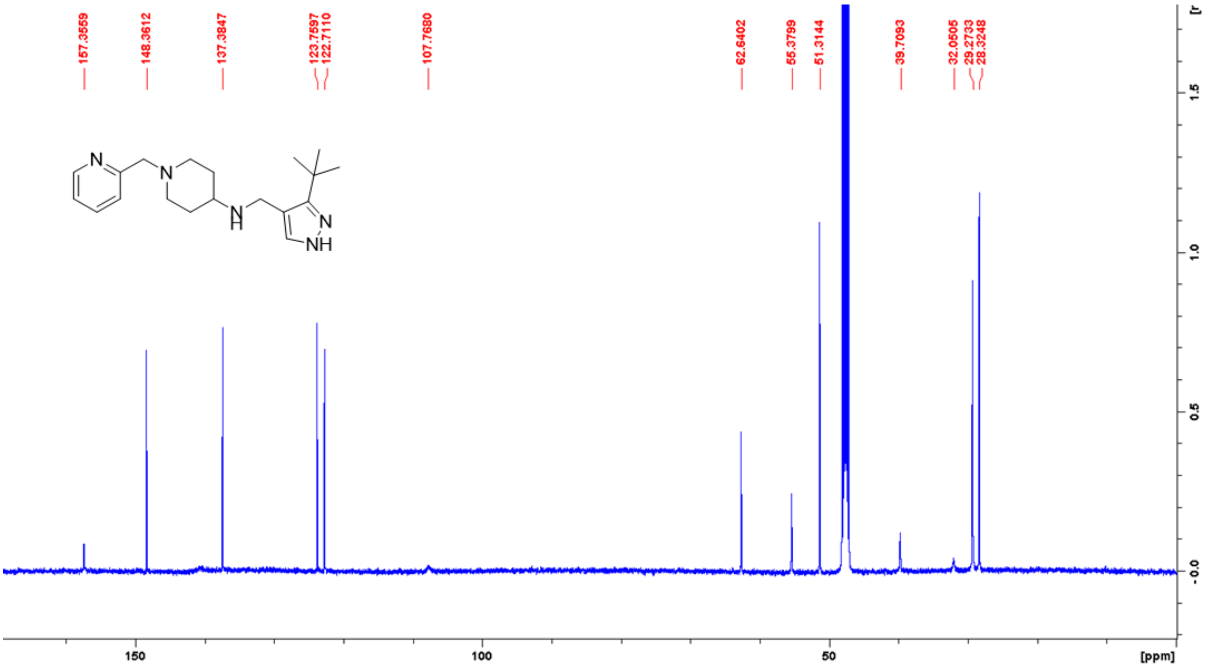

^{1}H and ^{13}C NMR spectra of compound 2 in MeOD

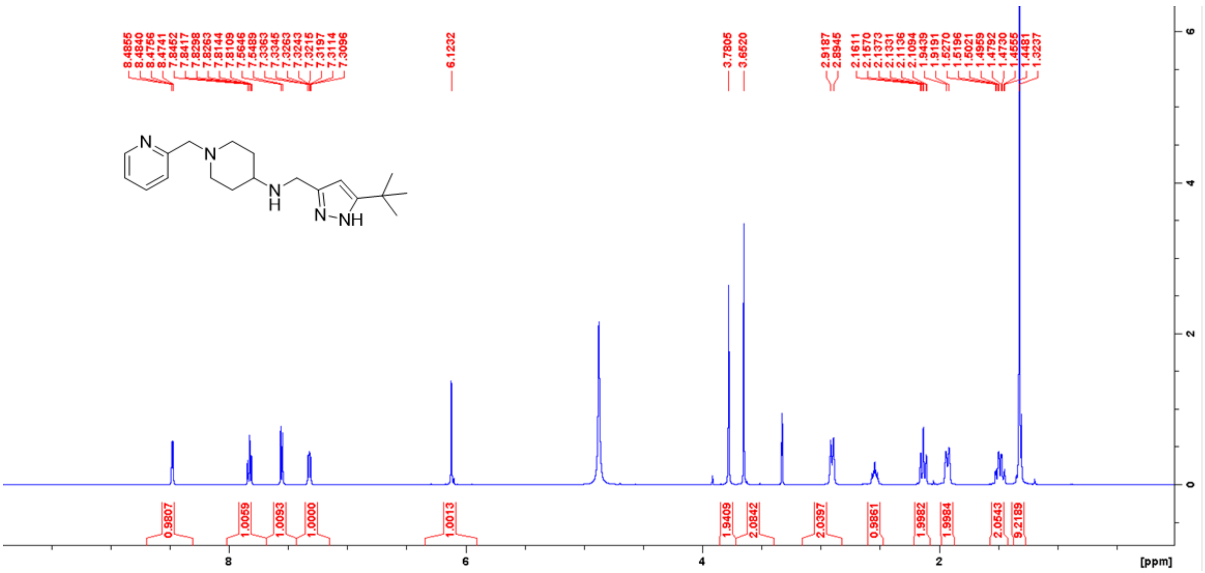

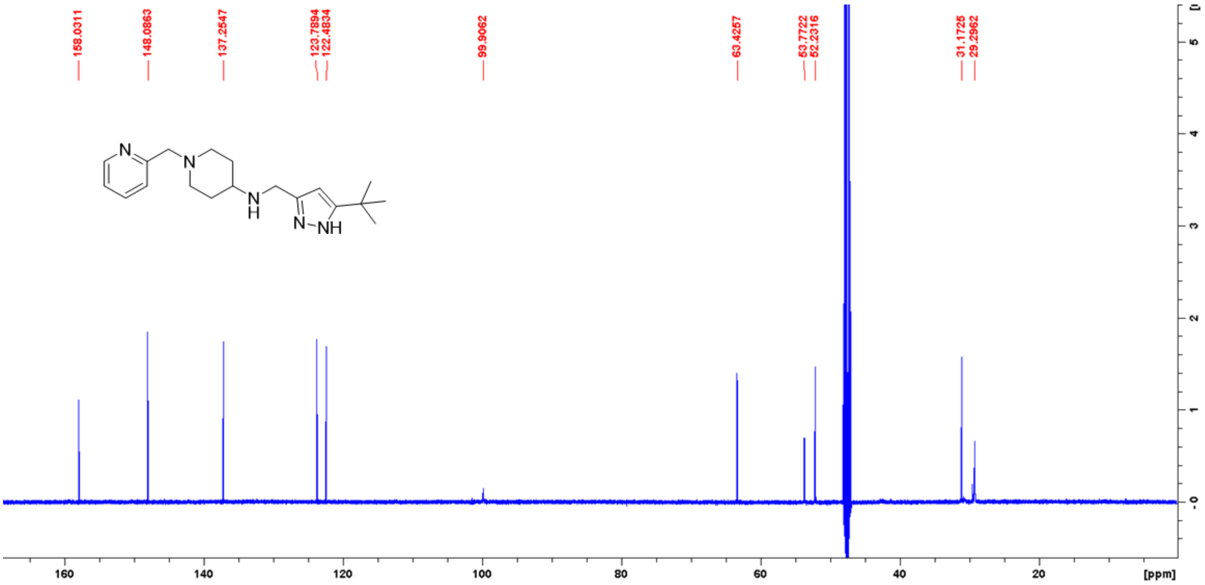

^{1}H and ^{13}C NMR spectra of compound 3 in MeOD

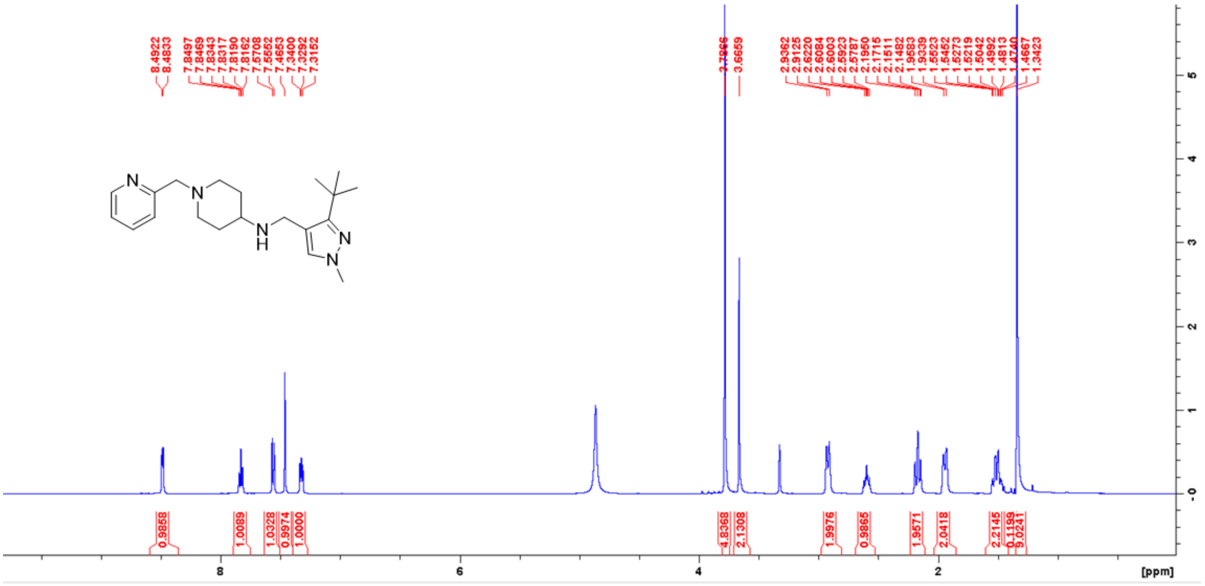

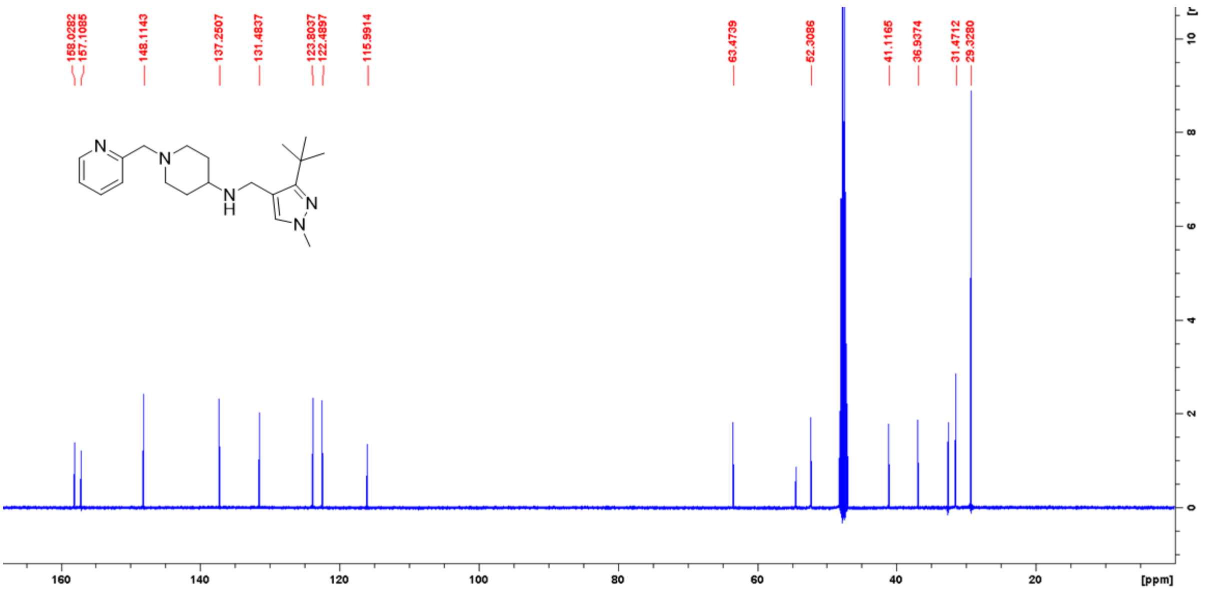

^{1}H and ^{13}C NMR spectra of compound 4 in MeOD

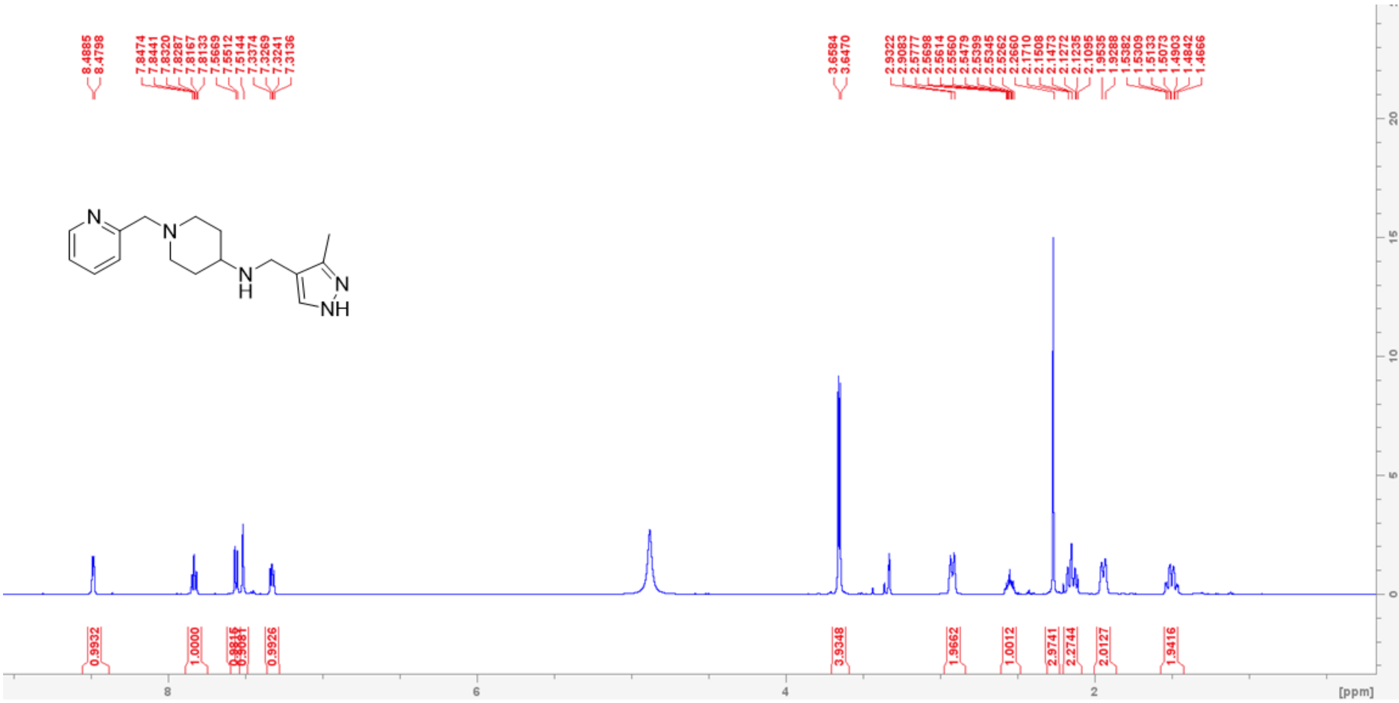

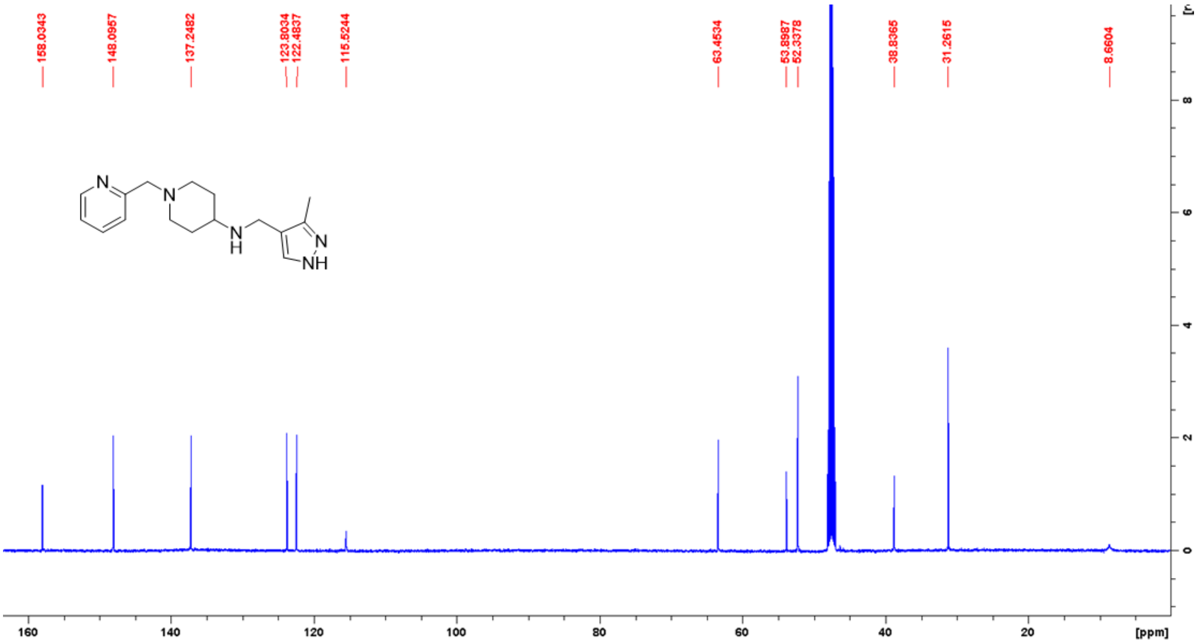

^{1}H and ^{13}C NMR spectra of compound 5 in MeOD

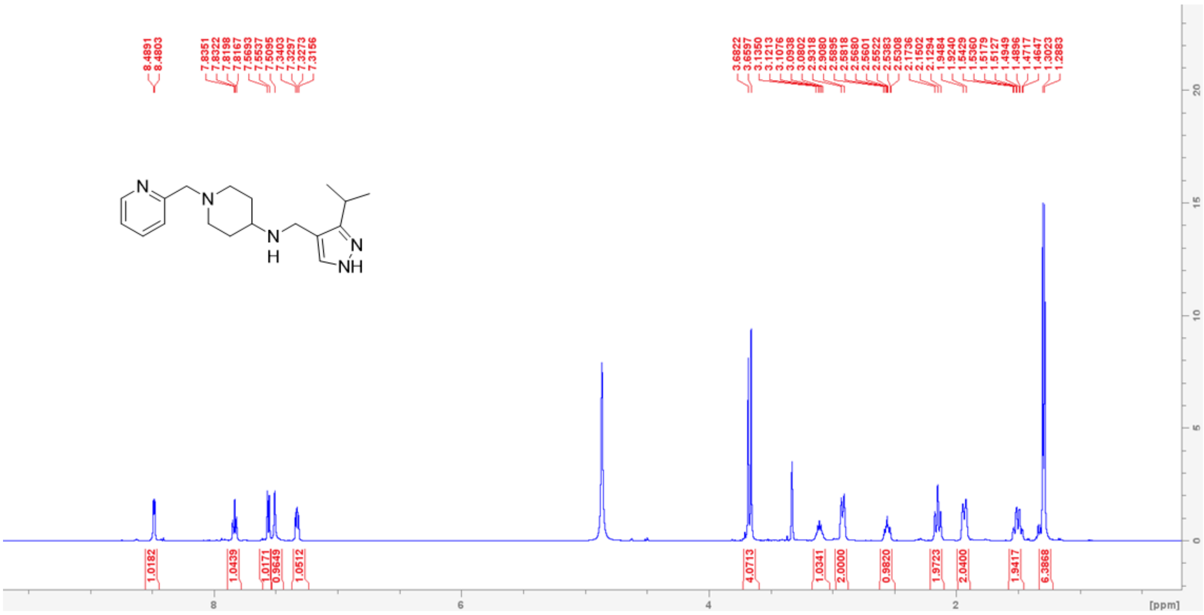

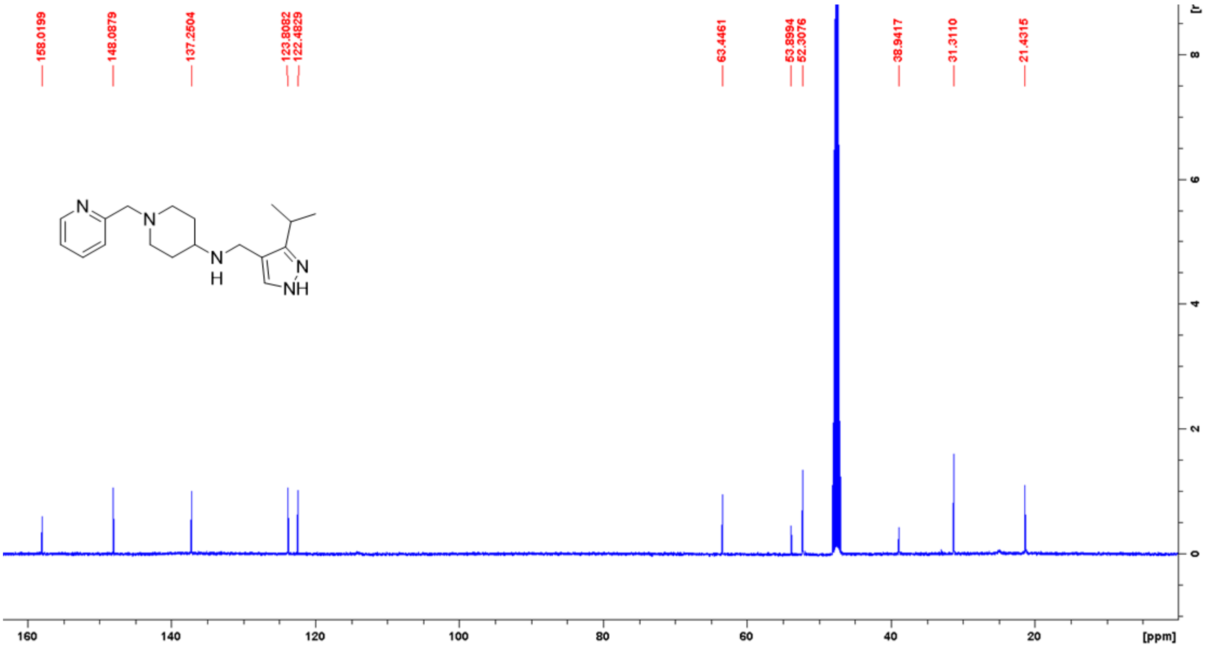

^{1}H and ^{13}C NMR spectra of compound 6 in MeOD

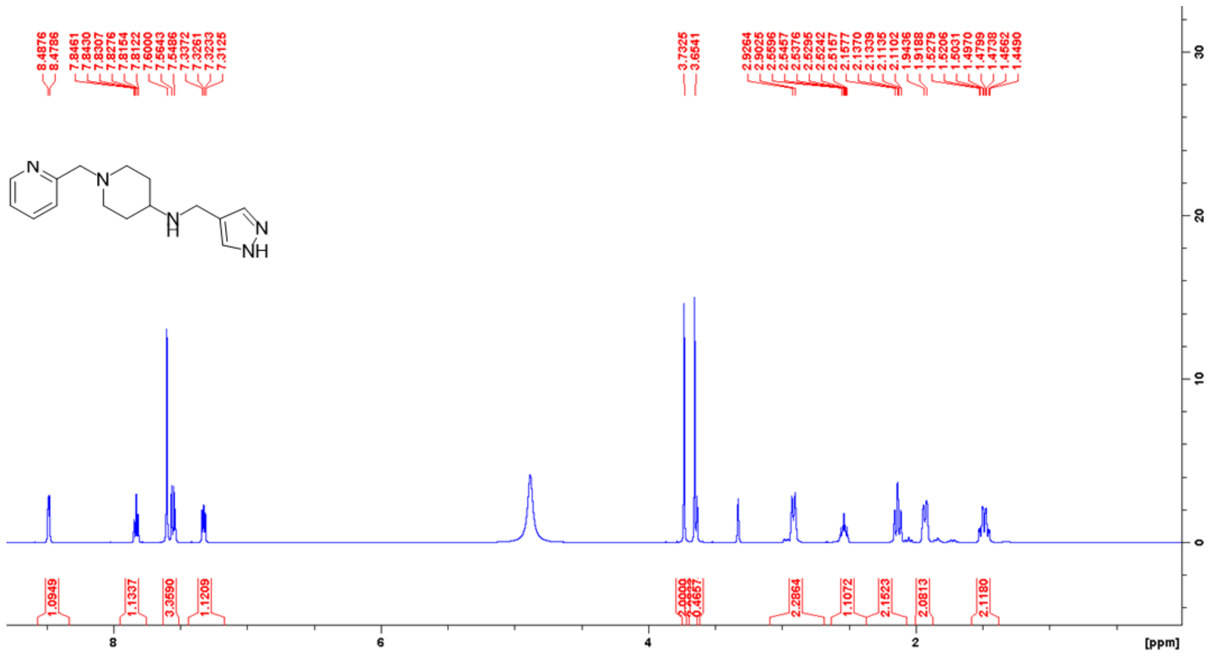

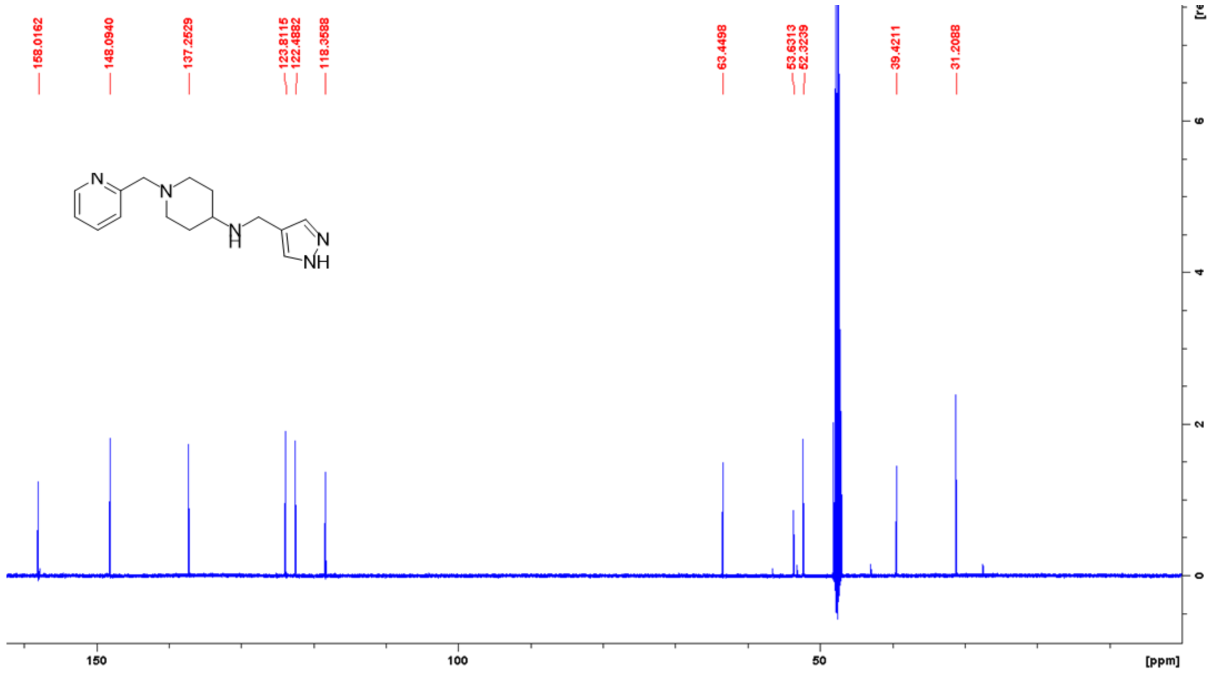

^{1}H and ^{13}C NMR spectra of compound 7 in MeOD

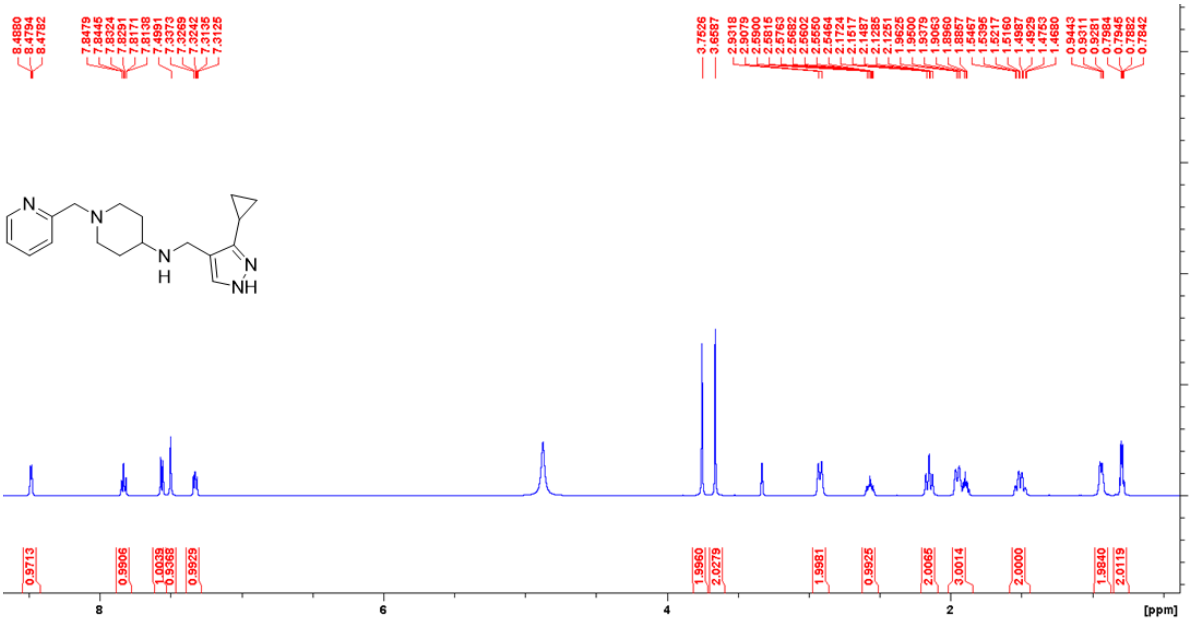

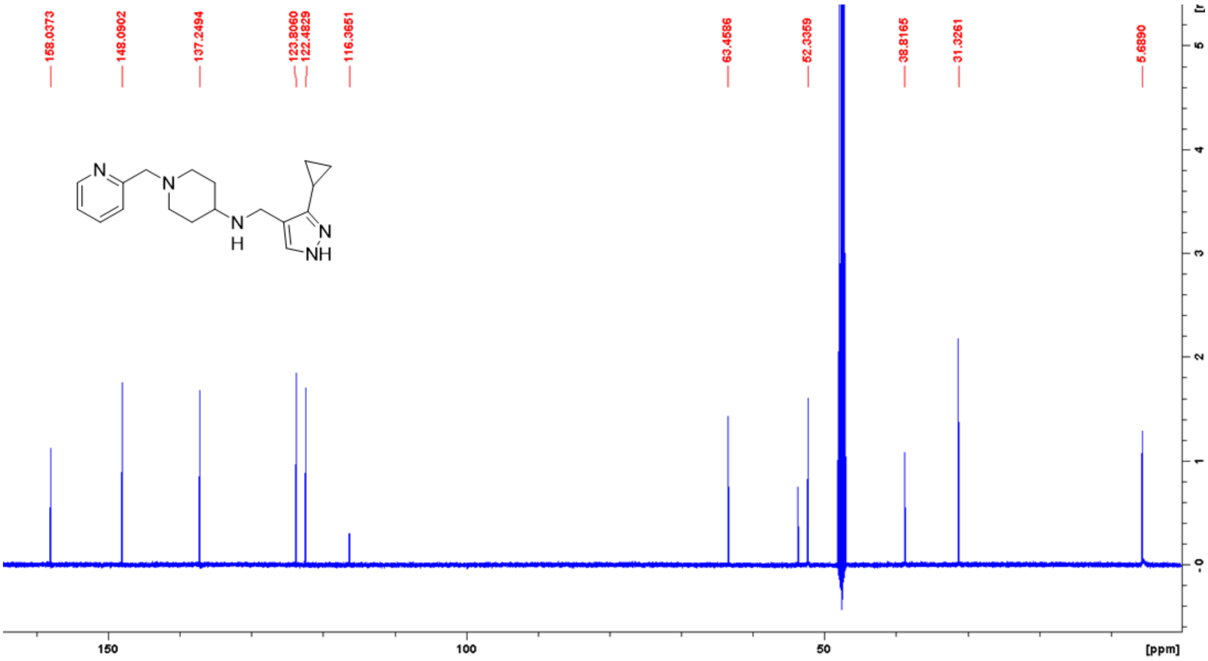

^{1}H and ^{13}C NMR spectra of compound 8 in MeOD

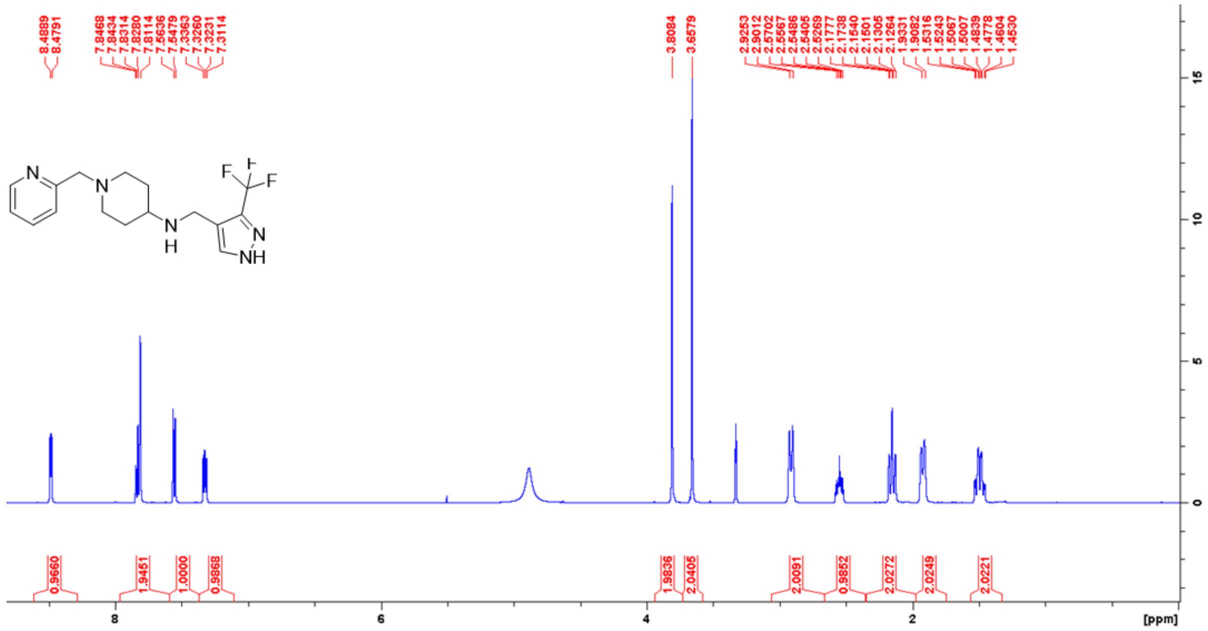

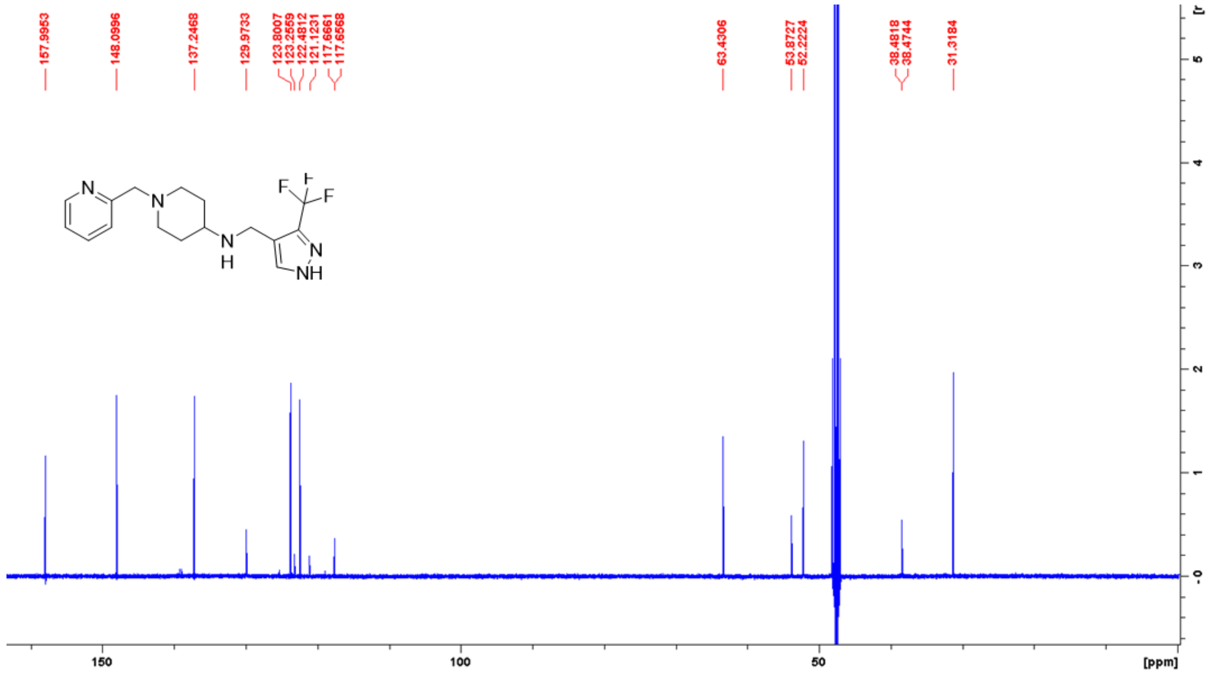

^{1}H and ^{13}C NMR spectra of compound 9 in MeOD

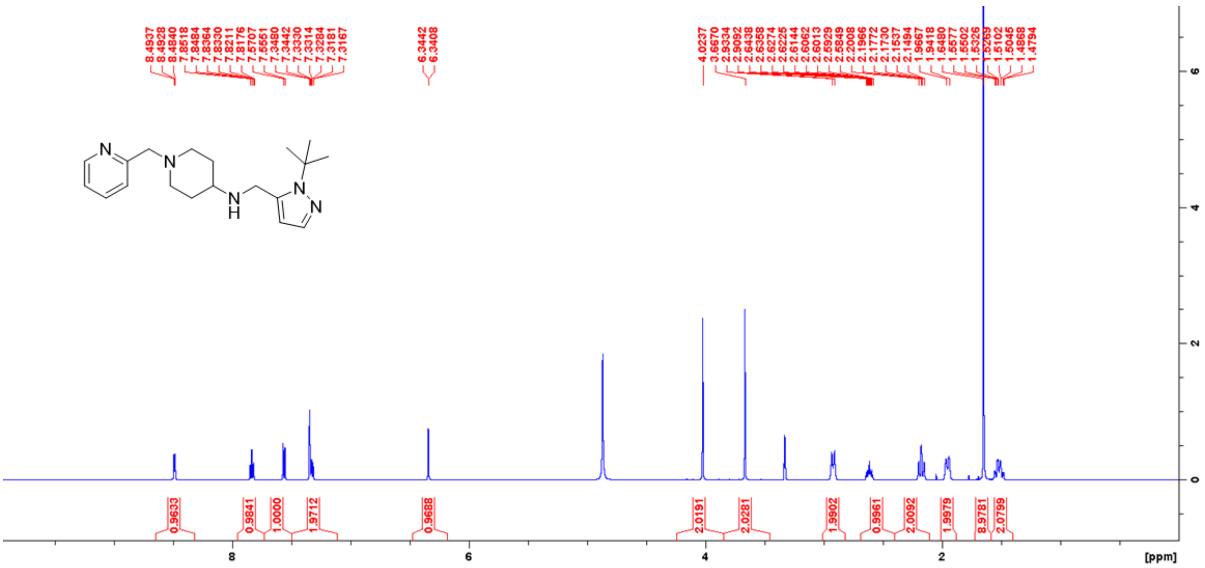

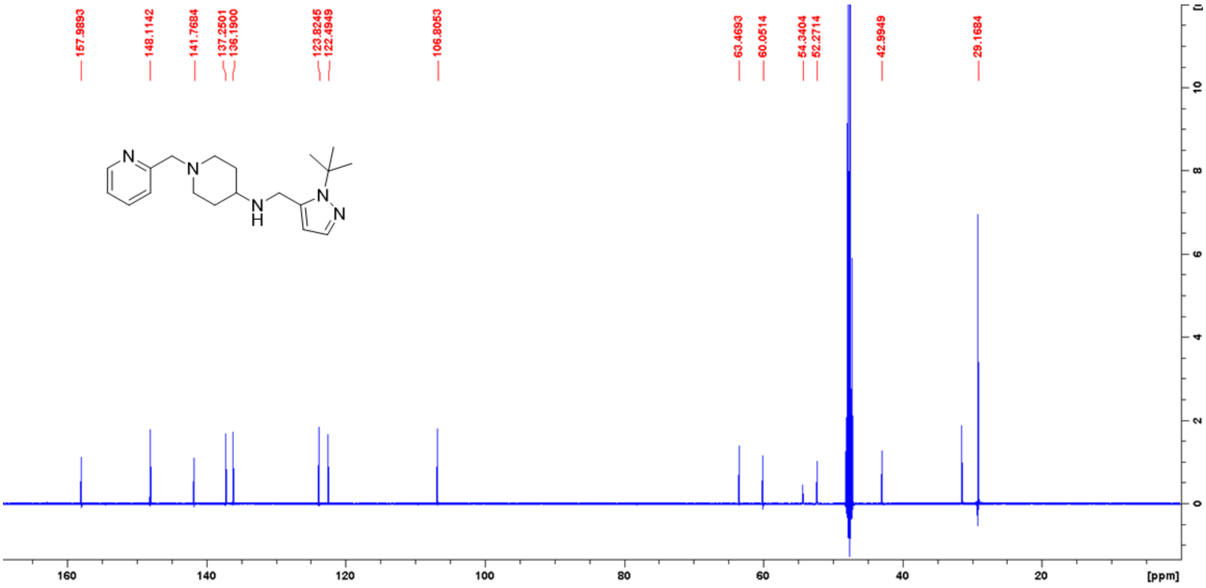

^{1}H and ^{13}C NMR spectra of compound 10 in MeOD

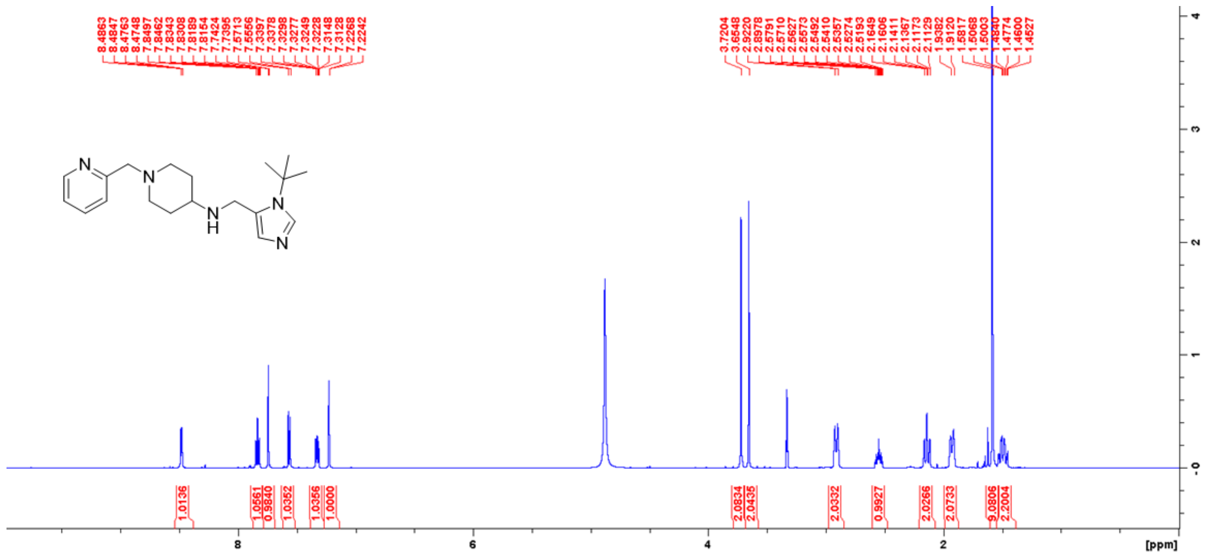

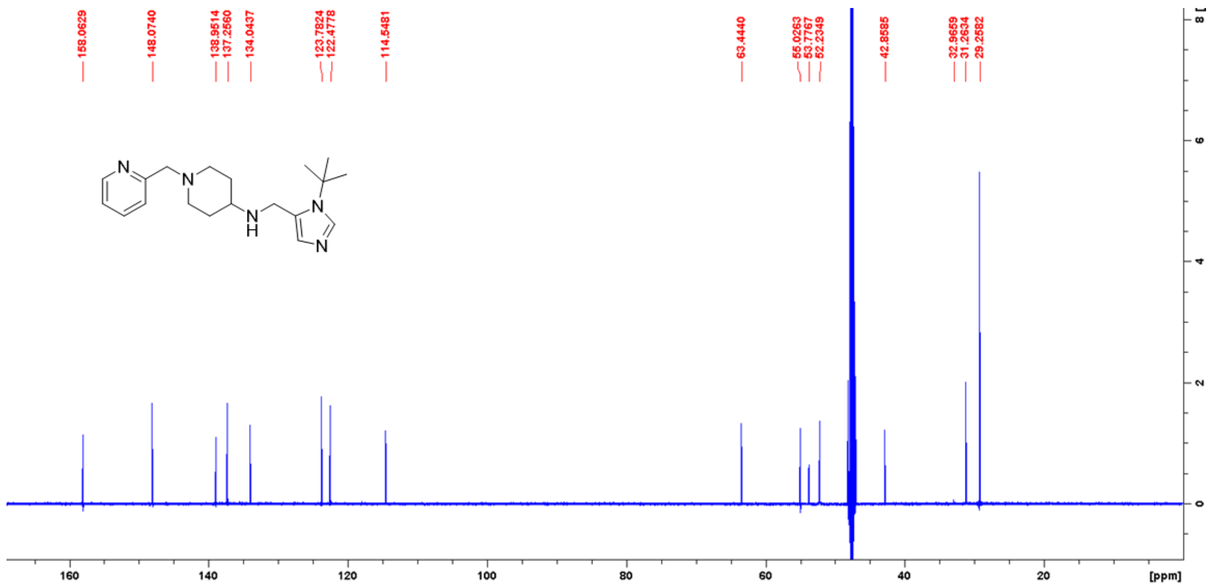

^{1}H and ^{13}C NMR spectra of compound 11 in MeOD

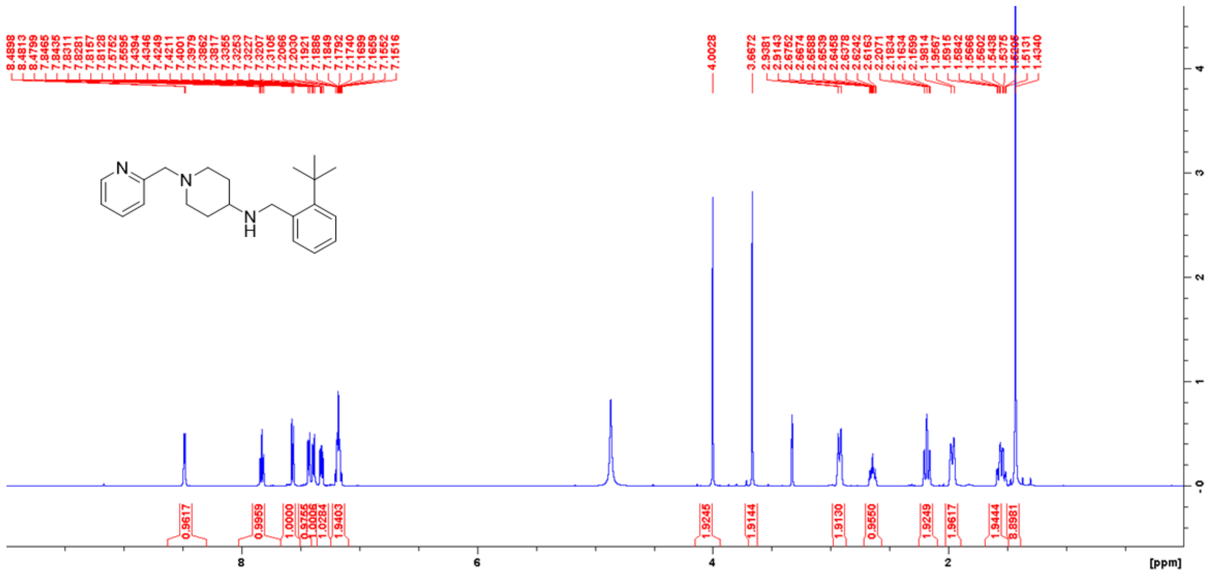

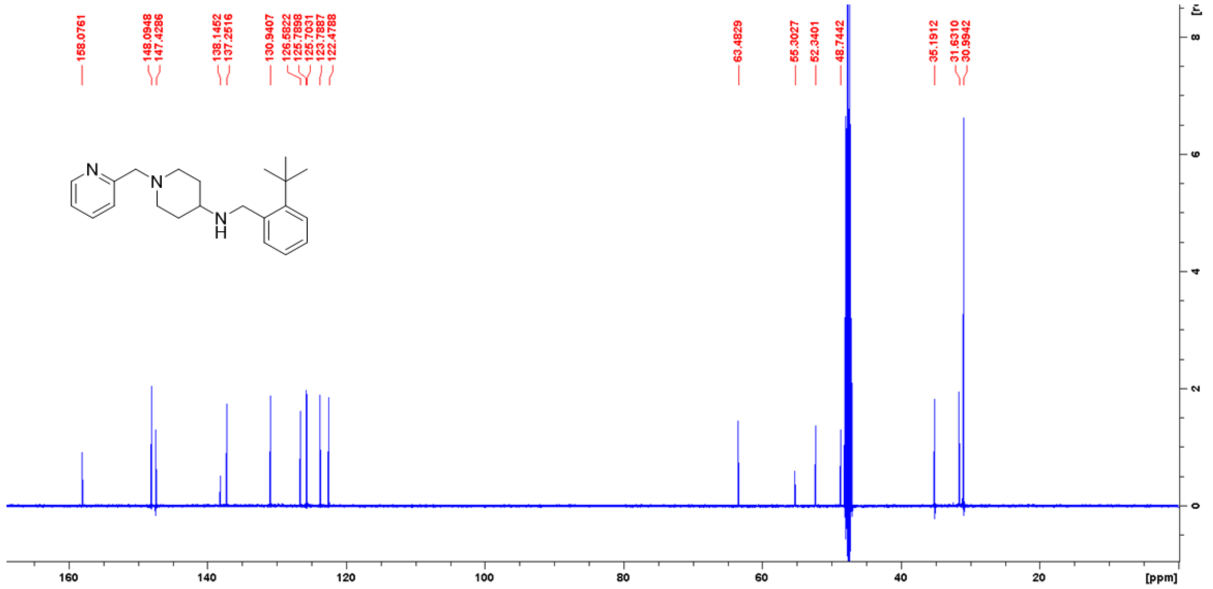

^{1}H and ^{13}C NMR spectra of compound 12 in MeOD

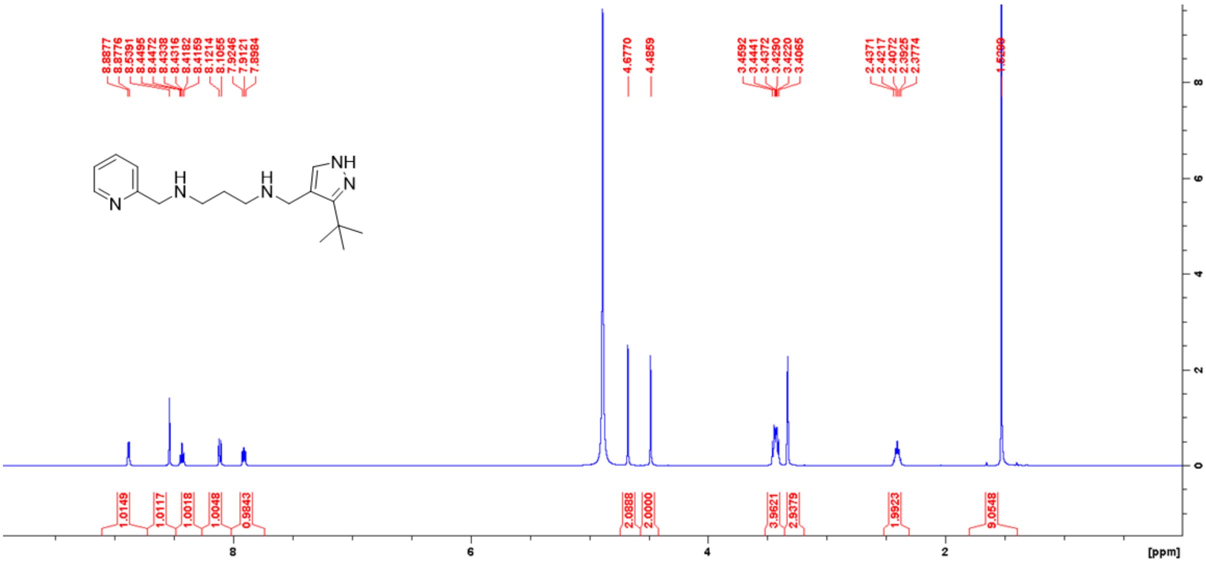

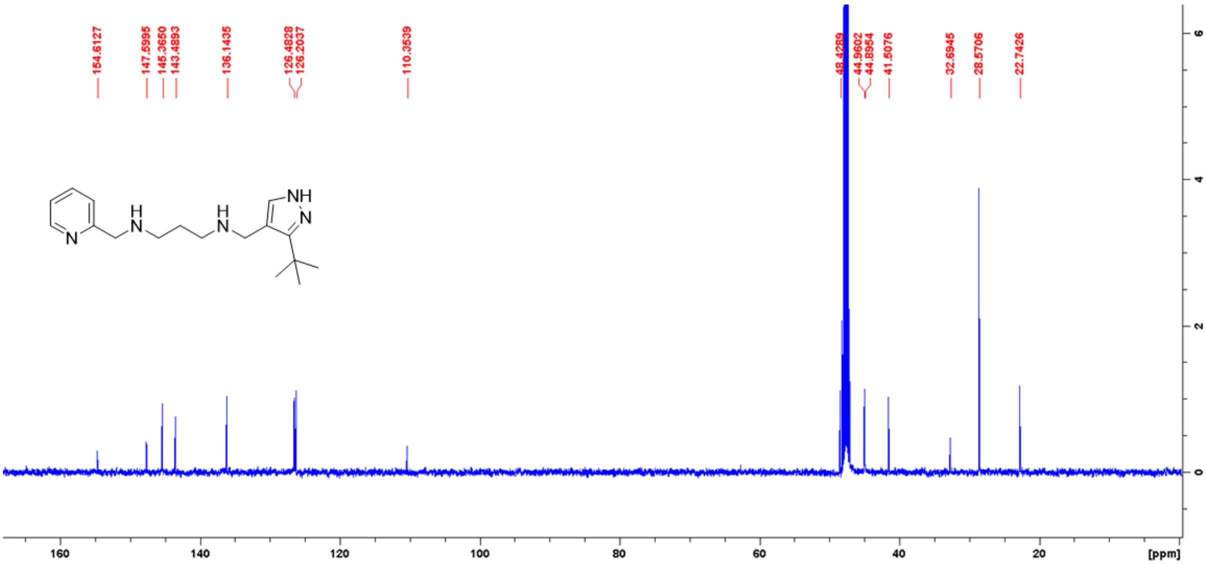

^1H and ^{13}C NMR spectra of compound 13 in MeOD

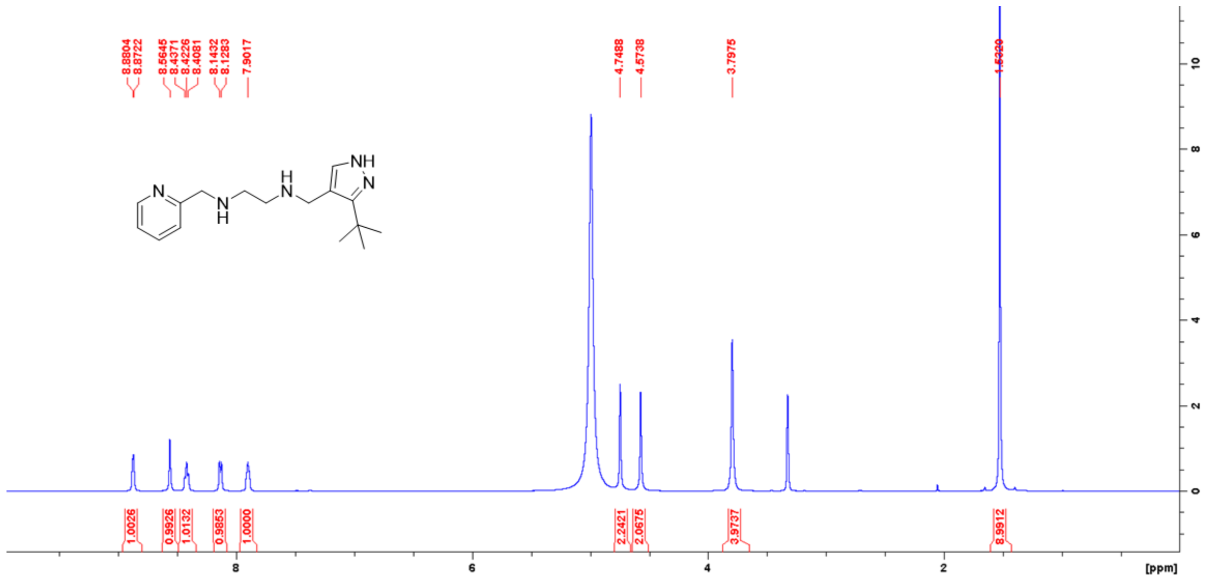

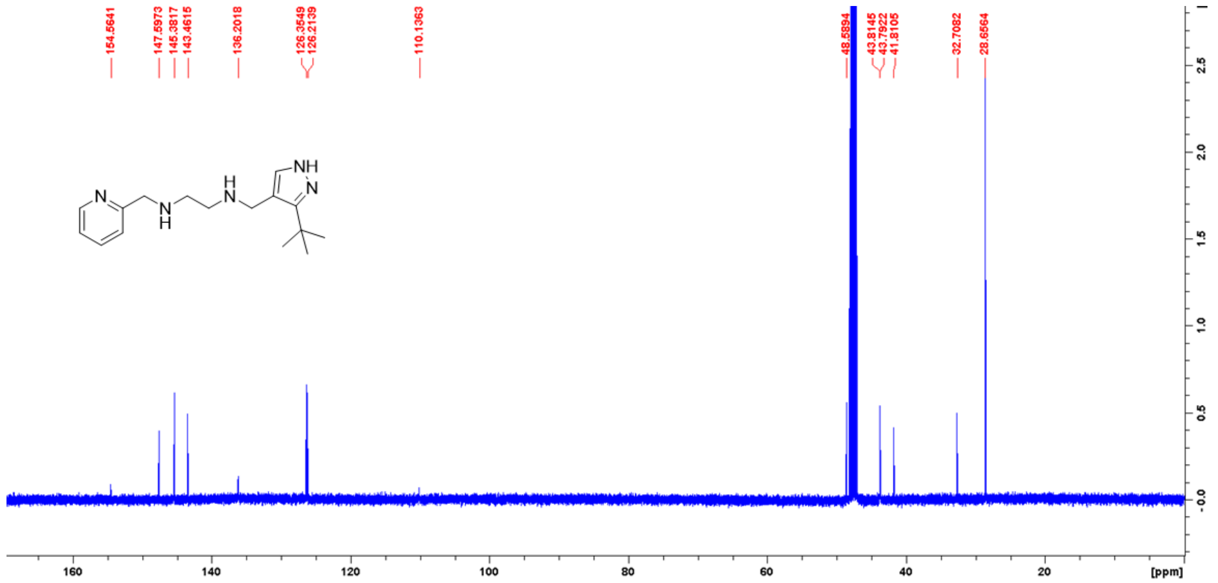

^{1}H and ^{13}C NMR spectra of compound 14 in MeOD

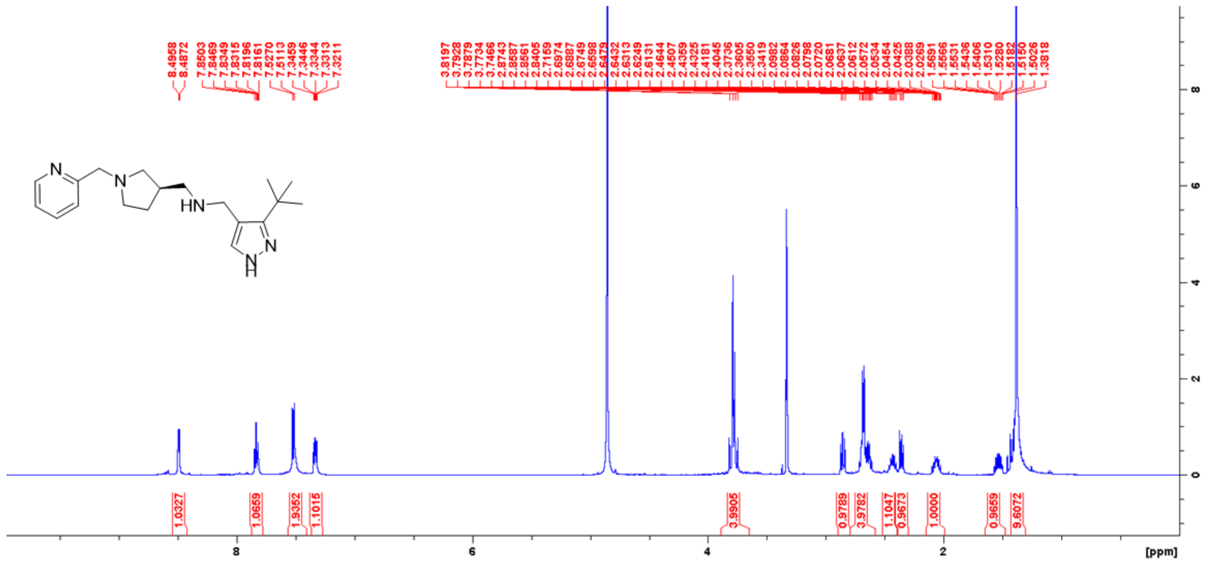

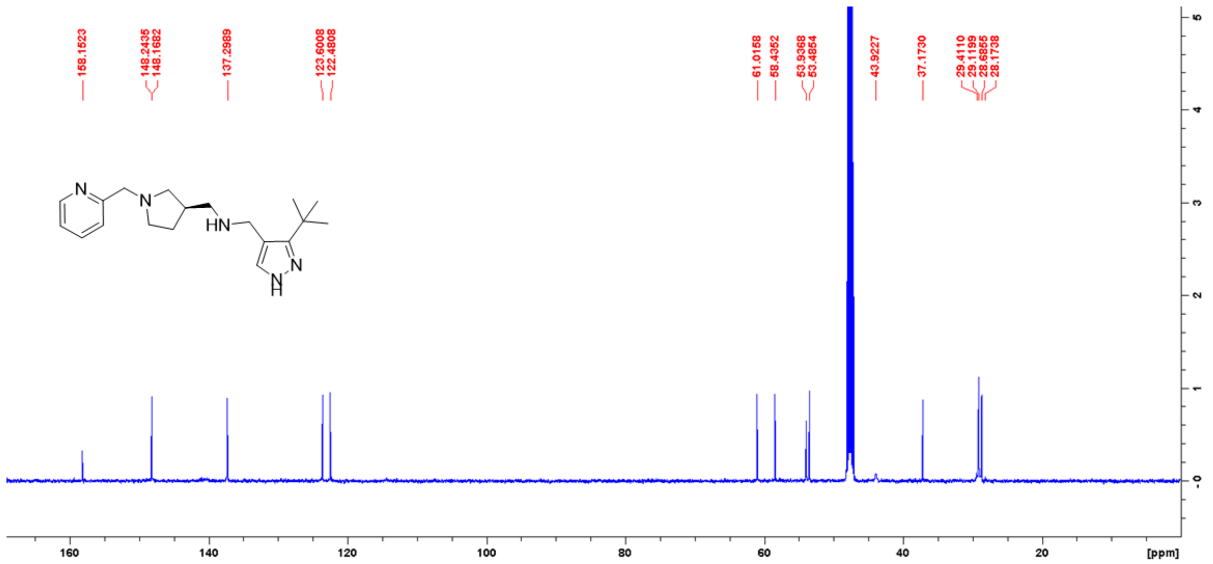

^{1}H and ^{13}C NMR spectra of compound 15 in MeOD

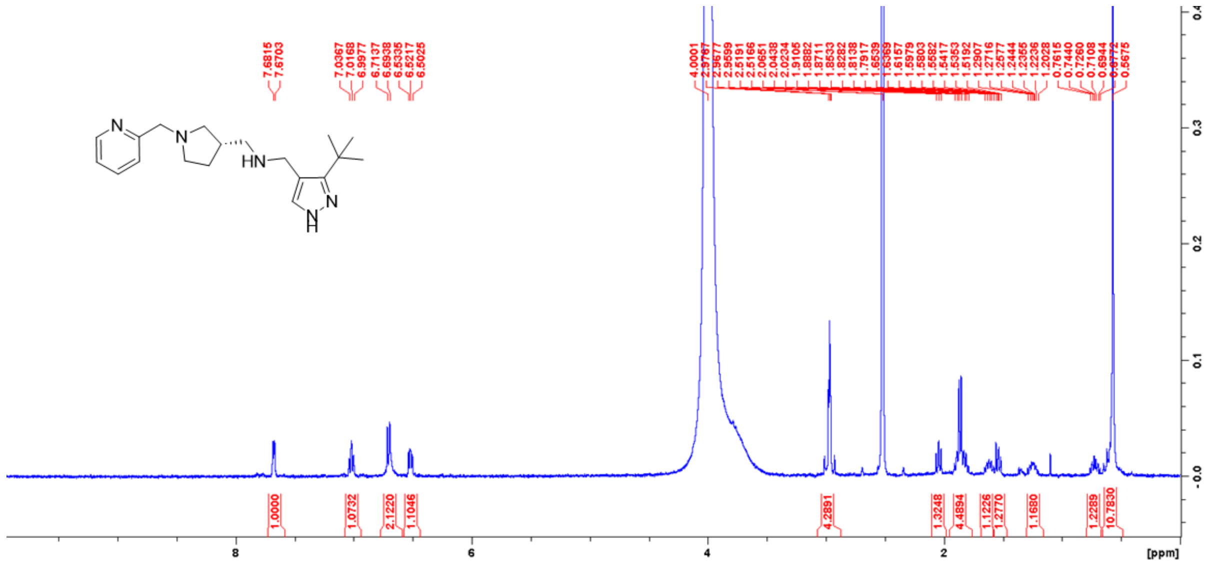

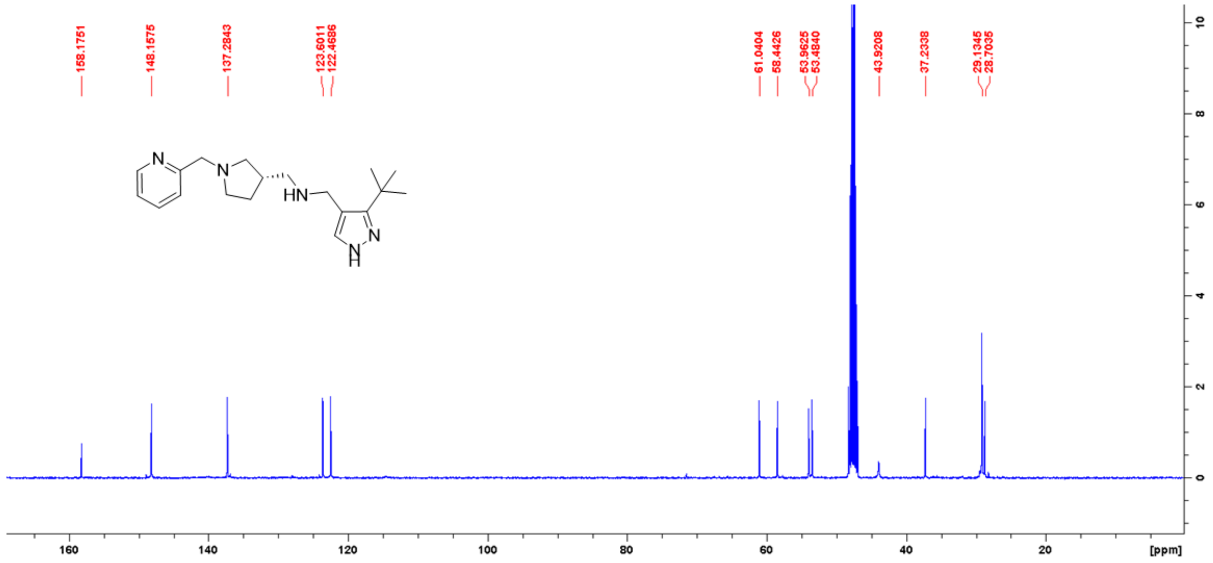

^{1}H and ^{13}C NMR spectra of compound 16 in MeOD

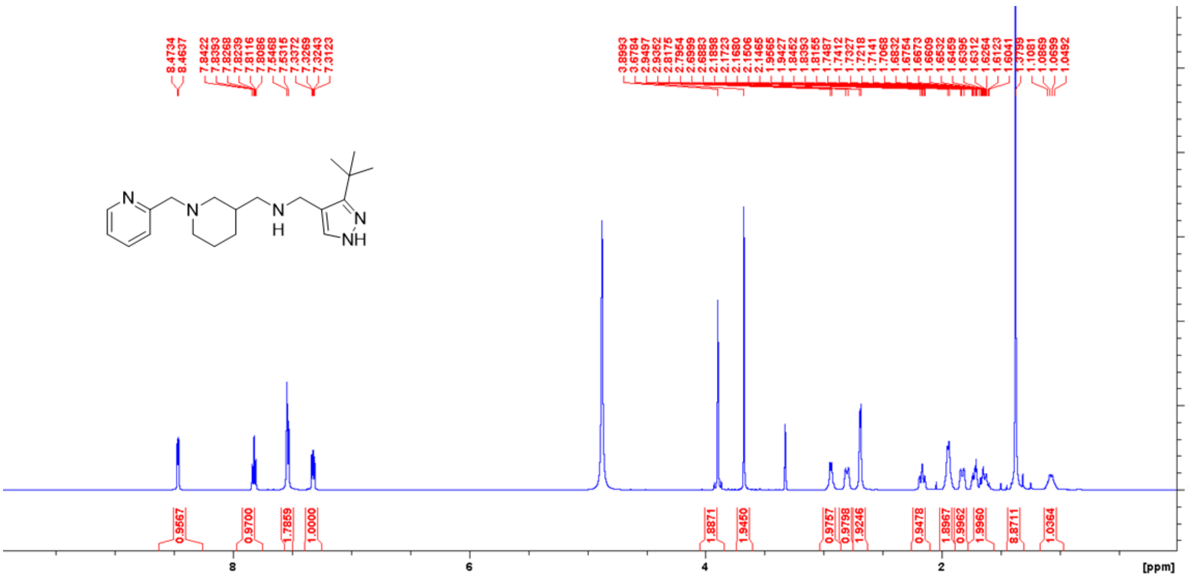

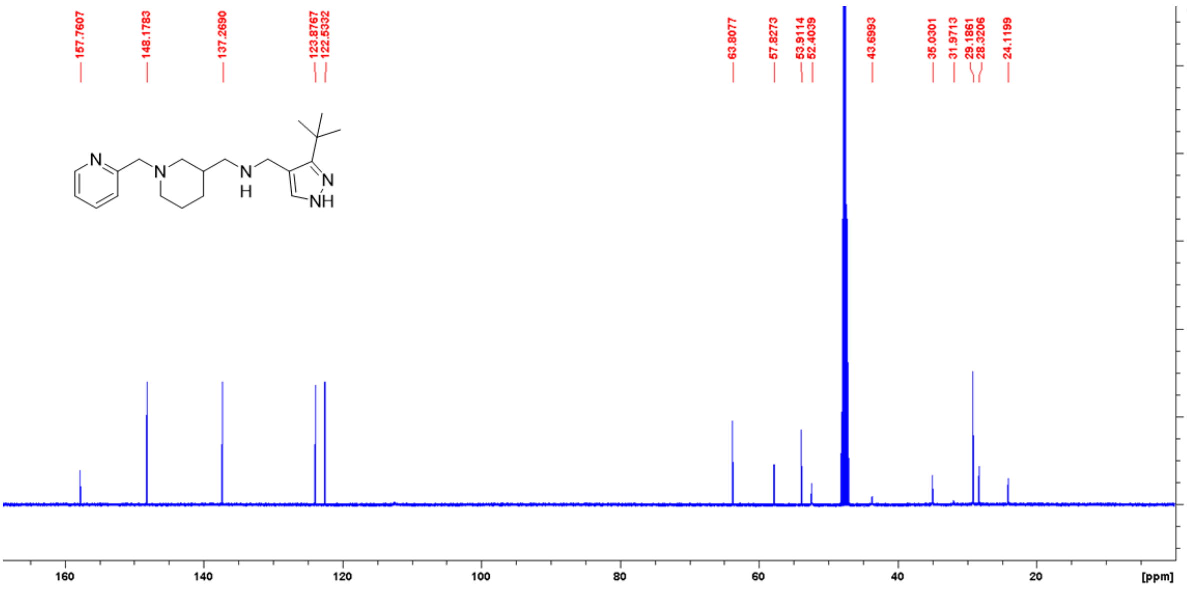

^{1}H and ^{13}C NMR spectra of compound 17 in MeOD

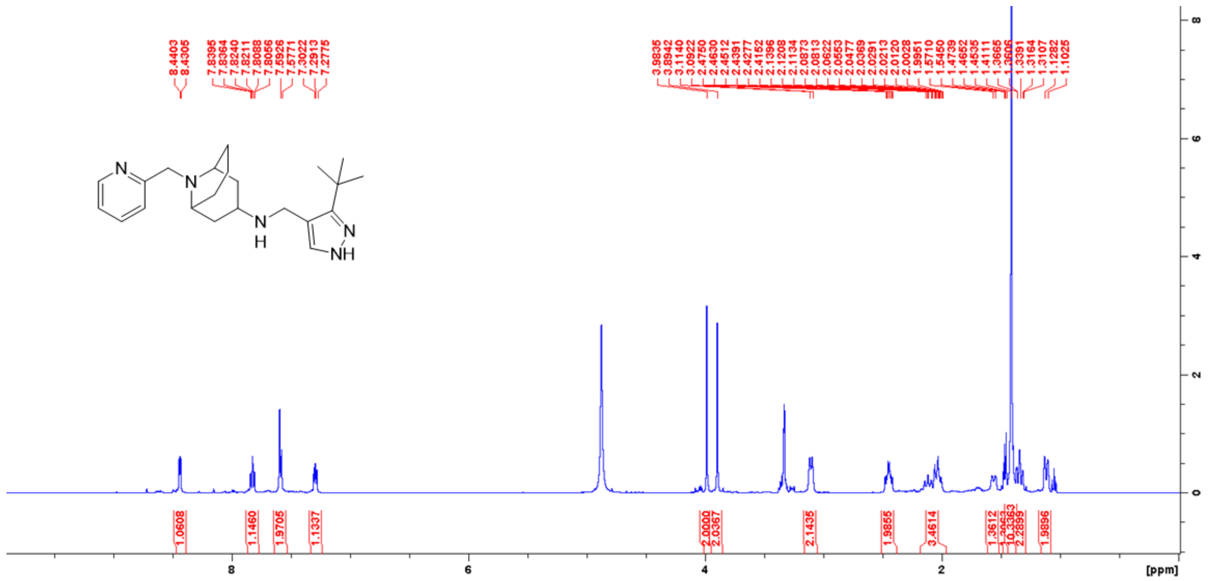

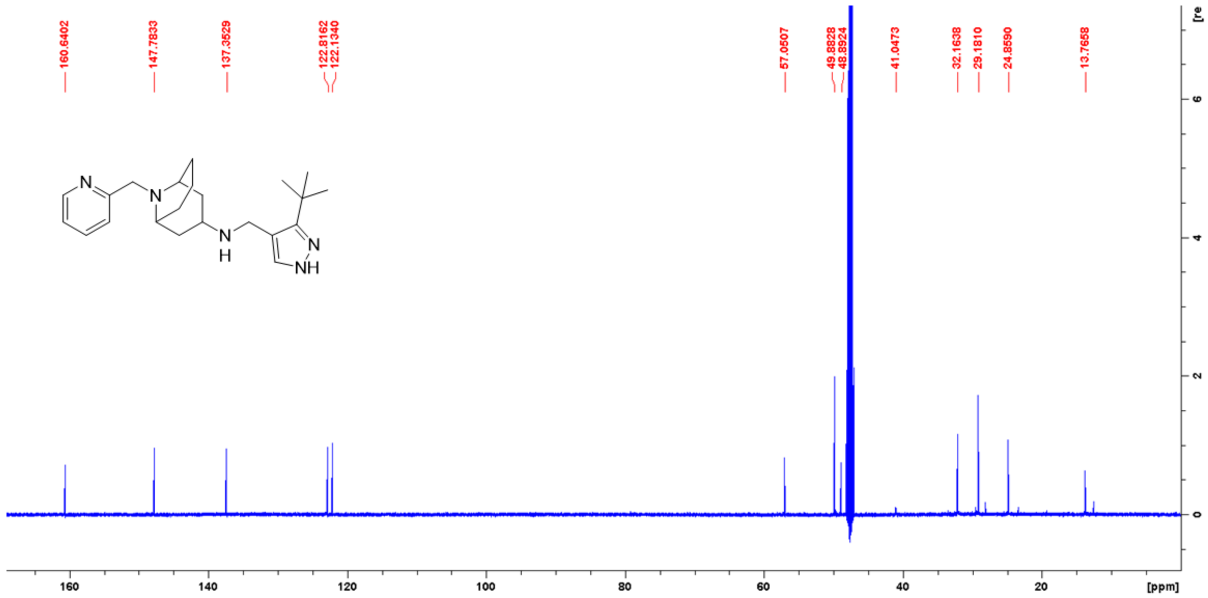

^{1}H and ^{13}C NMR spectra of compound 18 in MeOD

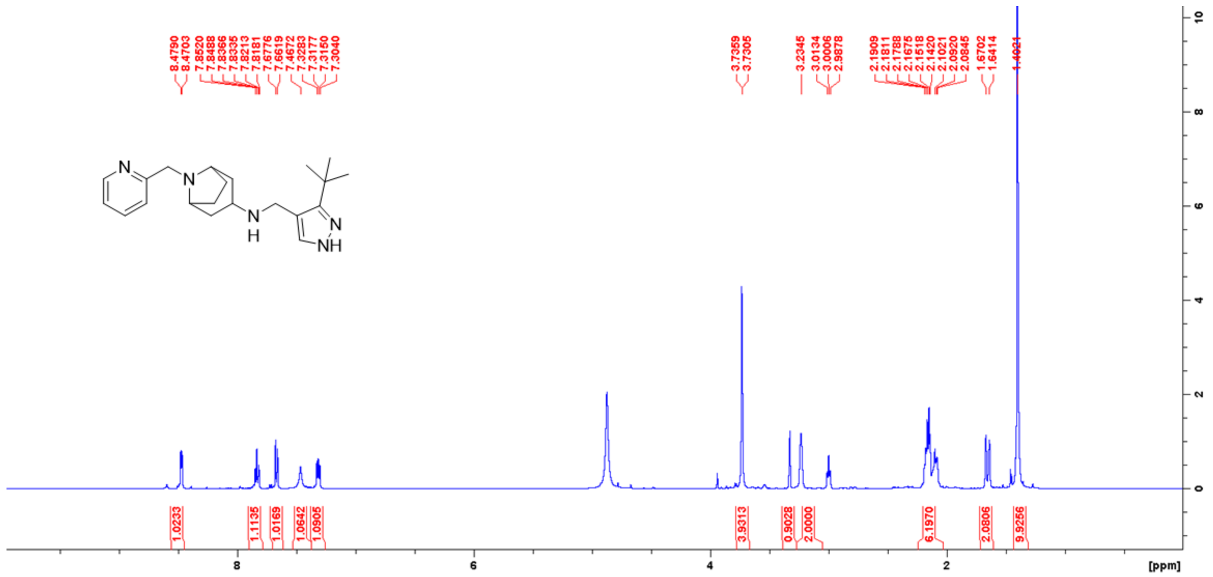

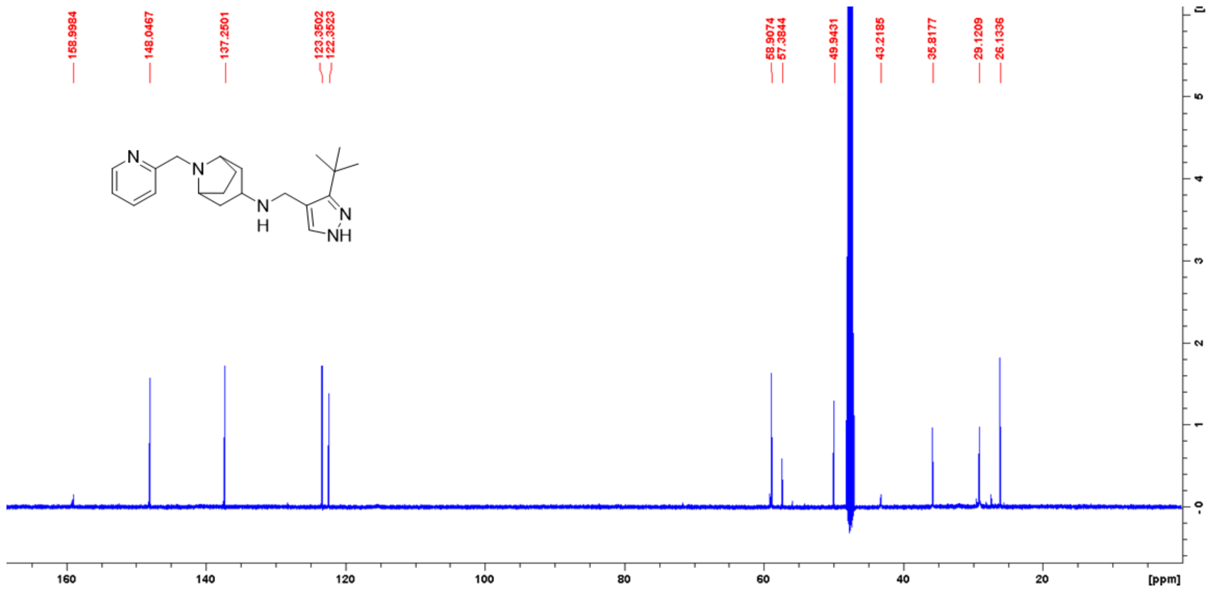

^{1}H and ^{13}C NMR spectra of compound 19 in MeOD

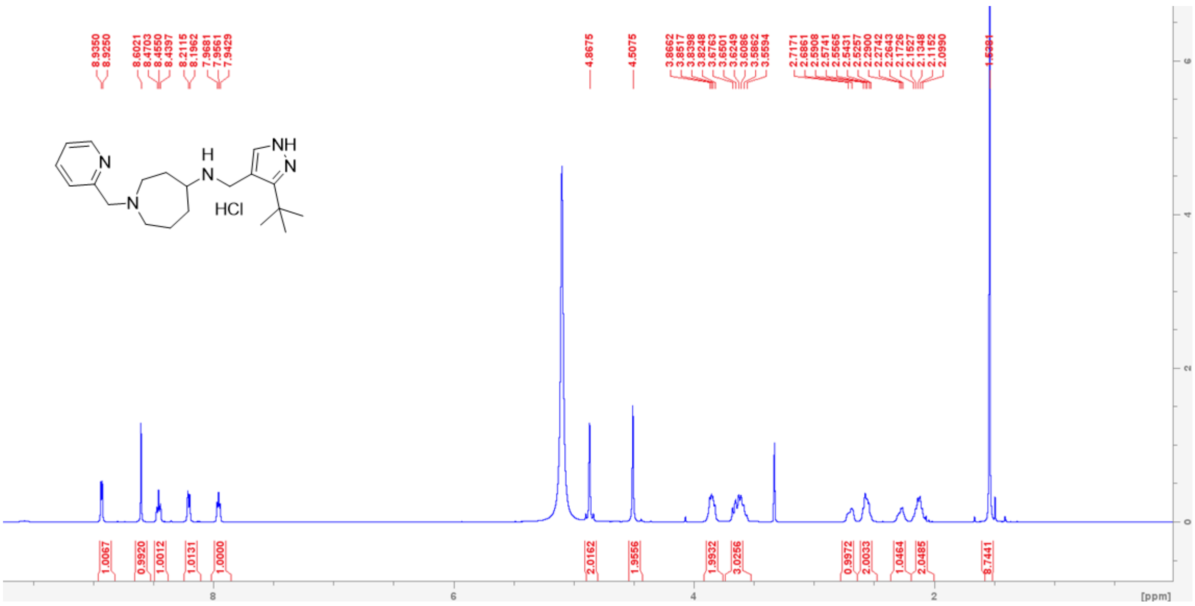

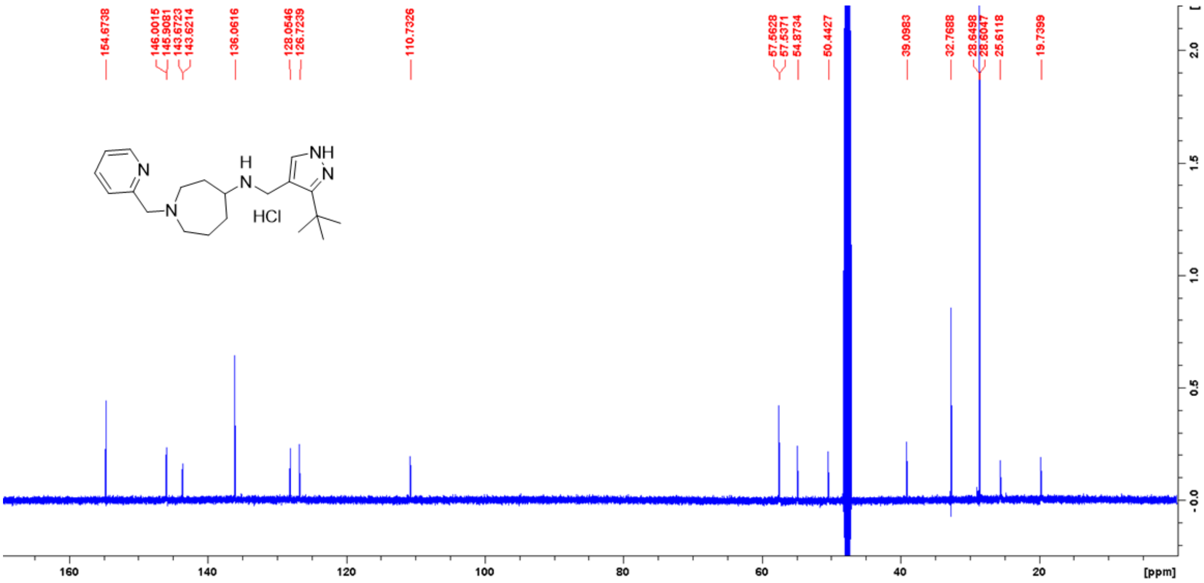

^{1}H NMR spectra of compound 20 in MeOD

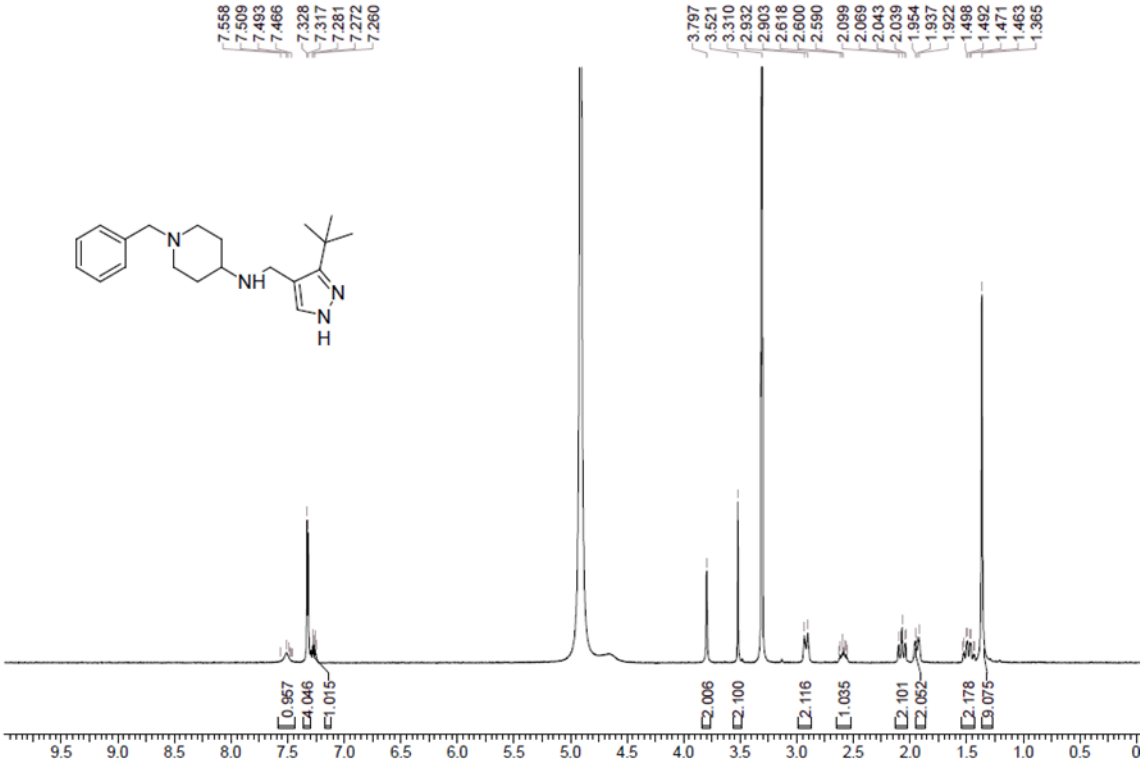

^{1}H and ^{13}C NMR spectra of compound 21 in MeOD

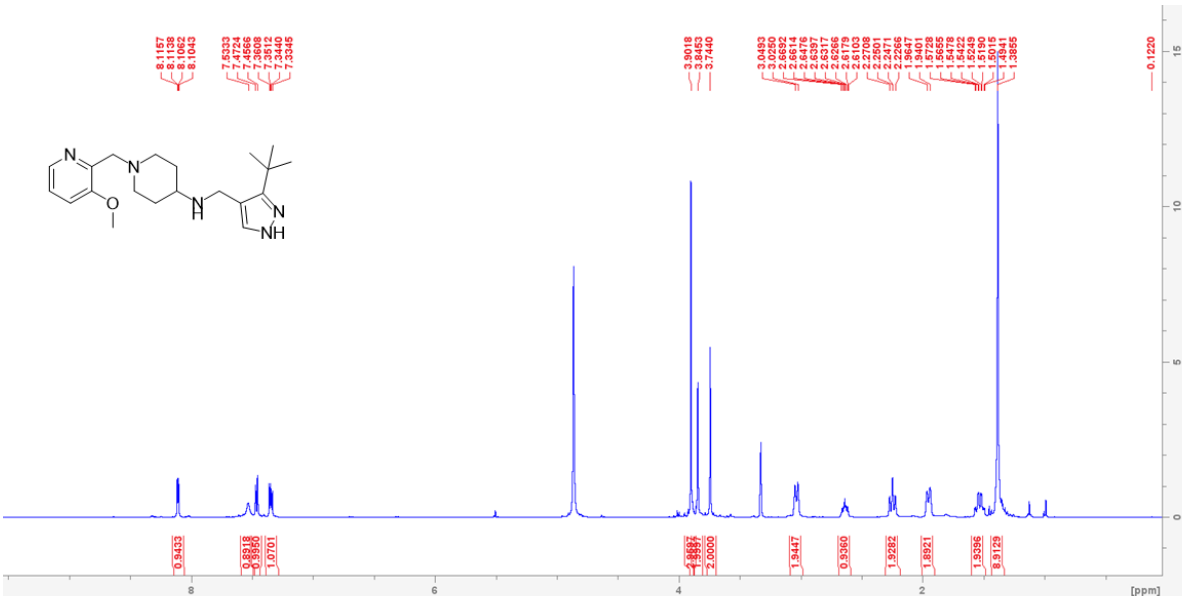

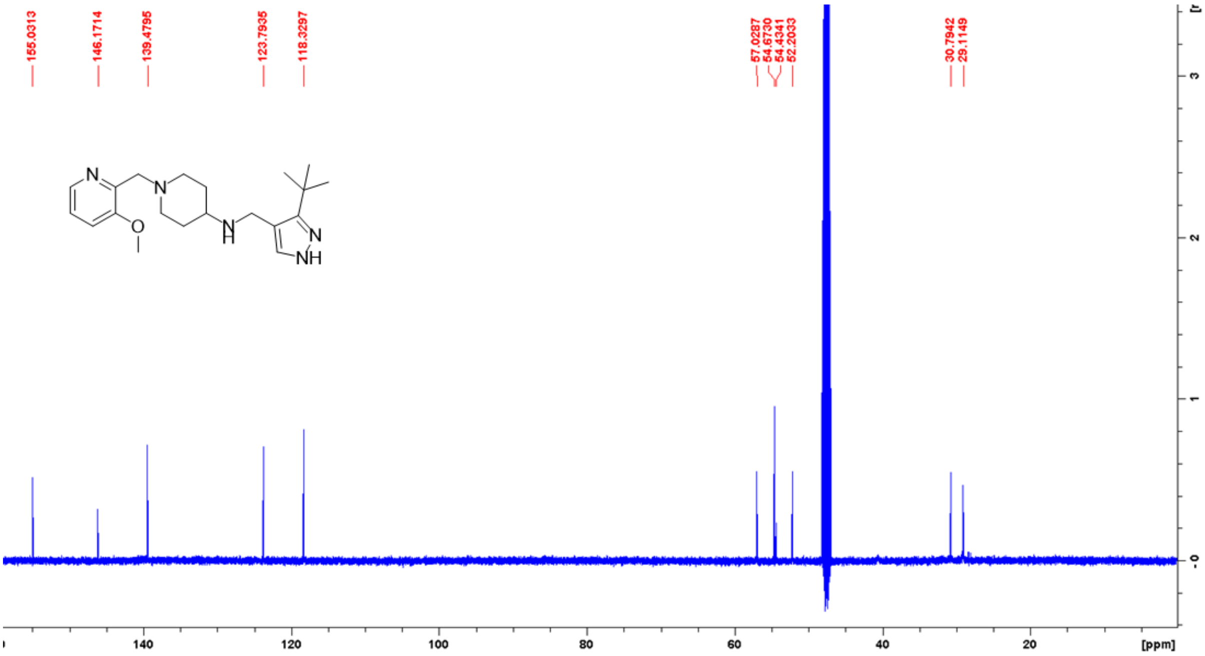

^{1}H NMR spectra of compound 22 in DMSO-d6

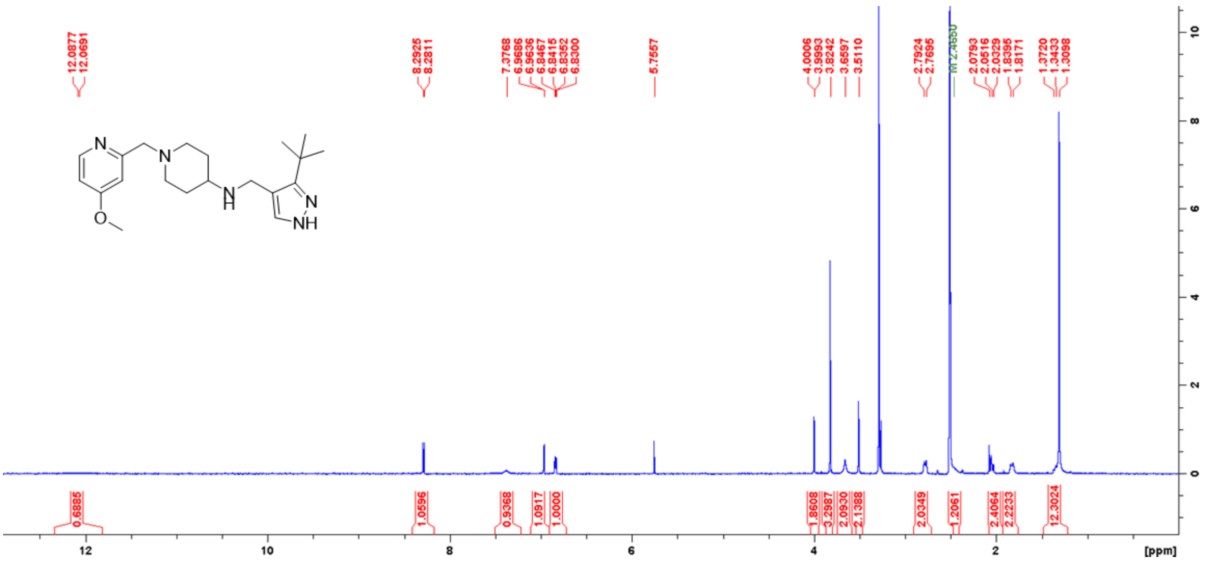

^{1}H and ^{13}C NMR spectra of compound 23 in MeOD

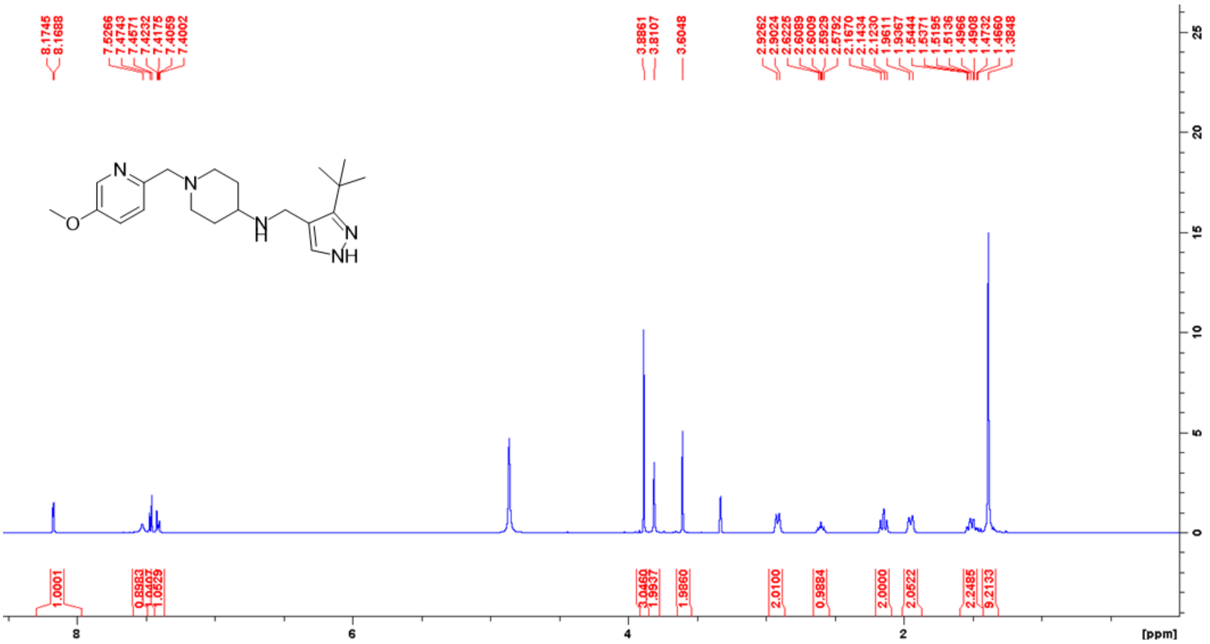

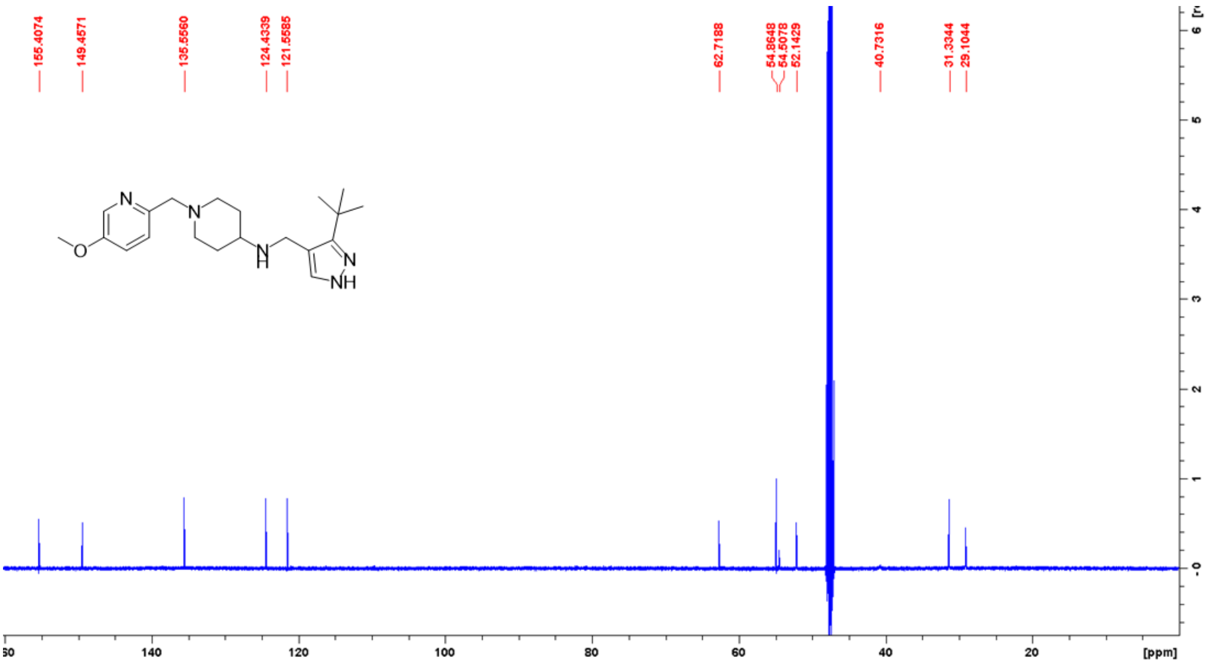

^{1}H and ^{13}C NMR spectra of compound 24 in MeOD

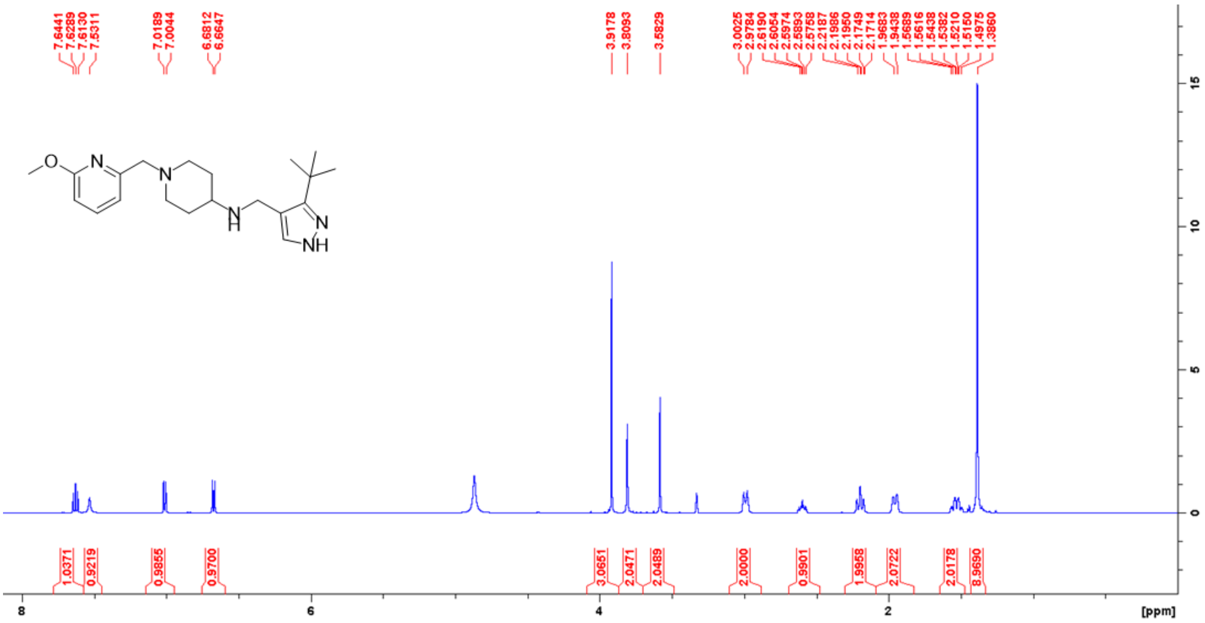

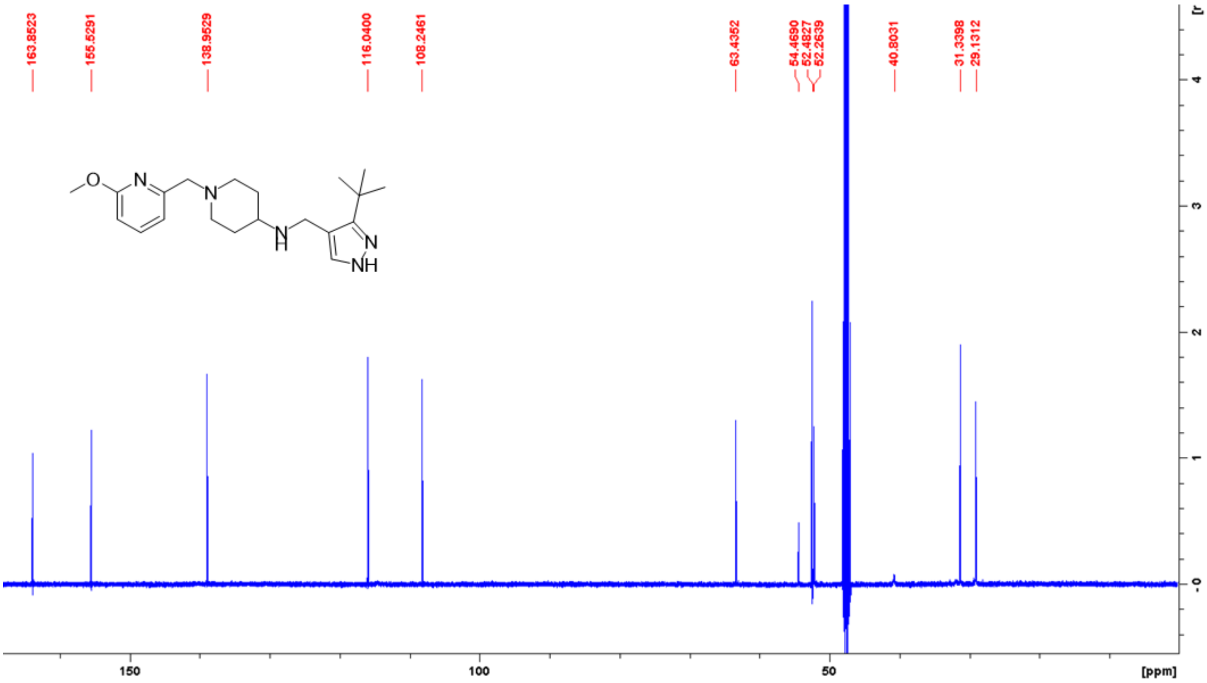

^{1}H and ^{13}C NMR spectra of compound 25 in MeOD

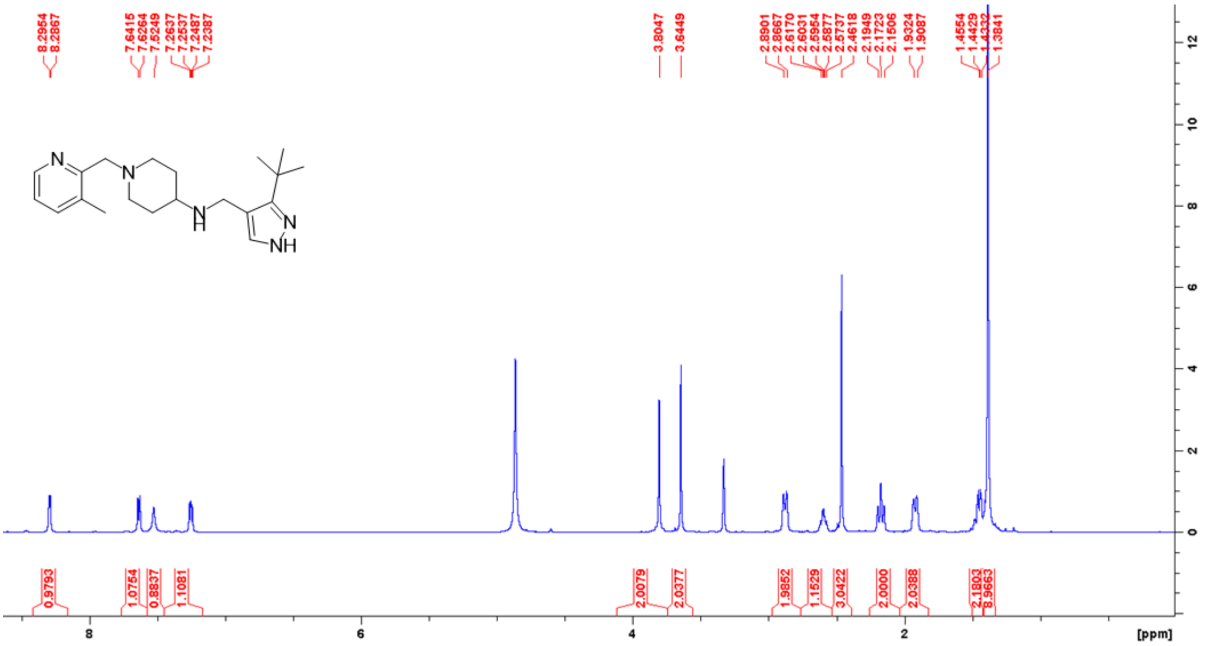

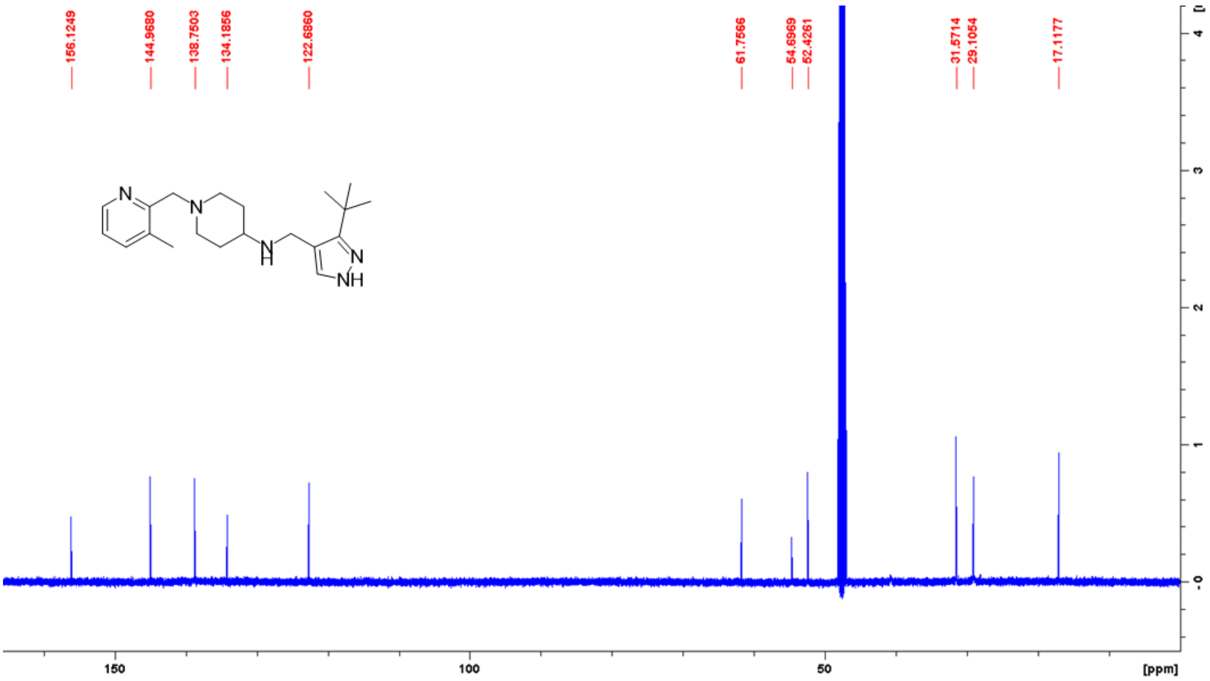

^{1}H NMR spectra of compound 26 in DMSO-d6

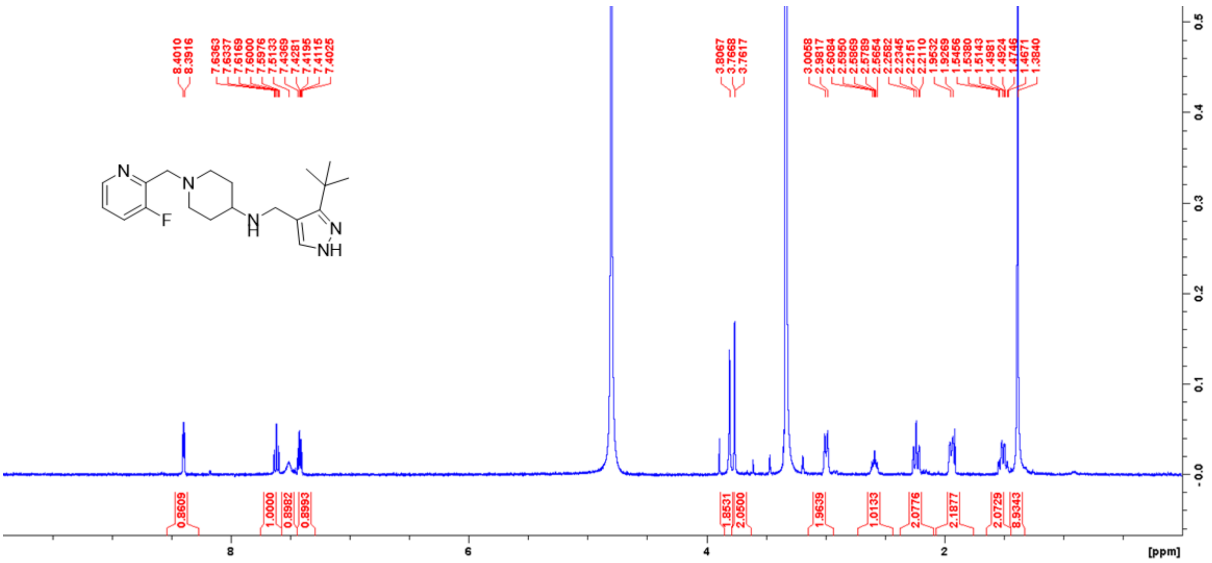

^{1}H and ^{13}C NMR spectra of compound 27 in MeOD

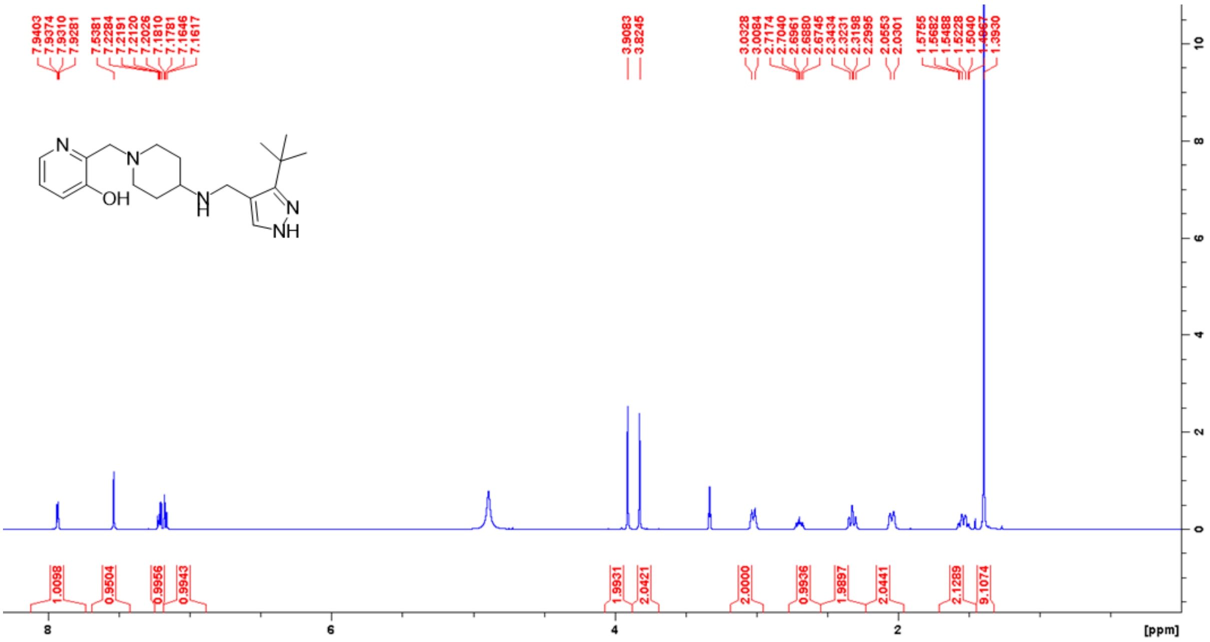

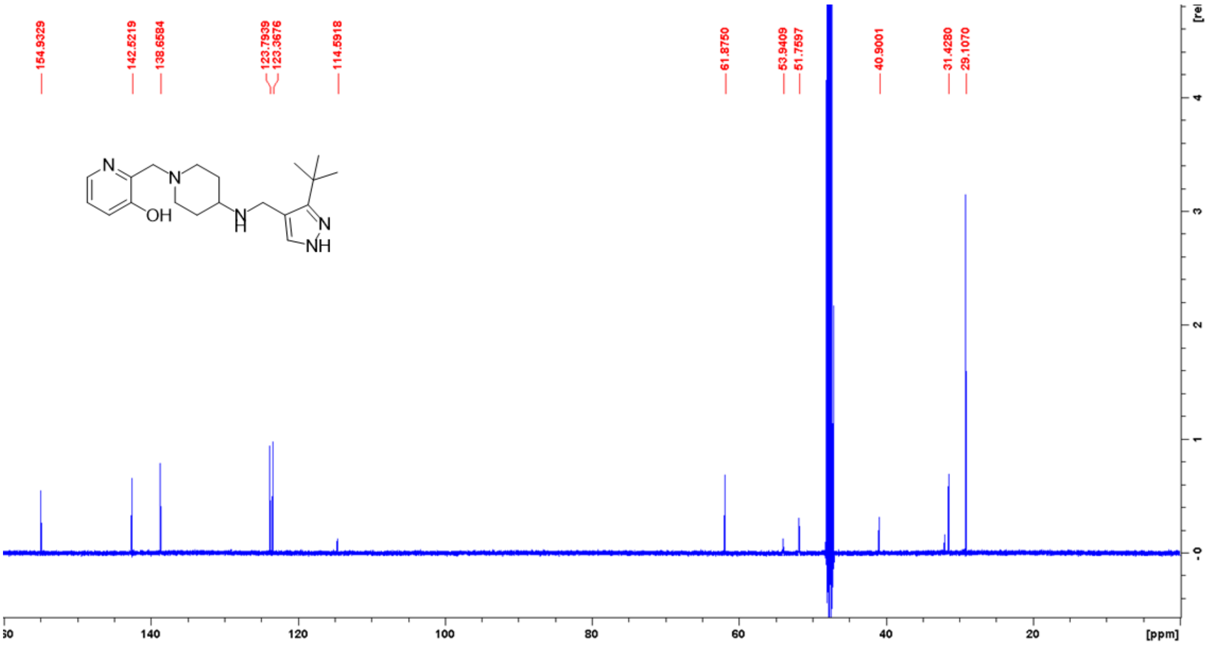

^{1}H and ^{13}C NMR spectra of compound 28 in MeOD

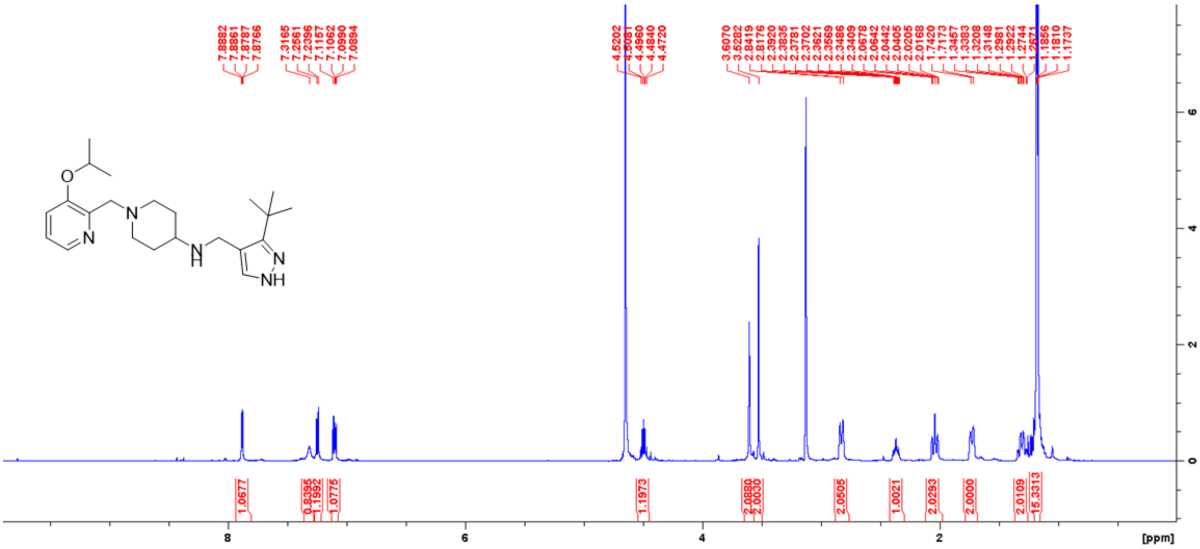

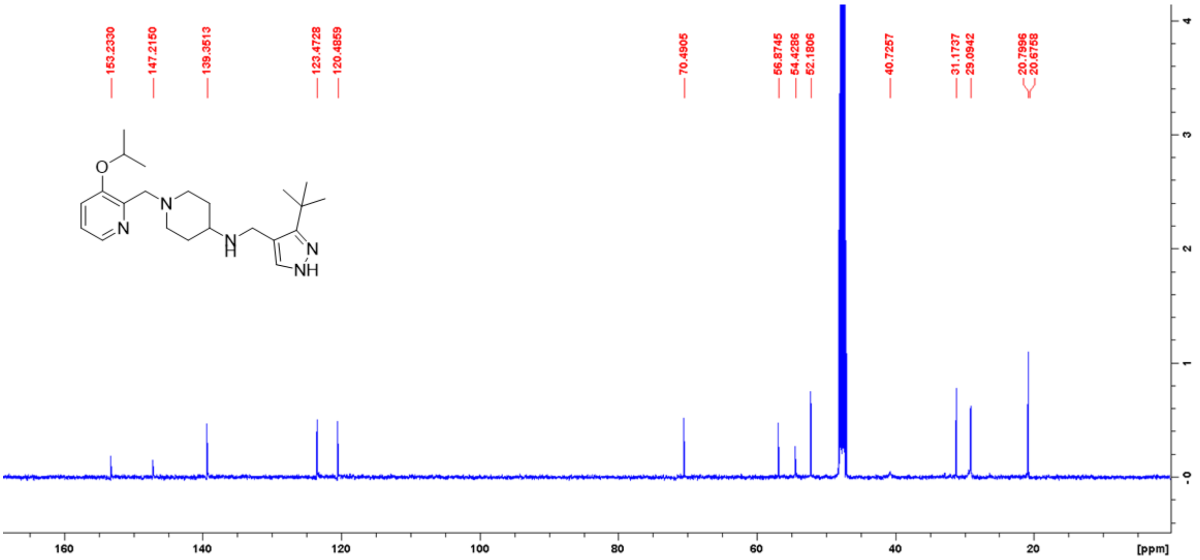

^{1}H and ^{13}C NMR spectra of compound 29 in MeOD

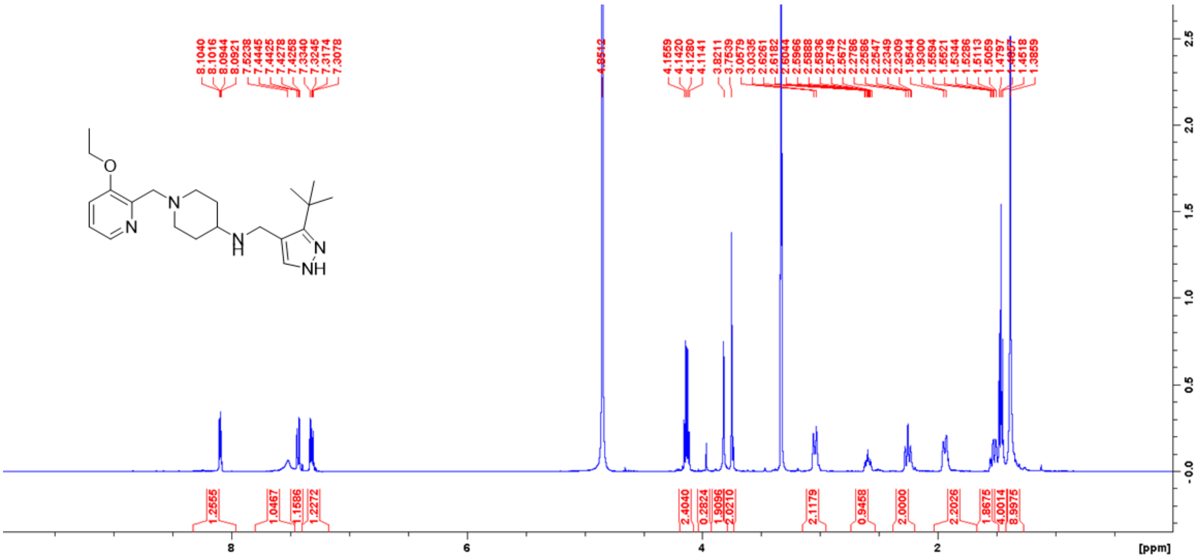

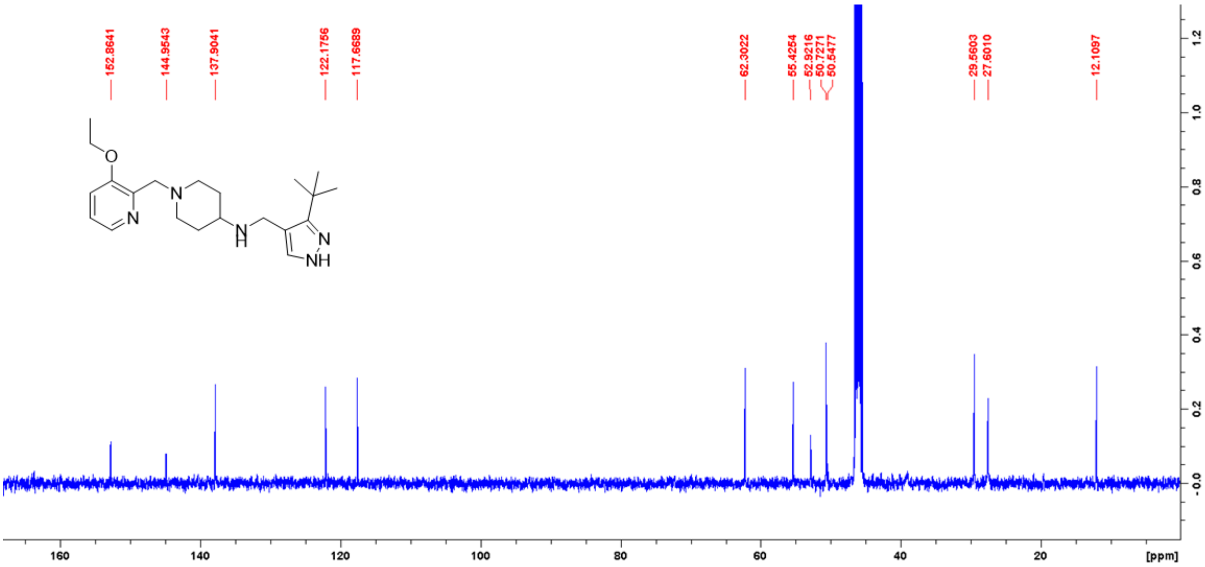

^{1}H and ^{13}C NMR spectra of compound 30 in MeOD

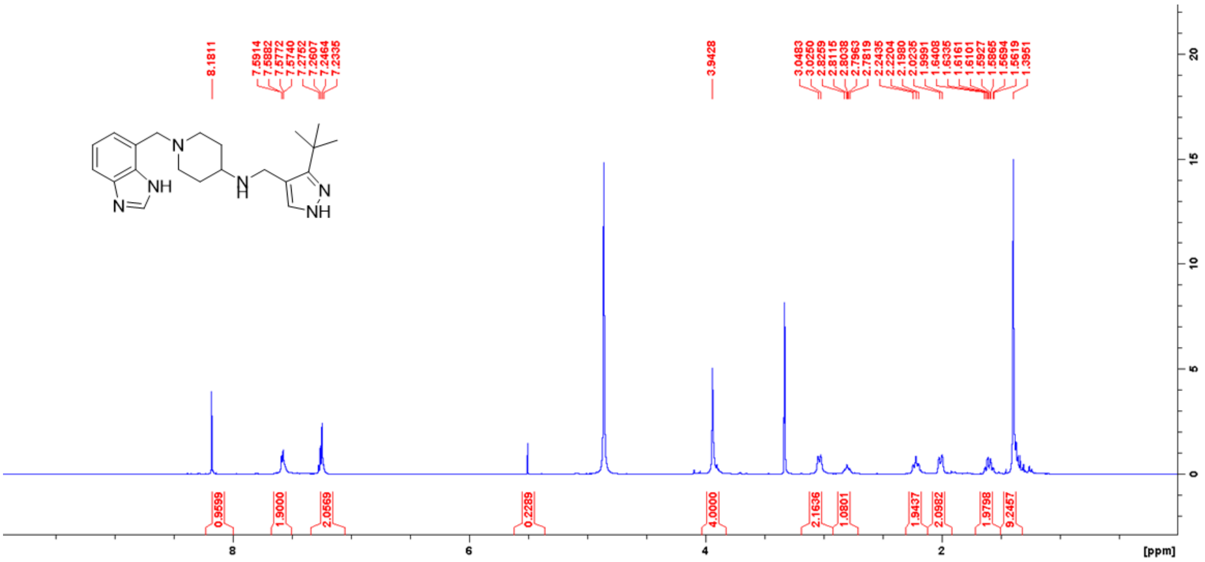

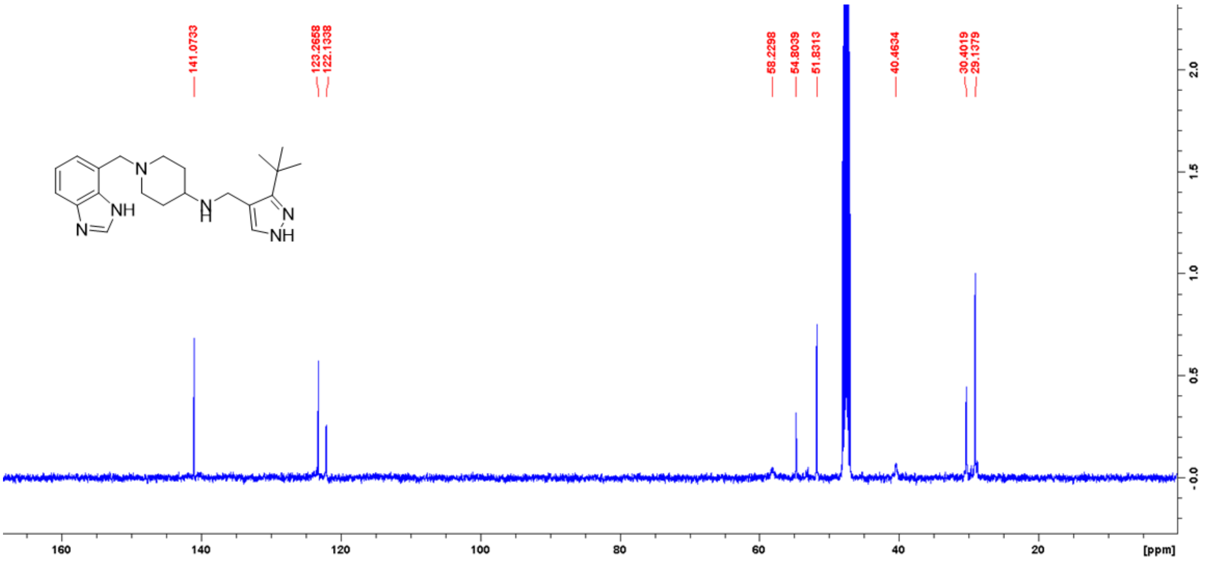

^{1}H and ^{13}C NMR spectra of compound 31 in MeOD

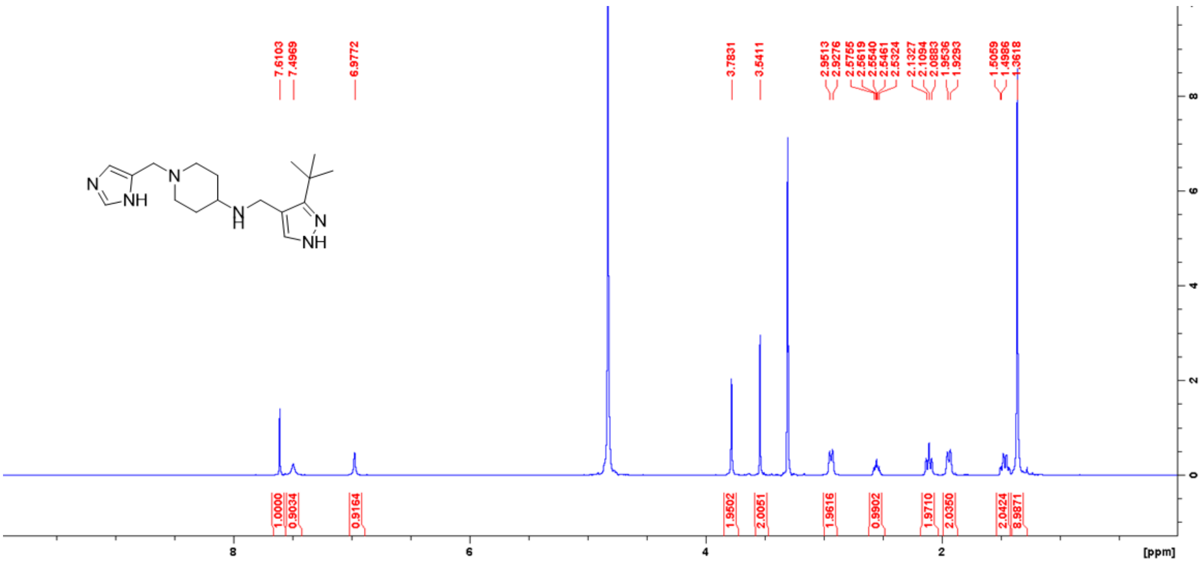

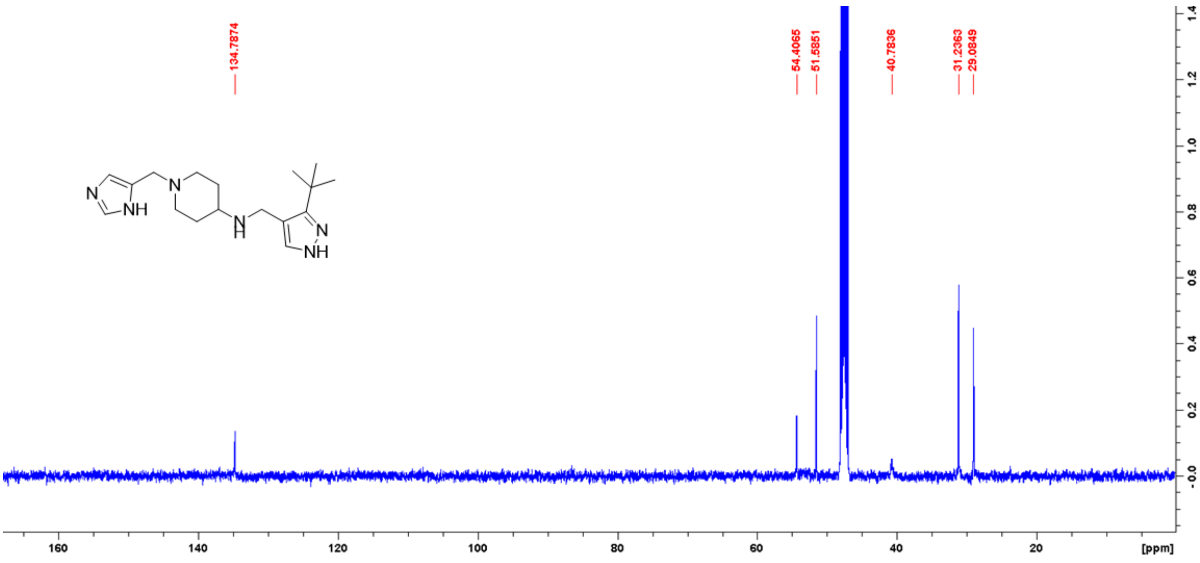

^{1}H and ^{13}C NMR spectra of compound 32 in MeOD

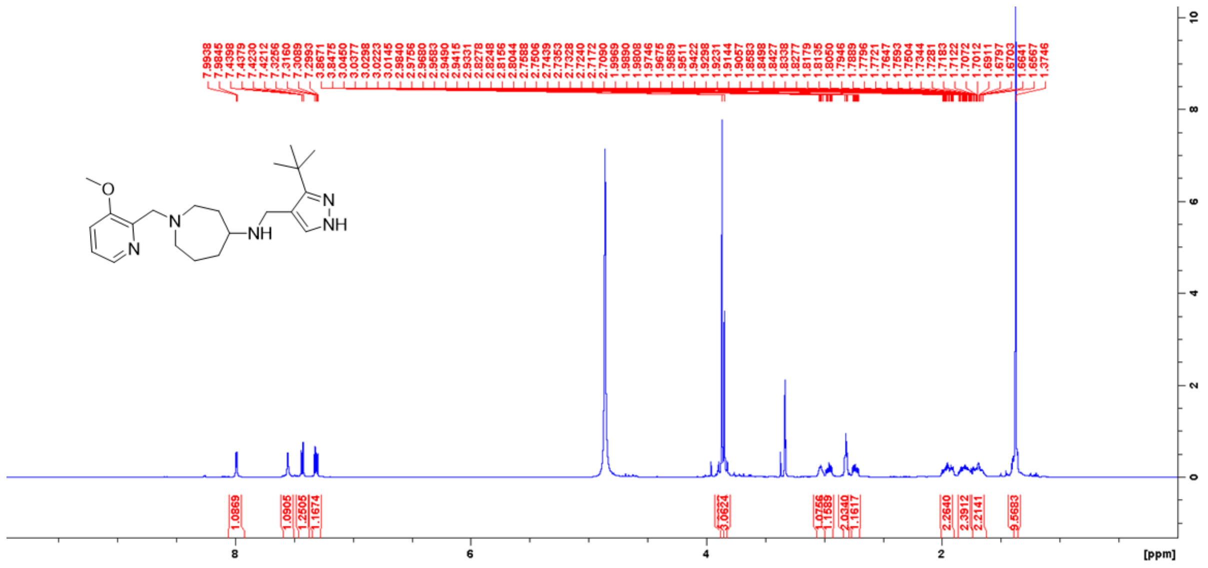

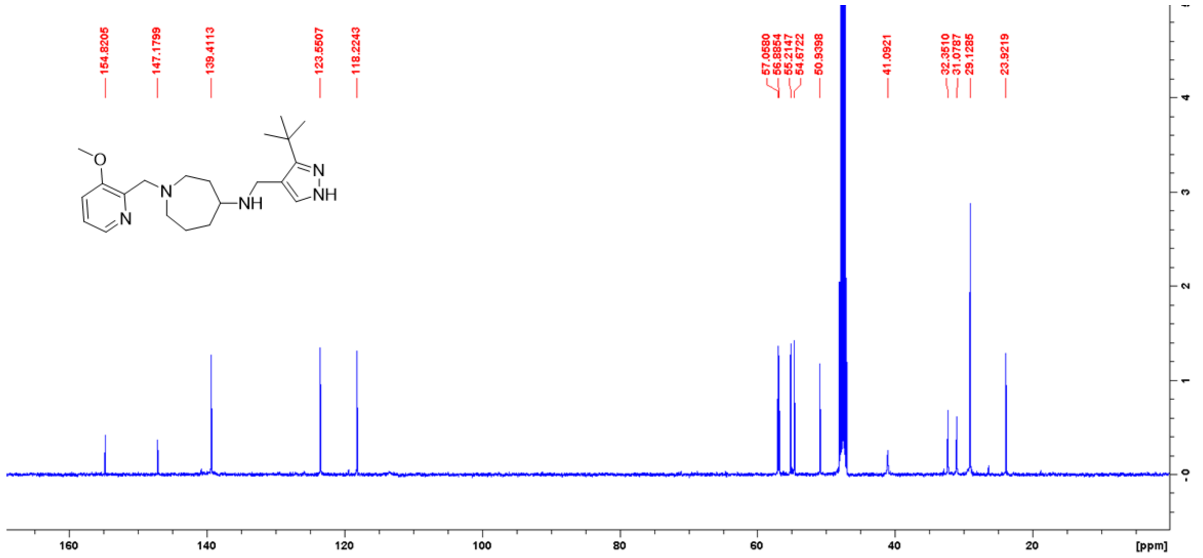

Supplement: Supplementary file 1 [file id6c00123_si_001.pdf]
